# Causal influence of plasma metabolites on age-related macular degeneration: A Mendelian randomization study

**DOI:** 10.1097/MD.0000000000039400

**Published:** 2024-09-13

**Authors:** Tao Wang, Chun Huang, Jinshuai Li, Xiangjian Wu, Xiaoyan Fu, Yimin Hu, Geping Wu, Chunfeng Yang, Sheng Chen

**Affiliations:** a Shenzhen Traditional Chinese Medicine Hospital, Shenzhen, Guangdong, China; b The Fourth Clinical Medical College, Guangzhou University of Chinese Medicine, Shenzhen, Guangdong, China; c School of Life Sciences, Beijing University of Chinese Medicine, Beijing, China.

**Keywords:** AMD, GWAS, Mendelian randomization analysis, metabolic pathway analysis, plasma metabolites

## Abstract

Using genome-wide association study data from European populations, this research clarifies the causal relationship between plasma metabolites and age-related macular degeneration (AMD) and employs Metabo Analyst 5.0 for enrichment analysis to investigate their metabolic pathways. Employing Mendelian randomization analysis, this study leveraged single nucleotide polymorphisms significantly associated with plasma metabolites as instrumental variables. This approach established a causal link between metabolites and AMD. Analytical methods such as inverse-variance weighted, Mendelian randomization-Egger, and weighted median were applied to validate causality. Mendelian Randomization Pleiotropy Residual Sum and Outlier was utilized for outlier detection and correction, and Cochran’s Q test was conducted to assess heterogeneity. To delve deeper into the metabolic characteristics of AMD, metabolic enrichment analysis was performed using Metabo Analyst 5.0. These combined methods provided a robust framework for elucidating the metabolic underpinnings of AMD. The 2-sample MR analysis, after meticulous screening, identified causal relationships between 88 metabolites and AMD. Of these, 16 metabolites showed a significant causal association. Following false discovery rate correction, 3 metabolites remained significantly associated, with androstenediol (3 beta, 17 beta) disulfate (2) exhibiting the most potent protective effect against AMD. Further exploration using Metabo Analyst 5.0 highlighted 4 metabolic pathways potentially implicated in AMD pathogenesis. This pioneering MR study has unraveled the causal connections between plasma metabolites and AMD. It identified several metabolites with a causal impact on AMD, with 3 maintaining significance after FDR correction. These insights offer robust causal evidence for future clinical applications and underscore the potential of these metabolites as clinical biomarkers in AMD screening, treatment, and prevention strategies.

## 1. Introduction

Age-related macular degeneration (AMD) is a prevalent chronic and degenerative eye condition that leads to central vision loss in individuals over 55, significantly impairing their quality of life. It emerges from a complex interplay of age, environmental, genetic, and metabolic factors.^[1]^ According to a meta-analysis by The Lancet, the number of AMD patients is projected to rise to 288 million by 2040,^[2]^ a figure that could be an underestimate considering increasing life expectancy and an aging global population. AMD is associated with heightened risks of depression and cognitive impairment^[3,4]^ and a 20% increase in all-cause mortality.^[5]^ The economic impact of AMD-induced blindness is substantial, encompassing both direct healthcare costs and indirect expenses such as caregiving and lost productivity. In 2020, the societal cost of AMD-related blindness in the US was estimated at 20 billion dollars, projected to triple by 2050.^[6]^ AMD typically progresses from early and intermediate stages, characterized by small and medium pigment deposits in the macula, to advanced stages. Advanced AMD is classified into 2 types: non-neovascular (dry) and neovascular (wet). Dry AMD mainly presents with geographic atrophy, leading to central blind spots and visual distortion, while wet AMD is marked by the growth of new blood vessels under or in the retina, causing severe visual distortion, large central blind spots, and a rapid decline in vision.^[7]^

Genome-wide association studies (GWAS) are extensively used to explore the relationships between genes and diseases. However, they fall short of elucidating the mechanisms underlying disease onset. In this context, metabolites serve as functional intermediates, shedding light on how genetic variations can impact metabolic processes and disease mechanisms.^[8]^ A notable study from China has identified 29 distinct metabolites across various metabolic pathways, including caffeine metabolism, biosynthesis of unsaturated fatty acids, and purine metabolism. These include 4-hydroxybenzoic acid, adrenic acid, and palmitic acid in plasma, which are promising candidates as biomarkers for neovascular age-related macular degeneration (nAMD). Changes in these metabolites may indicate metabolic imbalances in nAMD patients, offering insights into the disease’s molecular mechanism.^[9]^ For effective management of age-related macular degeneration (AMD), diagnostic tests must be both accessible and predictive of disease progression. Current research has delved into identifying serum and plasma biomarkers, particularly those associated with inflammation and lipid levels, due to their critical role in AMD’s pathogenesis.^[10–13]^ However, inconsistencies in research findings highlight the challenges in pinpointing reliable biological fluid biomarkers for AMD. Moreover, existing studies on AMD’s metabolome face limitations, including small sample sizes and the challenge of isolating dietary effects on AMD.^[14]^ These issues underscore the need for a deeper understanding of plasma metabolites’ roles in AMD.

Mendelian randomization (MR) analysis is a statistical method that leverages genetic variations as instrumental variables (IVs) to ascertain causal relationships between exposures and outcomes.^[15]^ Utilizing Mendel’s Second Law for random gene allocation, MR minimizes confounding factors, offering a clearer view of exposure effects on disease risk.^[16]^ This approach surpasses traditional observational studies by reducing confounder influence and avoiding reverse causality, providing more robust evidence in the absence of randomized trials. MR employs genetic variations as proxies for long-term exposures, thus circumventing common errors and biases in observational studies.^[17]^ With these premises, MR can be effectively used in large-scale studies to elucidate the causal relationship between plasma metabolites and AMD. Utilizing Metabo Analyst 5.0 for metabolic enrichment analysis, our study aims to discover metabolic pathways implicated in AMD’s pathogenesis. This research could significantly contribute to understanding the causal links between plasma metabolites and AMD, aiding in the development of new biomarkers and therapeutics, ultimately improving AMD patient care and prevention strategies.

## 2. Methods

### 2.1. Exposure data

We acquired comprehensive summary data on 1400 serum metabolites from a large-scale GWAS study conducted by Yiheng Chen et al. This dataset included 1091 blood metabolites and 309 metabolite ratios, sourced from the Canadian Longitudinal Study on Aging. The Canadian Longitudinal Study on Aging encompassed data from over 50,000 middle-aged and older Canadian participants. For the metabolomics analysis, ultra-high performance liquid chromatography-tandem mass spectrometry was utilized. In our study, we focused on 1091 metabolites that were measured in <50% of the samples. This subset included 850 known metabolites and 241 metabolites yet to be identified.^[18]^ These metabolites are found within 8 key metabolic pathways: lipids, amino acids, xenobiotic metabolism, nucleotides, cofactors and vitamins, carbohydrates, peptides, and energy. This distribution aligns with the classifications in the Kyoto Encyclopedia of Genes and Genomes (KEGG) database.^[19]^

### 2.2. Outcome data

We obtained comprehensive GWAS data on AMD from the FinnGen study. According to FinnGen’s ninth release, the dataset includes 8913 AMD patients and 348,936 controls. AMD is defined to encompass all types and stages of age-related macular degeneration (both dry and wet forms), based on central vision loss due to retinal degeneration. Exclusion criteria include other eye diseases such as diabetic retinopathy and retinal detachment. Detailed definitions and data sources can be found on Risteys (https://r9.finngen.fi/).^[20]^

### 2.3. Selection of IVs

For MR studies, 3 core assumptions must be met: the IV is significantly associated with the exposure; the IV is not associated with confounding factors between the exposure and outcome; and the IV affects the outcome solely through the exposure. For our analysis of 1400 serum and plasma metabolites, SNPs with *P* values < 5 × 10^−5^ were selected as IVs. Following the 1000 Genomes Project, we applied a linkage disequilibrium threshold of r^2 < 0.001 and a physical distance of 1000 kb. The F-statistic $\left(F=\frac{N-K-1}{K}\times \frac{R^{2}}{1-R^{2}}\right)$ was used to evaluate IV effectiveness, excluding those with an F-statistic < 10.^[21,22]^

### 2.4. MR analysis and sensitivity testing

The primary analysis method was inverse-variance weighted (IVW) method, aggregating weighted single nucleotide polymorphism causal effects for accurate causal inference.^[23,24]^ For sensitivity, the MR-Egger method estimated causal parameters and assessed overall causality.^[25]^ The weighted median (WM) method, assuming 50% of IVs are valid, was also used for robust causal effect estimation.^[26]^ MR pleiotropy residual sum and outlier (MR-PRESSO) detected and corrected horizontal pleiotropy outliers.^[27]^ MR-Egger intercept estimation tested for horizontal pleiotropy, with a non-zero intercept indicating its presence.^[28]^ Cochran’s Q test assessed heterogeneity.^[24,29]^ Leave-one-out sensitivity analysis removed 1 single nucleotide polymorphism at a time, enhancing the reliability of the analysis.

Consistency in *P* values and directions across the 3 MR methods indicated sufficient evidence for causality. We applied FDR correction to IVW *P* values; a q-value < 0.05 suggested a potential AMD candidate metabolite.^[30]^ Analyses were conducted using R version 4.3.1 (R Studio), with TwoSampleMR (version 0.5.7) and MR-PRESSO (version 1.0.0) for 2-sample MR analysis.

### 2.5. Metabolic Pathway Analysis

Metabolic enrichment analysis for AMD was conducted using Metabo Analyst 5.0 (https://www.metaboanalyst.ca/). By analyzing metabolite collections in KEGG and Small Molecule Pathway Database, we explored the metabolic characteristics of AMD.^[31]^

## 3. Results

### 3.1. MR analysis

**Figure 1.**
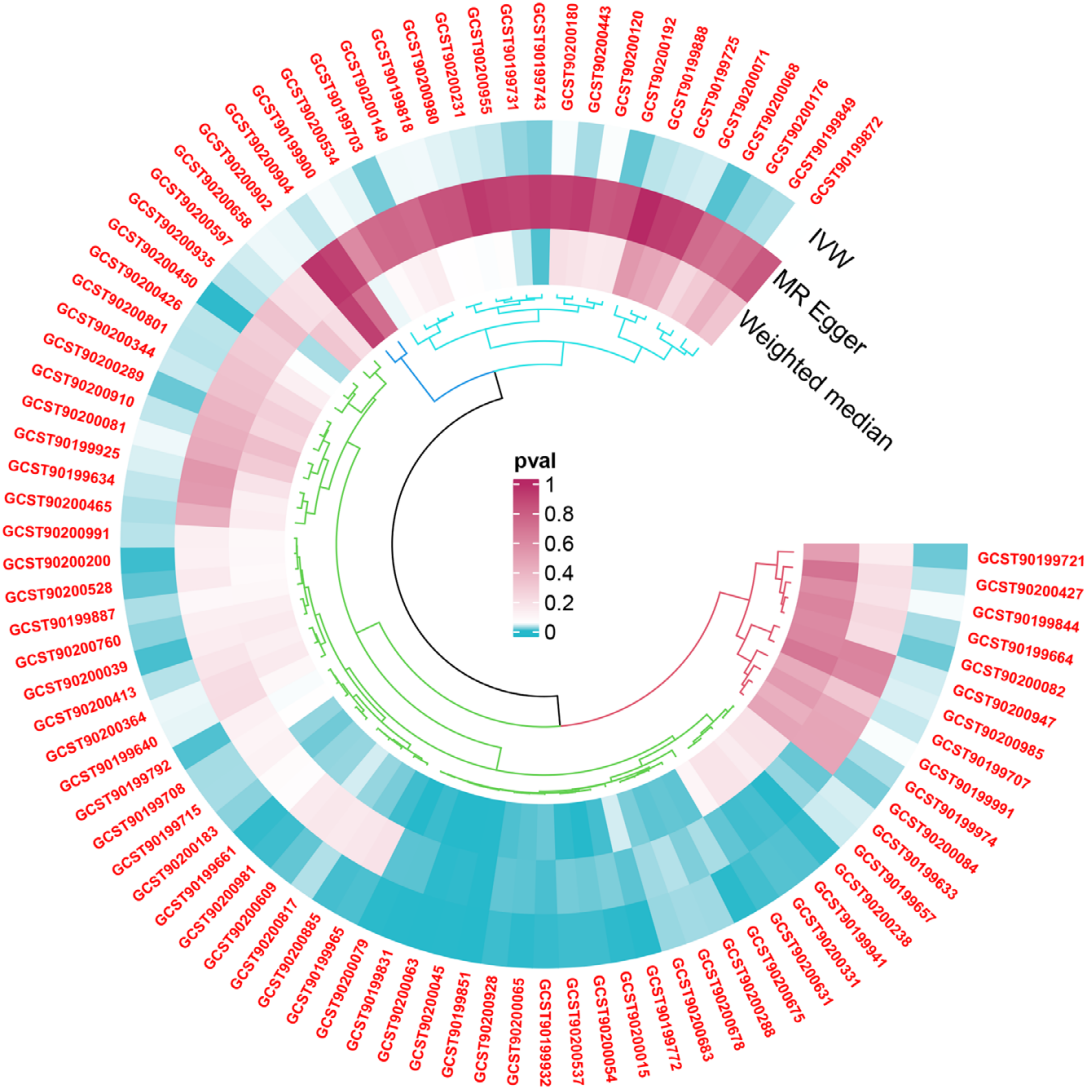
Circle plot illustrating the impact of metabolites identified by the IVW method on AMD. AMD = age-related macular degeneration, IVW = inverse-variance weighting, MR-Egger = Mendelian Randomization-Egger, WM = weighted median.

Our analysis encompassed 1400 metabolites, each represented by 11-82 SNPs, all with F-statistics >10 (Table S1, Supplemental Digital Content, http://links.lww.com/MD/N547). Utilizing the IVW method in MR analysis, we identified 88 serum and plasma metabolites associated with AMD (Fig. 1). Of these, 80 are known and 8 are unknown (Table S2, Supplemental Digital Content, http://links.lww.com/MD/N547). The comprehensive results of the IVW, MR-Egger, and Weighted Median methods for all 1400 metabolites are detailed in Tables S8 and S9, Supplemental Digital Content, http://links.lww.com/MD/N547.

**Figure 2.**
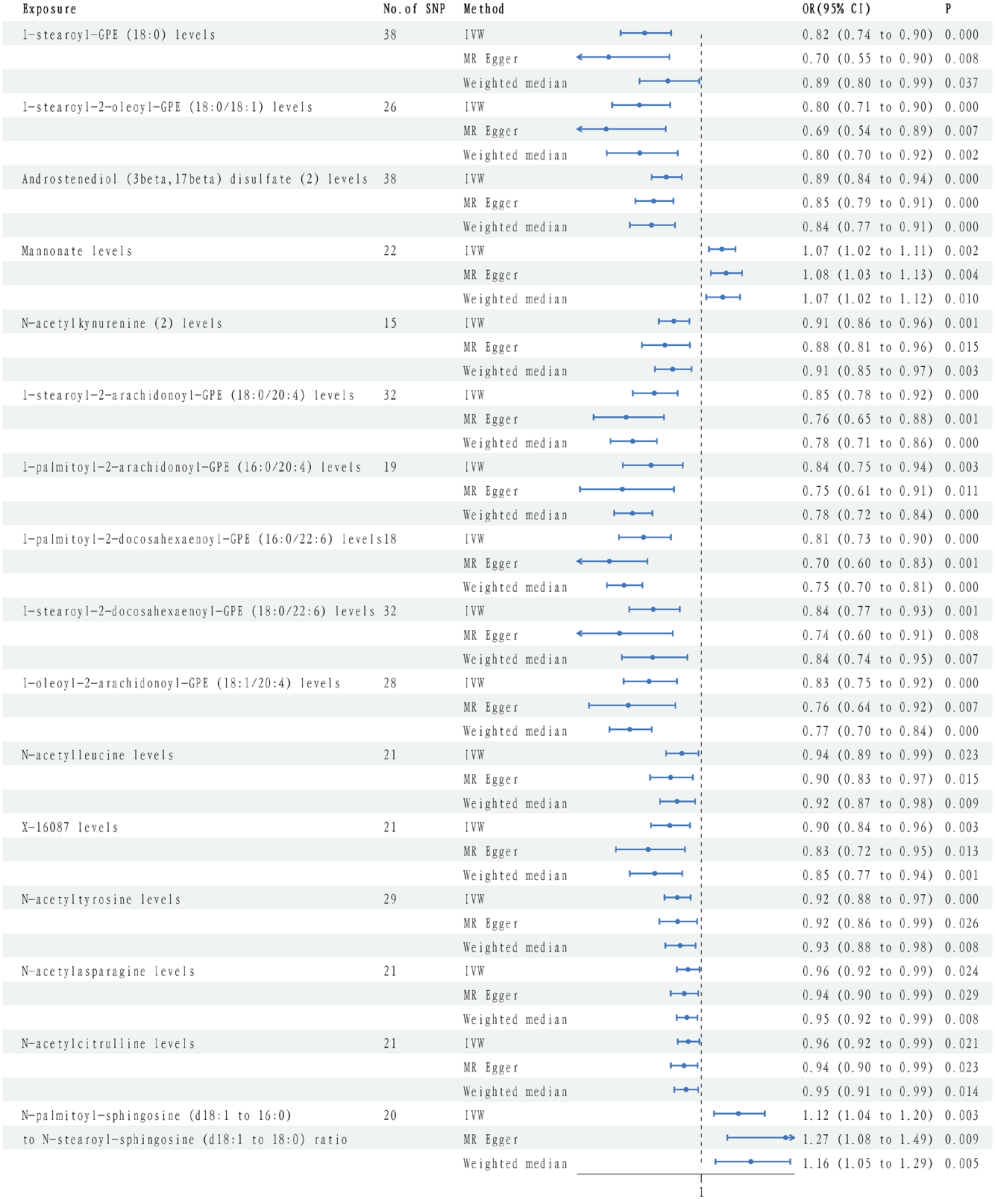
Forest plot of metabolite effects on AMD as determined by the IVW Method. AMD = age-related macular degeneration, CI = confidence interval, IVW = inverse-variance weighted method, OR = odds ratio.

As shown in Figure 2, after sensitivity analysis (MR-Egger and WM method), 16 metabolites showed significant *P* values and consistent directions across all 3 methods (Table S3, Supplemental Digital Content, http://links.lww.com/MD/N547), with 15 known and 1 unknown. These include 9 lipids, 5 amino acids, and 1 carbohydrate. Known AMD risk factors identified include the N-palmitoyl-sphingosine (d18: 1 to 16:0) to N-stearoyl-sphingosine (d18: 1 to 18:0) ratio (*P*: 0.0026, odds ratio [OR]: 1.12, 95% confidence interval [CI]: 1.04–1.20) and Mannonate levels (*P*: 0.0017, OR: 1.07, 95% CI: 1.02–1.11).

In contrast, several metabolites emerged as protective factors against AMD. Androstenediol (3 beta, 17 beta) disulfate (2) levels showed a significant protective effect (*P*: 0.000018, OR: 0.89, 95% CI: 0.84–0.94), followed by 1-stearoyl-GPE (18:0) (*P*: 0.000069, OR: 0.82, 95% CI: 0.74–0.90), 1-palmitoyl-2-docosahexaenoyl-GPE (16:0/22:6) (*P*: 0.000086, OR: 0.81, 95% CI: 0.73–0.90), 1-stearoyl-2-oleoyl-GPE (18:0/18:1) (*P*: 0.00018, OR: 0.80, 95% CI: 0.71–0.90), N-acetylkynurenine (2) (*P*: 0.00075, OR: 0.91, 95% CI: 0.86–0.96), 1-stearoyl-2-arachidonoyl-GPE (18:0/20:4) (*P*: 0.00015, OR: 0.85, 95% CI: 0.78–0.92), 1-palmitoyl-2-arachidonoyl-GPE (16:0/20:4) (*P*: 0.0026, OR: 0.84, 95% CI: 0.75–0.94), 1-stearoyl-2-docosahexaenoyl-GPE (18:0/22:6) (*P*: 0.00063, OR: 0.84, 95% CI: 0.78–0.93), 1-oleoyl-2-arachidonoyl-GPE (18:1/20:4) (*P*: 0.00046, OR: 0.83, 95% CI: 0.75–0.92), N-acetylleucine (*P*: 0.023, OR: 0.94, 95% CI: 0.89–0.99), N-acetyltyrosine (*P*: 0.00049, OR: 0.92, 95% CI: 0.88–0.97), N-acetylasparagine (*P*: 0.024, OR: 0.96, 95% CI: 0.92–0.99), and N-acetylcitrulline (*P*: 0.021, OR: 0.96, 95% CI: 0.92–0.99).

The metabolites did not show signs of heterogeneity or pleiotropy (Tables S4 and S5, Supplemental Digital Content, http://links.lww.com/MD/N547), All passed the MR-PRESSO test without evidence of horizontal pleiotropy (Table S6, Supplemental Digital Content, http://links.lww.com/MD/N547). As CFH and ARMS2 genes are known risk factors for AMD,^[32]^ it was necessary to exclude their influence. We searched for the SNPs related to these genes mentioned in the literature (e.g., rs1061170 and rs10490924) and found that these SNPs were not present in our dataset. This indicates that our selection of IVs did not include these known AMD risk factors, thereby excluding their influence.The leave-one-out plot (Figures S1–S16, Supplemental Digital Content, http://links.lww.com/MD/N546) confirms that excluding individual IVs did not significantly alter the results, reinforcing the analysis’s reliability. The scatter plots, funnel plots, and retention sensitivity analyses of the 16 metabolites are shown in Figures S17 to S48, Supplemental Digital Content, http://links.lww.com/MD/N546. Post-FDR correction, 3 metabolites with adjusted *P* values below 0.05 were identified: 1-stearoyl-GPE (18:0) levels, androstenediol (3 beta, 17 beta) disulfate (2) levels, and 1-palmitoyl-2-docosahexaenoyl-GPE (16:0/22:6) levels.

### 3.2. Metabolic pathway analysis

Utilizing Metabo Analyst 5.0, we selected 80 metabolites significant via the IVW method, with 19 identified for further metabolic pathway analysis to investigate their association with AMD. The analysis in Small Molecule Pathway Database and KEGG yielded specific results, as shown in Table 1. For more details on the metabolic pathways, refer to Table S7, Supplemental Digital Content, http://links.lww.com/MD/N547.

**Table 1:** Significant metabolic pathways involved in the pathogenesis of AMD.

Metabolic pathway	Metabolites Involved	*P* value	Database
Lysine degradation	5-hydroxylysine N(6),N(6),N(6)-trimethyl-L-lysine	0.01257	KEGG SMP
Glycerophospholipid metabolism	1-stearoyl-2-arachidonoyl-sn-glycero-3-phosphoethanolamine PC(18:1(9Z)/22:6(4Z,7Z,10Z,13Z,16Z,19Z))	0.025294	KEGG
Phenylalanine, tyrosine and tryptophan biosynthesis Linoleic acid metabolism	Tyrosine PC(18:1(9Z)/22:6(4Z,7Z,10Z,13Z,16Z,19Z))	0.028113 0.035028	KEGG SMP KEGG SMP

KEGG = Kyoto Encyclopedia of Genes and Genomes, SMPDB = small molecule pathway database.

## 4. Discussion

Our study successfully identified 88 metabolites with causal relationships to AMD, with 16 showing significant causal associations after rigorous sensitivity analysis. Notably, 3 metabolites—1-stearoyl-GPE (18:0), androstenediol (3 beta, 17 beta) disulfate (2), and 1-palmitoyl-2-docosahexaenoyl-GPE (16:0/22:6)—remained significant after FDR correction (*P* < .05). These metabolites are protective against AMD, with androstenediol (3 beta, 17 beta) disulfate (2) exhibiting the strongest protective effect. Our study found no evidence of pleiotropy or heterogeneity. Furthermore, we excluded the influence of CFH and ARMS2 gene variants. This research is pioneering in using metabolomics techniques and MR analysis to explore the potential causal impact of serum and plasma metabolites on AMD.

Our findings underscore the protective role of high levels of androstenediol (3 beta, 17 beta) monosulfate in slowing AMD progression. Notably, Androstenediol (3 beta, 17 beta) disulfate (2), a steroid sulfate derived from androstenediol, possesses both androgenic and estrogenic properties. Synthesized in the adrenal glands from DHEA via the Δ5 pathway,^[33]^ this compound functions in the central nervous system through both traditional androgen receptor (AR) signaling and alternative pathways. The traditional AR pathway, found in the fovea but not in the macula, and alternative pathways involving specific binding proteins, are present in various parts of the retina and are closely associated with sex hormones. Androgens, including metabolic derivatives of androstenediol, can also interact with GABAA receptors, which are located in areas of the central nervous system, including the retina.^[34]^ The neuroprotective role of androgens, such as androstenediol (3 beta, 17 beta) monosulfate, is evident in their influence on neural differentiation, survival, and development through the AR pathway.^[35]^ Testosterone, a derivative of androstanediol, has been shown to protect neurons from serum deprivation-induced cellular death, even when its conversion to estrogen is inhibited, a process that is suppressed by anti-androgen drugs like flutamide.^[36]^ This suggests that androstenediol (3 beta, 17 beta) monosulfate’s protective effect against AMD may be due to its neuroprotective efficacy. Androgens, by activating their receptors, can reduce oxidative stress, inflammation, and cell apoptosis, thus potentially preventing AMD.^[36,37]^

To date, no studies have directly linked 1-stearoyl-GPE (18:0) and 1-palmitoyl-2-docosahexaenoyl-GPE (16:0/22:6) levels with AMD. Both of these are glycerophosphoethanolamines, integral to phospholipid structures. Research involving the quantification of mixed extracts from the macula and surrounding retinal areas has investigated the relationship between lipofuscin accumulation and age in the retinal pigment epithelium. Notably, A2-glycerophosphoethanolamine, a type of retinoid acid, showed a 7.1-fold increase in these areas, although the increase was not statistically significant.^[38]^ This implies that A2-glycerophosphoethanolamine, despite being a retinoid acid, may not contribute to lipofuscin accumulation.^[39]^ In a separate study, a plasma metabolomics analysis was conducted on AMD patients and healthy controls using mass spectrometry. This study highlighted significant metabolite differences, particularly in lipid metabolism pathways. The glycerophospholipid pathway, in particular, exhibited significant metabolic variations in AMD patients. Glycerophospholipids, crucial for cell membrane structure, can impact membrane stability and function. Their levels may trigger processes like oxidative stress and influence the proliferation, differentiation, and migration of neuronal cells, ultimately affecting ocular cell health. The study also noted a correlation between reduced levels of diacylglycerol and phosphatidylcholine and neuronal cell membrane damage.^[40]^ Our research suggests that 1-stearoyl-GPE (18:0) and 1-palmitoyl-2-docosahexaenoyl-GPE (16:0/22:6) levels might also influence AMD through the glycerophosphate pathway. We found that both metabolites exhibit protective effects, potentially delaying AMD progression. However, the precise relationship and protective mechanisms of these metabolites in AMD are yet to be fully elucidated, highlighting a gap in experimental research in this area.

In our study, we performed a metabolic pathway analysis on 19 metabolites, uncovering several pathways associated with AMD. Notably, the KEGG highlighted Lysine degradation as the most significant metabolic pathway related to AMD, with a *P* value of 0.01257. This discovery underscores the potential importance of amino acid metabolism in AMD. Additionally, other relevant metabolic pathways were identified, including phenylalanine, tyrosine, and tryptophan biosynthesis (*P* = .028113), glycerophospholipid metabolism (*P* = .025294), and linoleic acid metabolism (*P* = .034). These findings suggest a complex interplay of various metabolic pathways in the progression and development of AMD, warranting further investigation to elucidate their specific roles and impacts.

The primary strength of our study is its pioneering approach of utilizing 2 extensive GWAS summary datasets to establish a causal link between plasma metabolites and AMD. This approach enabled us to identify 88 metabolites causally related to AMD, including 16 of significant relevance, with 3 maintaining significance post-FDR adjustment. This is particularly noteworthy considering that AMD often presents no symptoms in its early stages. Although optical coherence tomography and telemedicine technologies have shown potential in screening, their effectiveness is still under evaluation for specificity, sensitivity, and accuracy.^[41]^ Moreover,the high costs and advanced technology required for these methods may limit their applicability, especially in resource-constrained settings.

Our findings hold substantial promise for early AMD diagnosis by identifying potential biomarkers. These biomarkers not only improve diagnostic accuracy but also unveil biological pathways implicated in AMD, paving the way for novel treatment approaches. Additionally, understanding metabolite variations enables more personalized treatment plans, risk assessment, preventive measures, and monitoring of disease progression, thereby enriching our comprehension of AMD and enhancing current treatment and management strategies.

Furthermore, our study employed IVs with F-statistics exceeding 10 to mitigate weak instrument bias. We also incorporated multiple methods to address heterogeneity and pleiotropy, bolstering the reliability of our results. FDR testing further reinforced this, adjusting the significance threshold to minimize false positives and elevate the credibility of our findings.

However, the study is not without limitations. Despite employing MR-egger and MR-presso methods to account for pleiotropy, potential confounding factors like nutritional status and smoking habits could still introduce biases.^[42]^ Additionally, while we identified 3 metabolites associated with AMD, the underlying biological mechanisms of how plasma metabolites influence AMD require further exploration. Last, since our data primarily pertains to European populations, the generalizability of our results to other ethnicities remains uncertain, highlighting the need for future studies to encompass a broader demographic range.

## 5. Conclusions

This study represents a groundbreaking MR investigation into the causal relationship between plasma metabolites and AMD. We successfully identified several metabolites with causal effects on AMD, with 3 demonstrating significant associations even after FDR correction. These findings are anticipated to contribute high-quality causal evidence to clinical practice, laying a foundation for using these metabolites as clinical circulating biomarkers in the screening, treatment, and prevention of AMD.

## Acknowledgments

Our gratitude extends to all the studies that provided public GWAS summary data, as well as the researchers and participants involved in these GWAS studies.

## Author contributions

**Conceptualization:** Tao Wang, Chun Huang, Sheng Chen.

**Data curation:** Tao Wang, Chun Huang, Jinshuai Li, Sheng Chen.

**Formal analysis:** Tao Wang.

**Methodology:** Tao Wang, Chun Huang.

**Resources:** Tao Wang, Chun Huang, Jinshuai Li, Xiaoyan Fu, Chunfeng Yang.

**Writing – original draft:** Tao Wang.

**Visualization:** Chun Huang, Xiaoyan Fu, Geping wu.

**Software:** Jinshuai Li, Xiangjian Wu, Xiaoyan Fu, Yimin Hu, Chunfeng Yang

**Investigation:** Xiangjian Wu

**Validation:** Xiangjian Wu, Xiaoyan Fu, Yimin Hu, Geping Wu

**Supervision:** Yimin Hu, Geping wu, Chunfeng Yang

**Funding acquisition:** Sheng Chen

**Writing – review and editing:** Sheng Chen.

## Supplementary Material


