# Supplementary material for: Causal influence of plasma metabolites on age-related macular degeneration: A Mendelian randomization study
Source: Medicine (Baltimore). 2024 Sep 13;103(37):e39400. doi: 10.1097/MD.0000000000039400 (PMC11404906; doi:10.1097/MD.0000000000039400)
Supplement: Supplementary file 2 [file medi-103-e39400-s002.pdf]

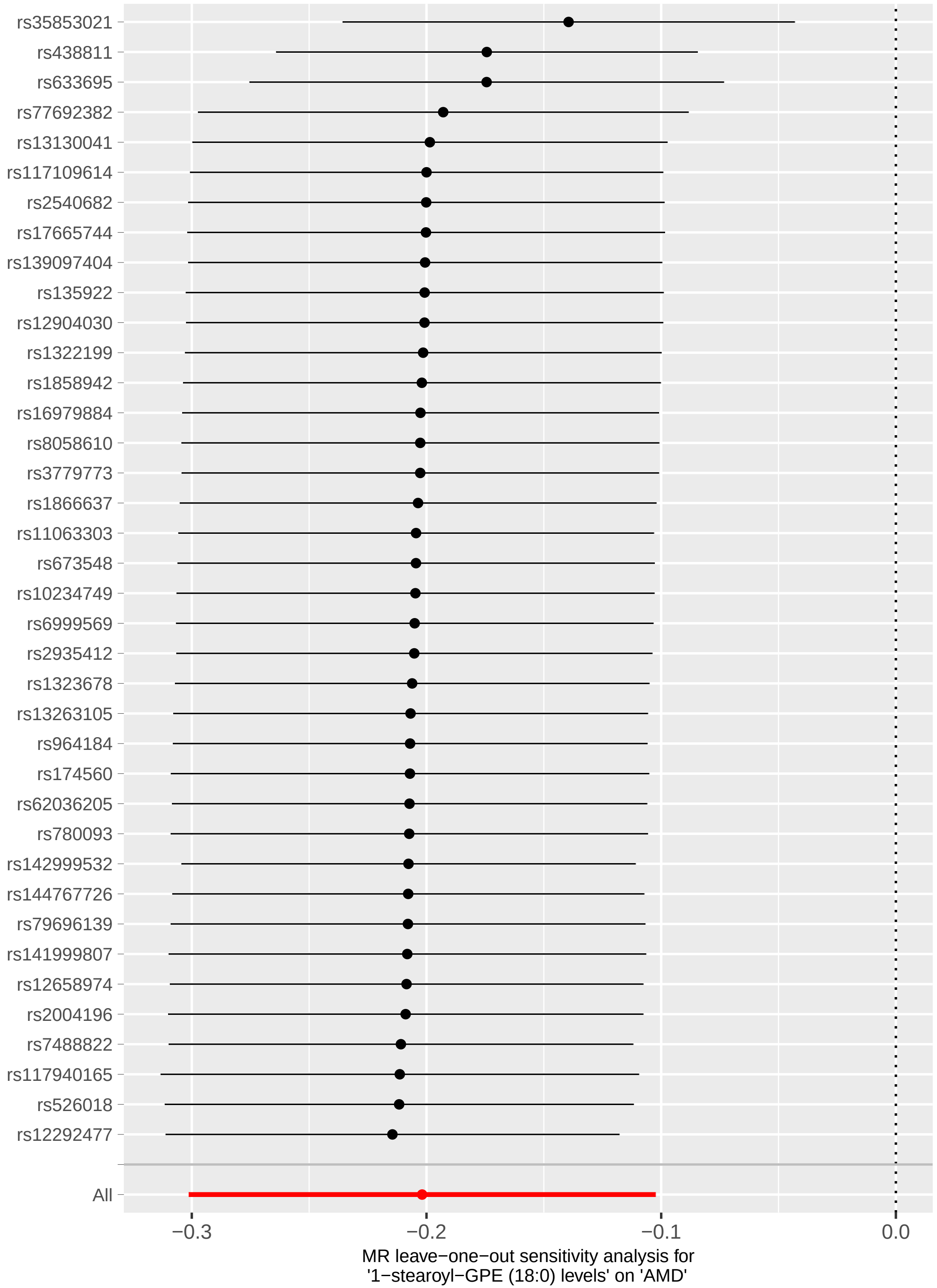

rs35853021
rs438811
rs633695
rs77692382
rs13130041
rs117109614
rs2540682
rs17665744
rs139097404
rs135922
rs12904030
rs1322199
rs1858942
rs16979884
rs8058610
rs3779773
rs1866637
rs11063303
rs673548
rs10234749
rs6999569
rs2935412
rs1323678
rs13263105
rs964184
rs174560
rs62036205
rs780093
rs142999532
rs144767726
rs79696139
rs141999807
rs12658974
rs2004196
rs7488822
rs117940165
rs526018
rs12292477
All
−0.3
−0.2
−0.1
0.0
MR leave−one−out sensitivity analysis for
'1−stearoyl−GPE (18:0) levels' on 'AMD'

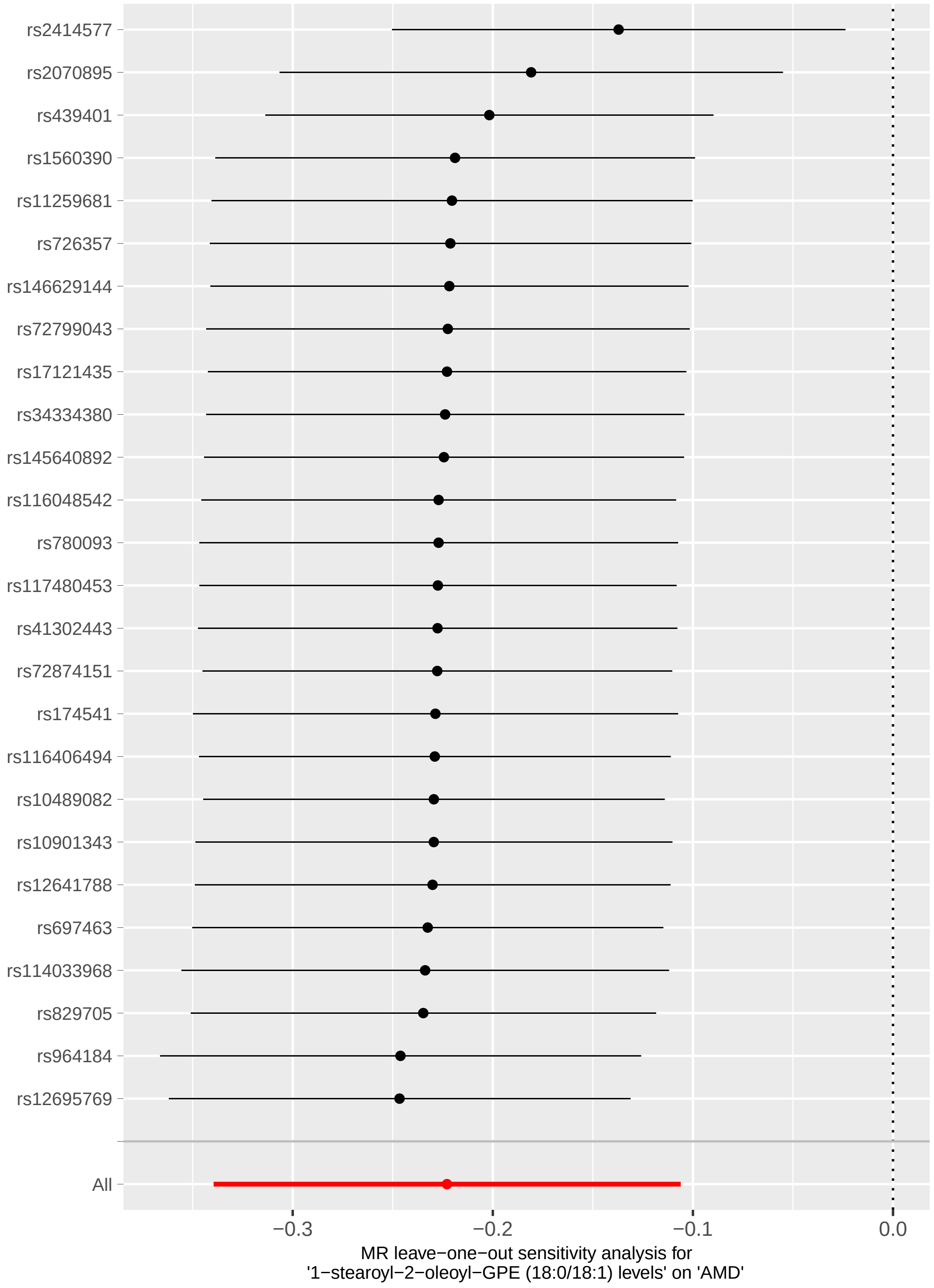

rs2414577
rs2070895
rs439401
rs1560390
rs11259681
rs726357
rs146629144
rs72799043
rs17121435
rs34334380
rs145640892
rs116048542
rs780093
rs117480453
rs41302443
rs72874151
rs174541
rs116406494
rs10489082
rs10901343
rs12641788
rs697463
rs114033968
rs829705
rs964184
rs12695769
All
−0.3
−0.2
−0.1
0.0
MR leave−one−out sensitivity analysis for
'1−stearoyl−2−oleoyl−GPE (18:0/18:1) levels' on 'AMD'

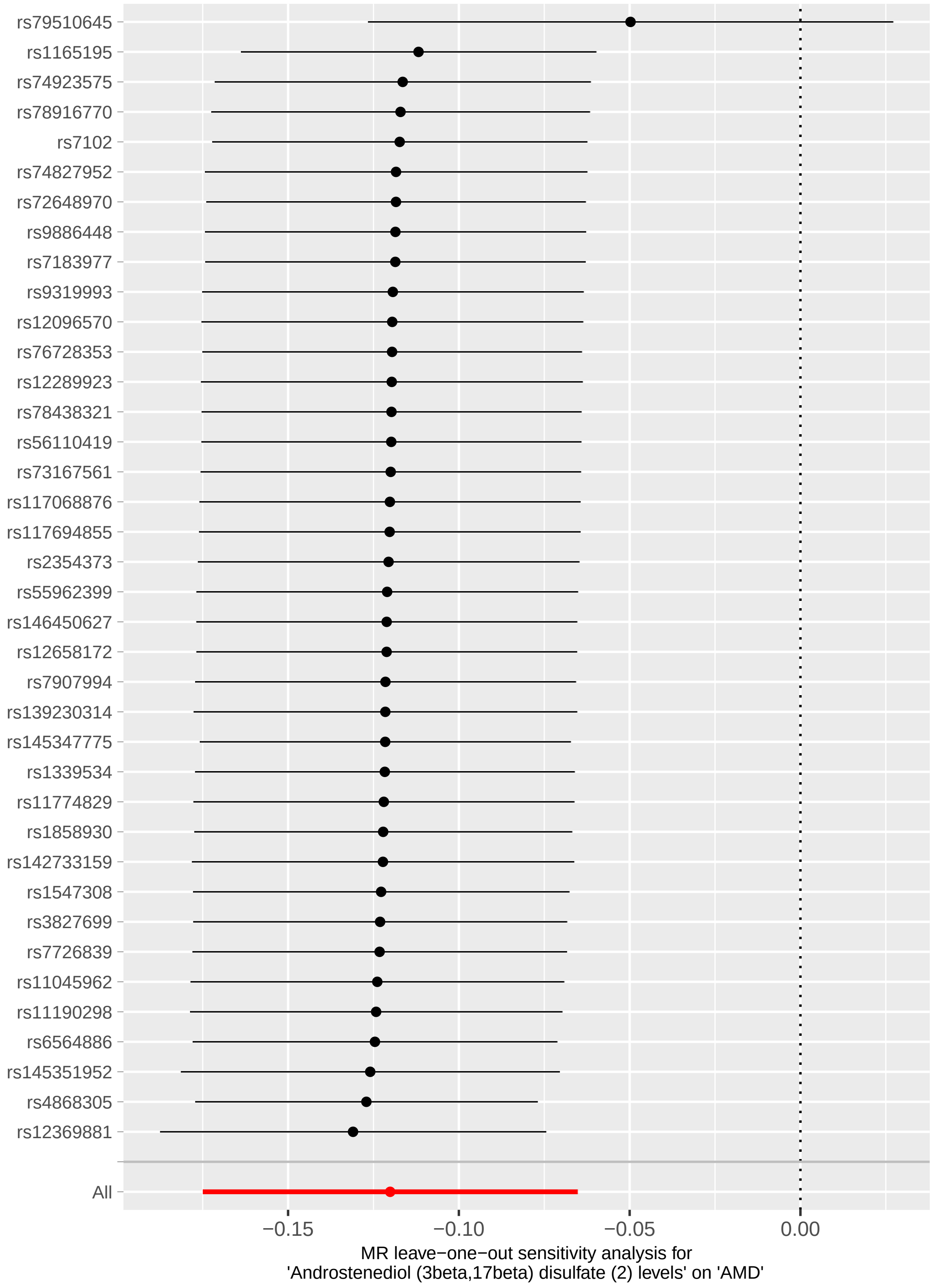

rs79510645
rs1165195
rs74923575
rs78916770
rs7102
rs74827952
rs72648970
rs9886448
rs7183977
rs9319993
rs12096570
rs76728353
rs12289923
rs78438321
rs56110419
rs73167561
rs117068876
rs117694855
rs2354373
rs55962399
rs146450627
rs12658172
rs7907994
rs139230314
rs145347775
rs1339534
rs11774829
rs1858930
rs142733159
rs1547308
rs3827699
rs7726839
rs11045962
rs11190298
rs6564886
rs145351952
rs4868305
rs12369881
All
−0.15
−0.10
−0.05
0.00
MR leave−one−out sensitivity analysis for
'Androstenediol (3beta,17beta) disulfate (2) levels' on 'AMD'

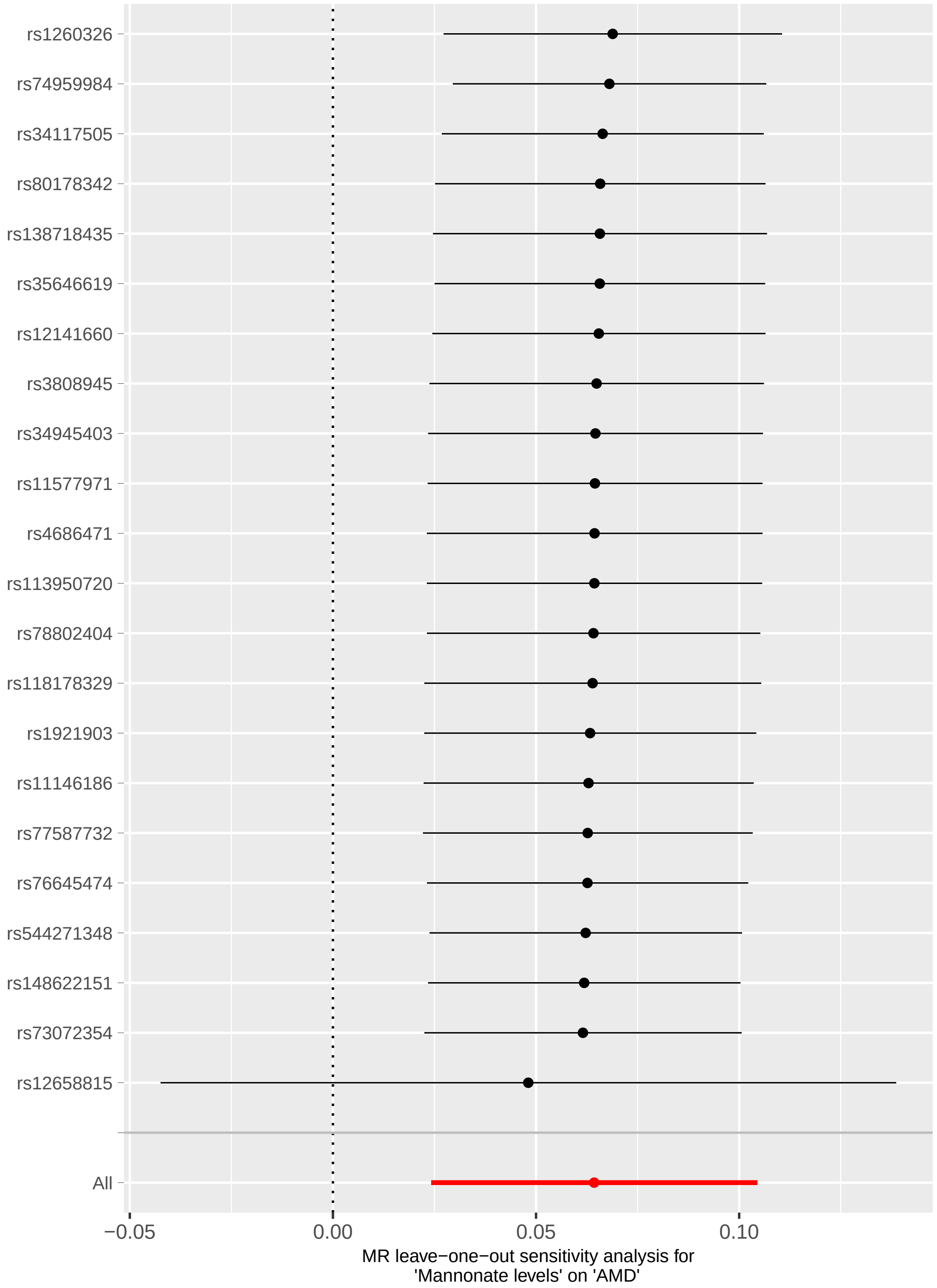
rs1260326
rs74959984
rs34117505
rs80178342
rs138718435
rs35646619
rs12141660
rs3808945
rs34945403
rs11577971
rs4686471
rs113950720
rs78802404
rs118178329
rs1921903
rs11146186
rs77587732
rs76645474
rs544271348
rs148622151
rs73072354
rs12658815
All
−0.05
0.00
0.05
0.10
MR leave−one−out sensitivity analysis for
'Mannonate levels' on 'AMD'

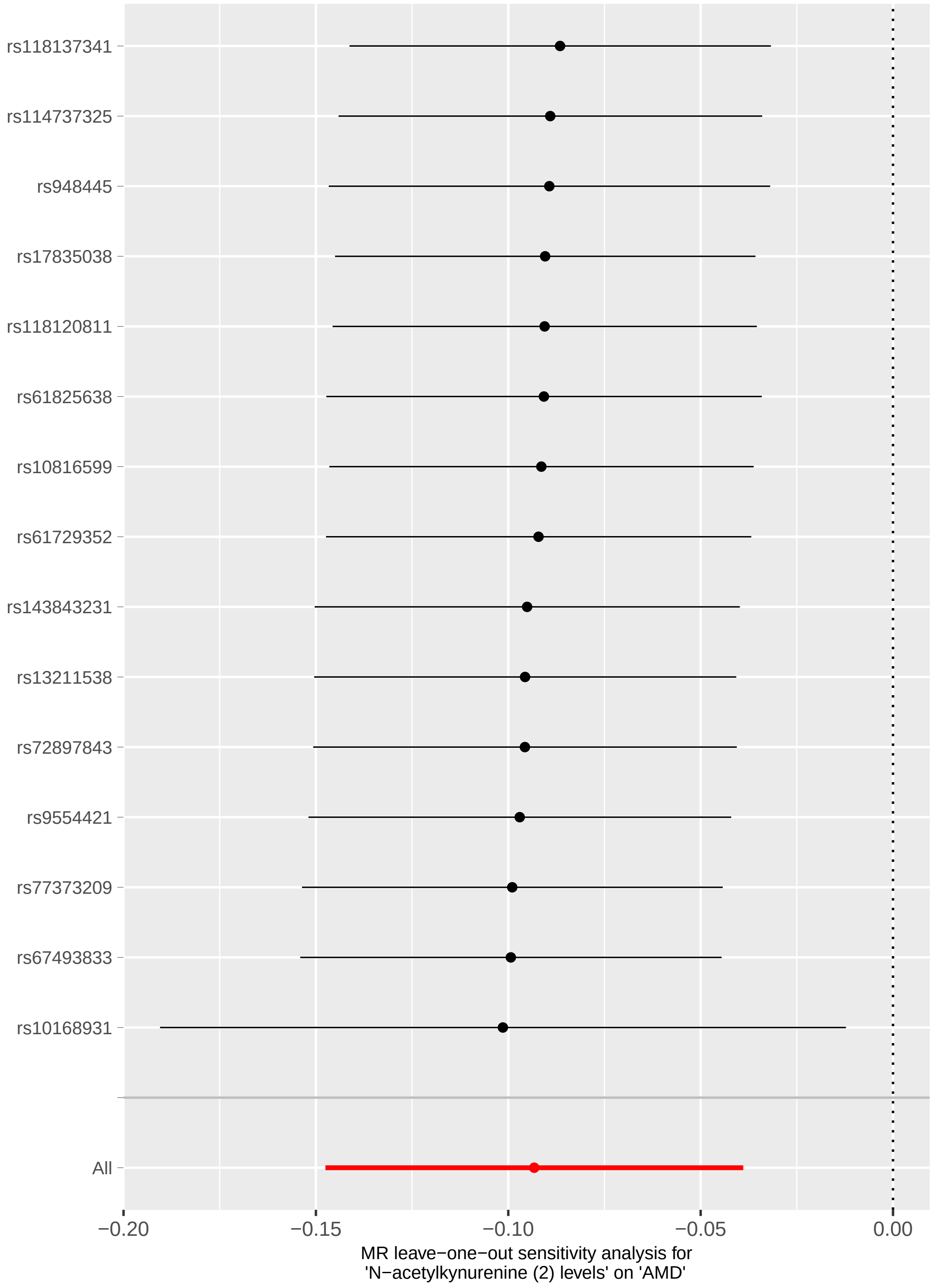
rs118137341
rs114737325
rs948445
rs17835038
rs118120811
rs61825638
rs10816599
rs61729352
rs143843231
rs13211538
rs72897843
rs9554421
rs77373209
rs67493833
rs10168931
All
−0.20
−0.15
−0.10
−0.05
0.00
MR leave−one−out sensitivity analysis for
'N−acetylkynurenine (2) levels' on 'AMD'

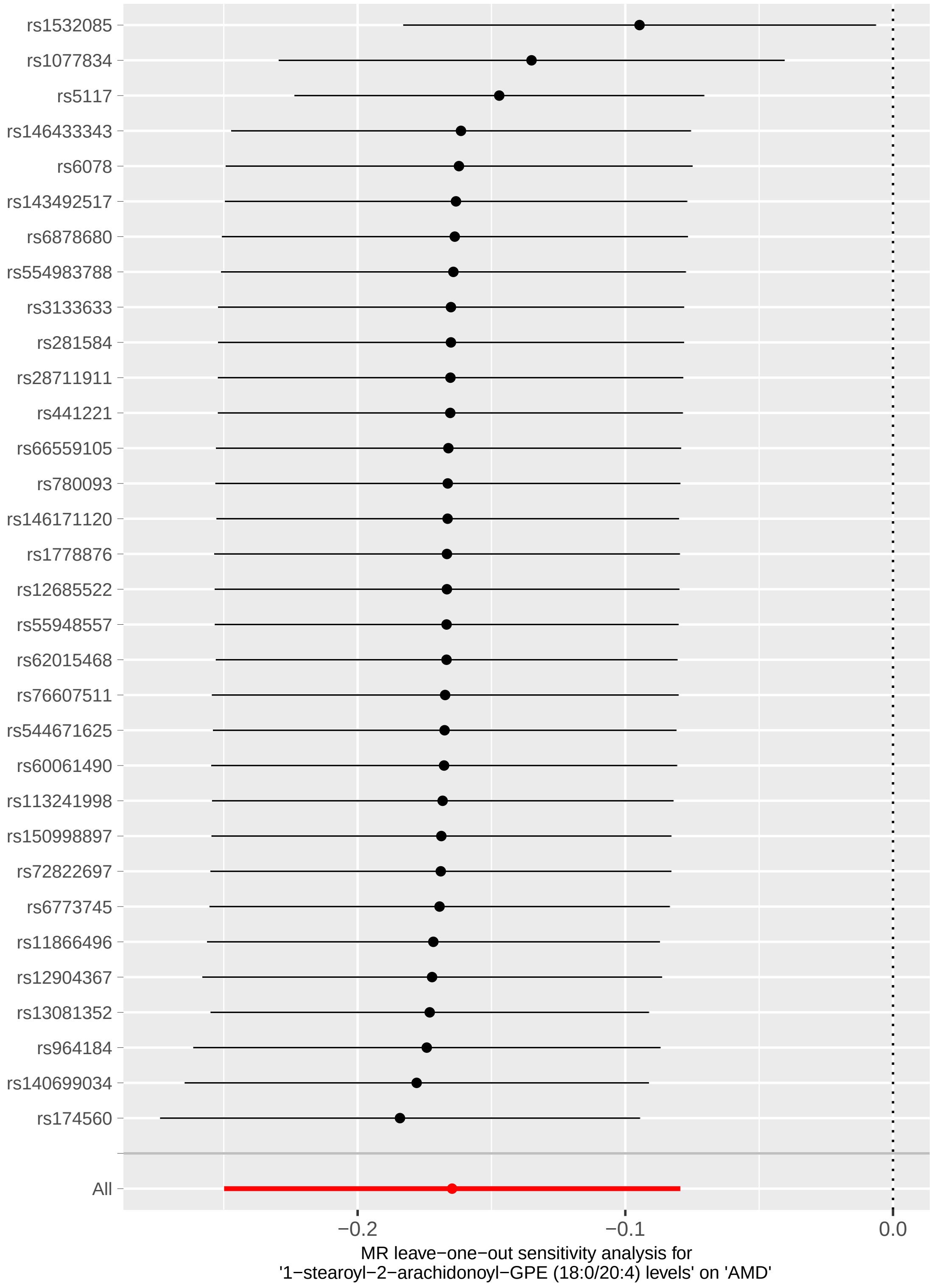
rs1532085
rs1077834
rs5117
rs146433343
rs6078
rs143492517
rs6878680
rs554983788
rs3133633
rs281584
rs28711911
rs441221
rs66559105
rs780093
rs146171120
rs1778876
rs12685522
rs55948557
rs62015468
rs76607511
rs544671625
rs60061490
rs113241998
rs150998897
rs72822697
rs6773745
rs11866496
rs12904367
rs13081352
rs964184
rs140699034
rs174560
All
−0.2
−0.1
0.0
MR leave−one−out sensitivity analysis for
'1−stearoyl−2−arachidonoyl−GPE (18:0/20:4) levels' on 'AMD'

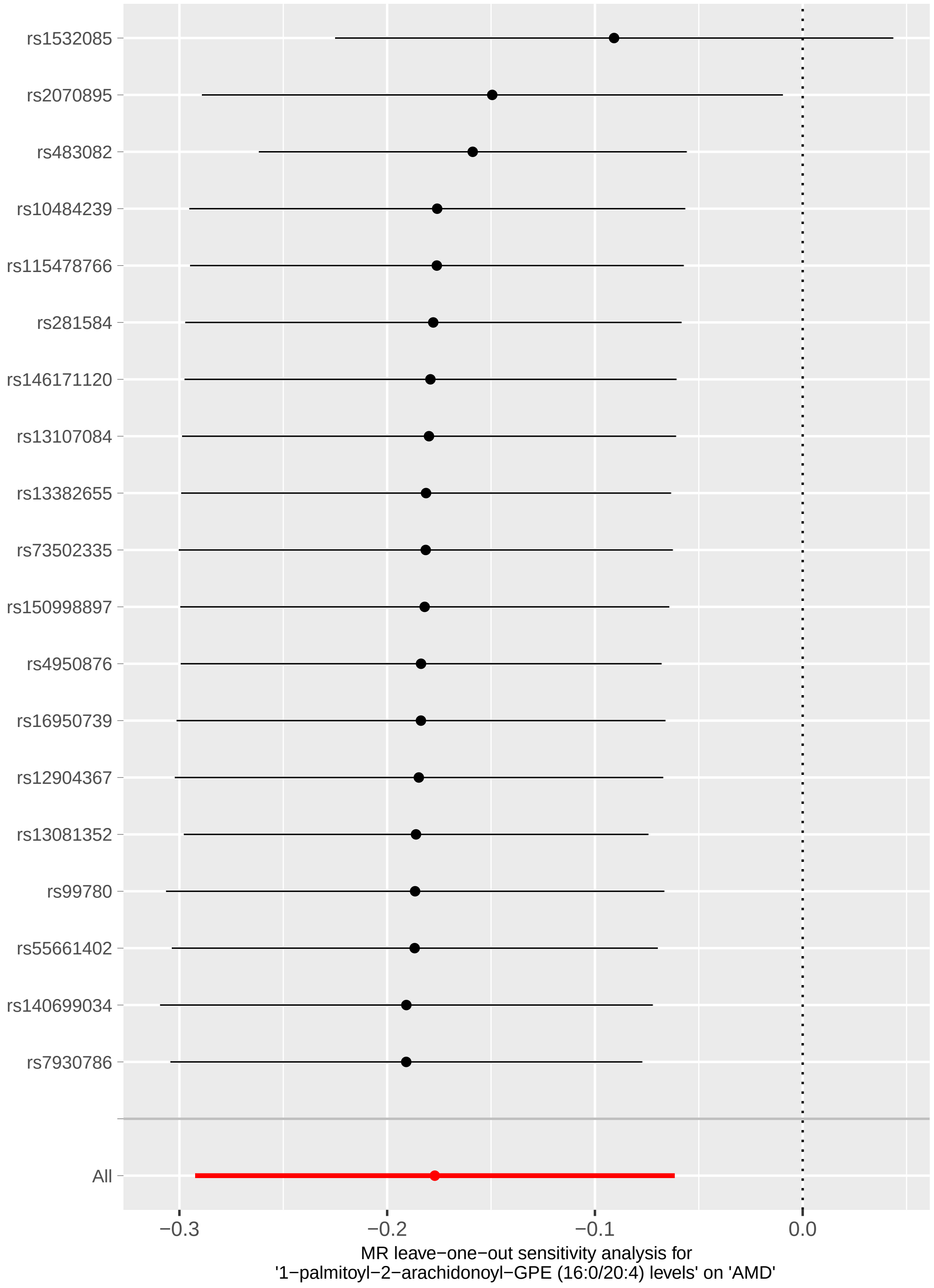
rs1532085
rs2070895
rs483082
rs10484239
rs115478766
rs281584
rs146171120
rs13107084
rs13382655
rs73502335
rs150998897
rs4950876
rs16950739
rs12904367
rs13081352
rs99780
rs55661402
rs140699034
rs7930786
All
−0.3
−0.2
−0.1
0.0
MR leave−one−out sensitivity analysis for
'1−palmitoyl−2−arachidonoyl−GPE (16:0/20:4) levels' on 'AMD'

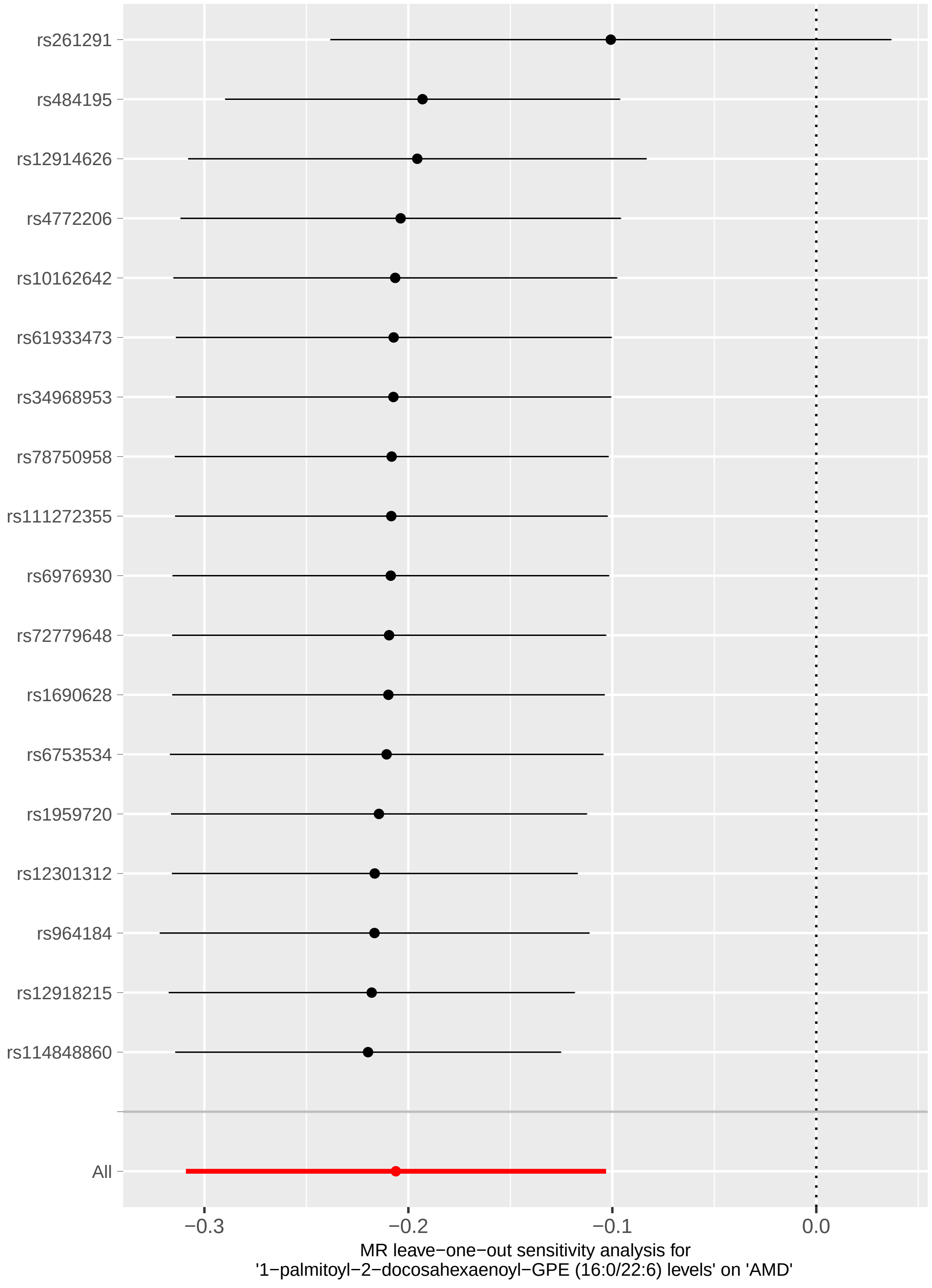
rs261291
rs484195
rs12914626
rs4772206
rs10162642
rs61933473
rs34968953
rs78750958
rs111272355
rs6976930
rs72779648
rs1690628
rs6753534
rs1959720
rs12301312
rs964184
rs12918215
rs114848860
All
−0.3
−0.2
−0.1
0.0
MR leave−one−out sensitivity analysis for
'1−palmitoyl−2−docosahexaenoyl−GPE (16:0/22:6) levels' on 'AMD'

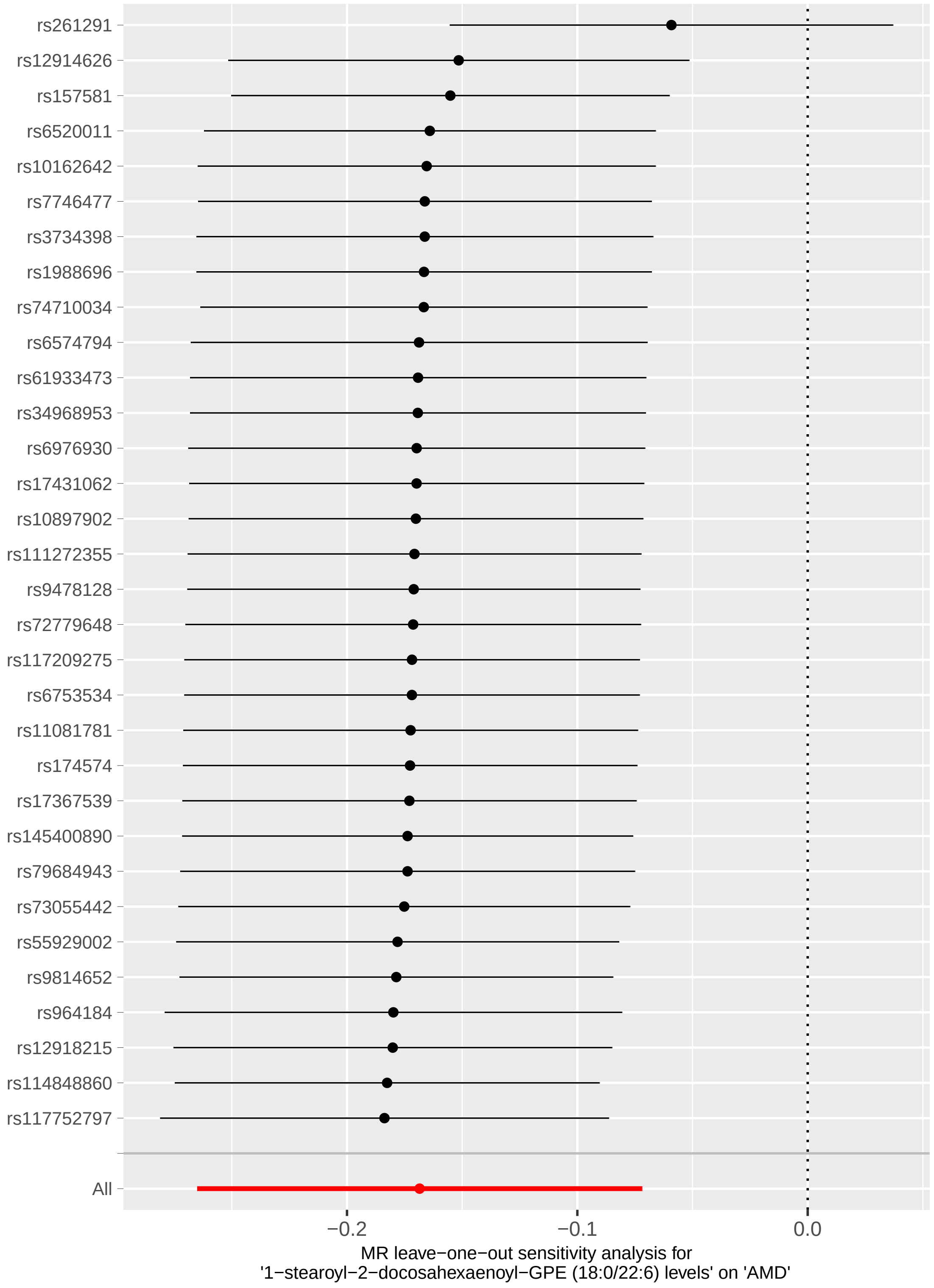

rs261291
rs12914626
rs157581
rs6520011
rs10162642
rs7746477
rs3734398
rs1988696
rs74710034
rs6574794
rs61933473
rs34968953
rs6976930
rs17431062
rs10897902
rs111272355
rs9478128
rs72779648
rs117209275
rs6753534
rs11081781
rs174574
rs17367539
rs145400890
rs79684943
rs73055442
rs55929002
rs9814652
rs964184
rs12918215
rs114848860
rs117752797
All
−0.2
−0.1
0.0
MR leave−one−out sensitivity analysis for
'1−stearoyl−2−docosahexaenoyl−GPE (18:0/22:6) levels' on 'AMD'

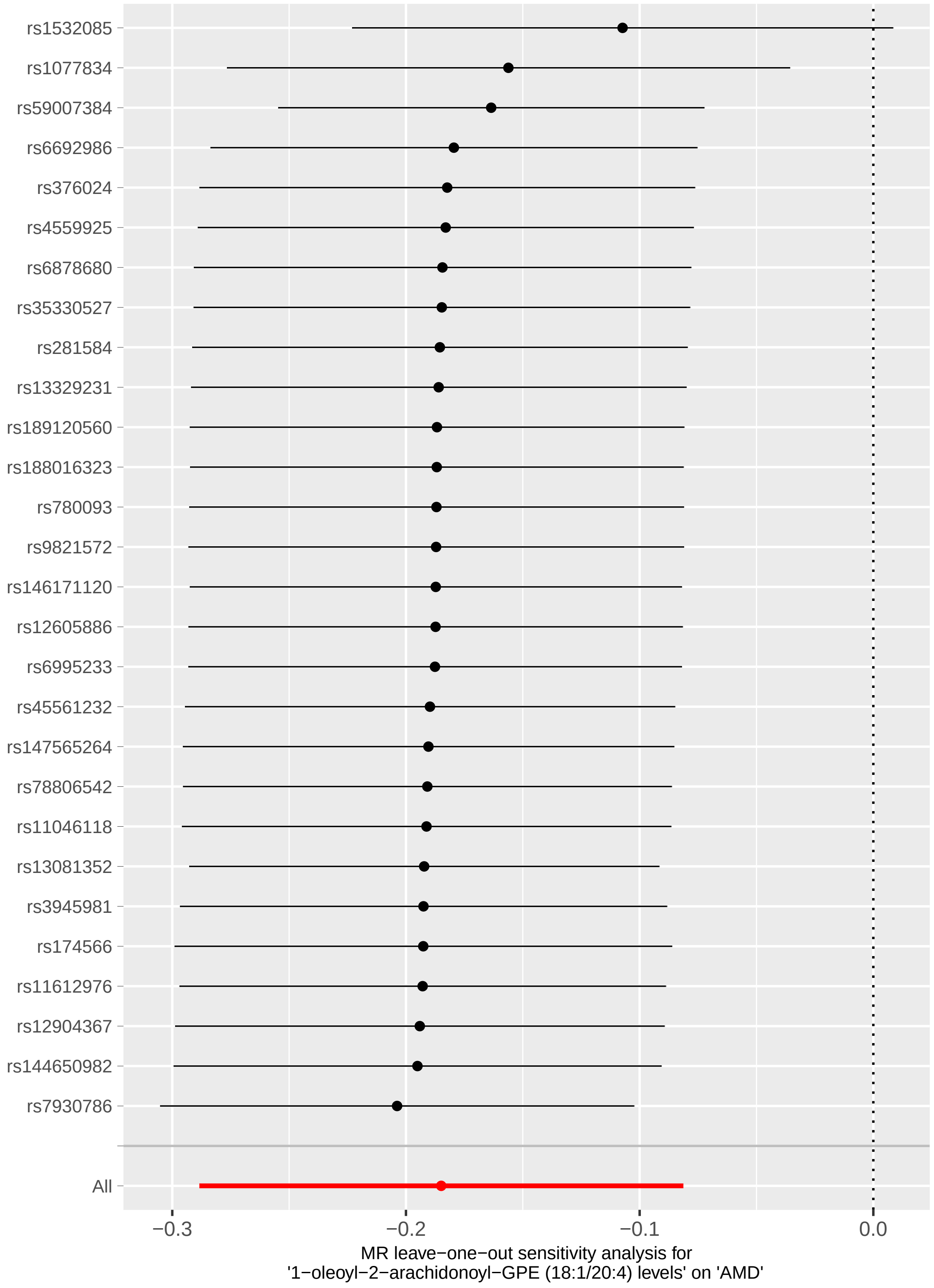

rs1532085
rs1077834
rs59007384
rs6692986
rs376024
rs4559925
rs6878680
rs35330527
rs281584
rs13329231
rs189120560
rs188016323
rs780093
rs9821572
rs146171120
rs12605886
rs6995233
rs45561232
rs147565264
rs78806542
rs11046118
rs13081352
rs3945981
rs174566
rs11612976
rs12904367
rs144650982
rs7930786
All
−0.3
−0.2
−0.1
0.0
MR leave−one−out sensitivity analysis for
'1−oleoyl−2−arachidonoyl−GPE (18:1/20:4) levels' on 'AMD'

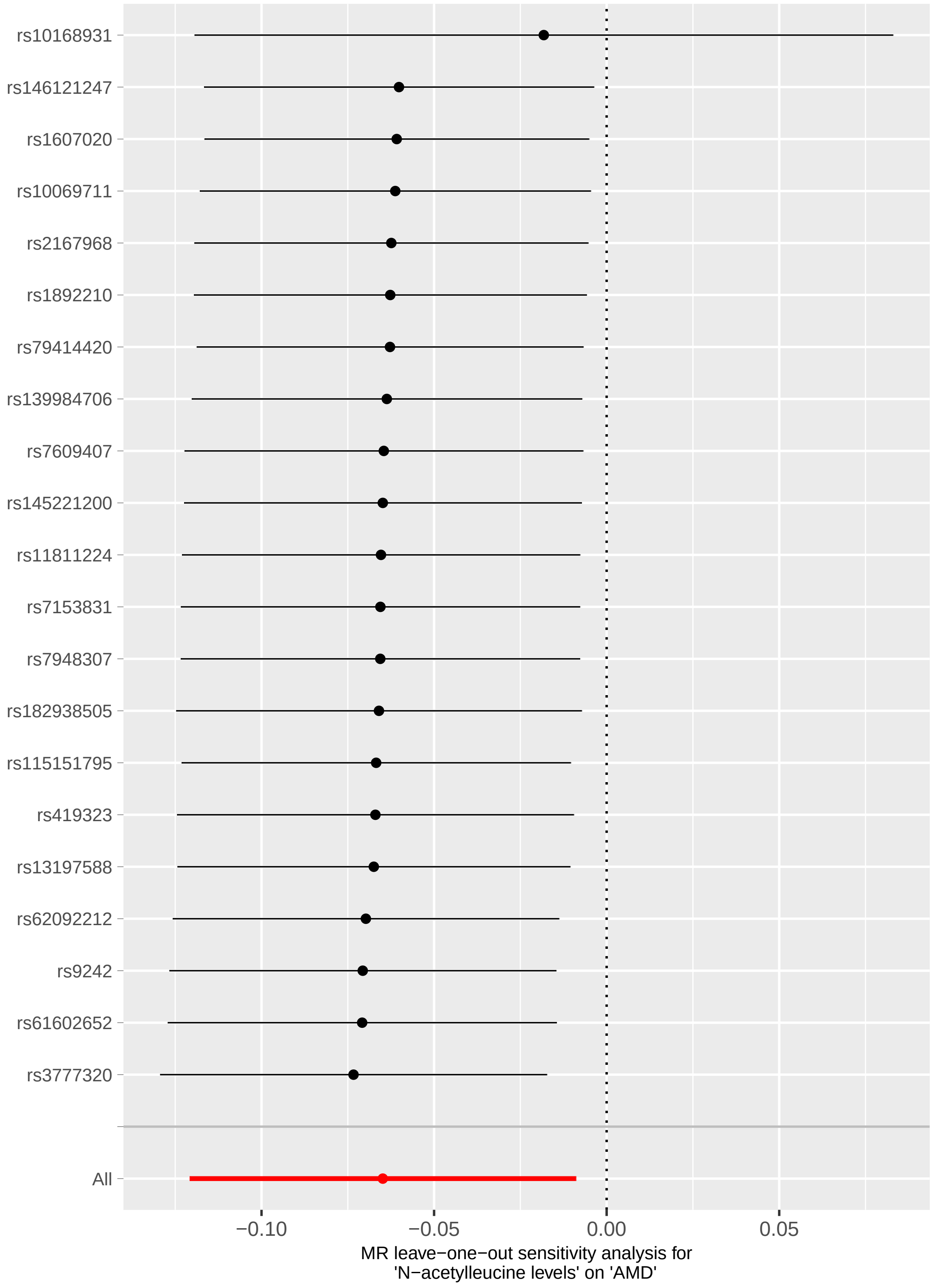

rs10168931
rs146121247
rs1607020
rs10069711
rs2167968
rs1892210
rs79414420
rs139984706
rs7609407
rs145221200
rs11811224
rs7153831
rs7948307
rs182938505
rs115151795
rs419323
rs13197588
rs62092212
rs9242
rs61602652
rs3777320
All
−0.10
−0.05
0.00
0.05
MR leave−one−out sensitivity analysis for
'N−acetylleucine levels' on 'AMD'

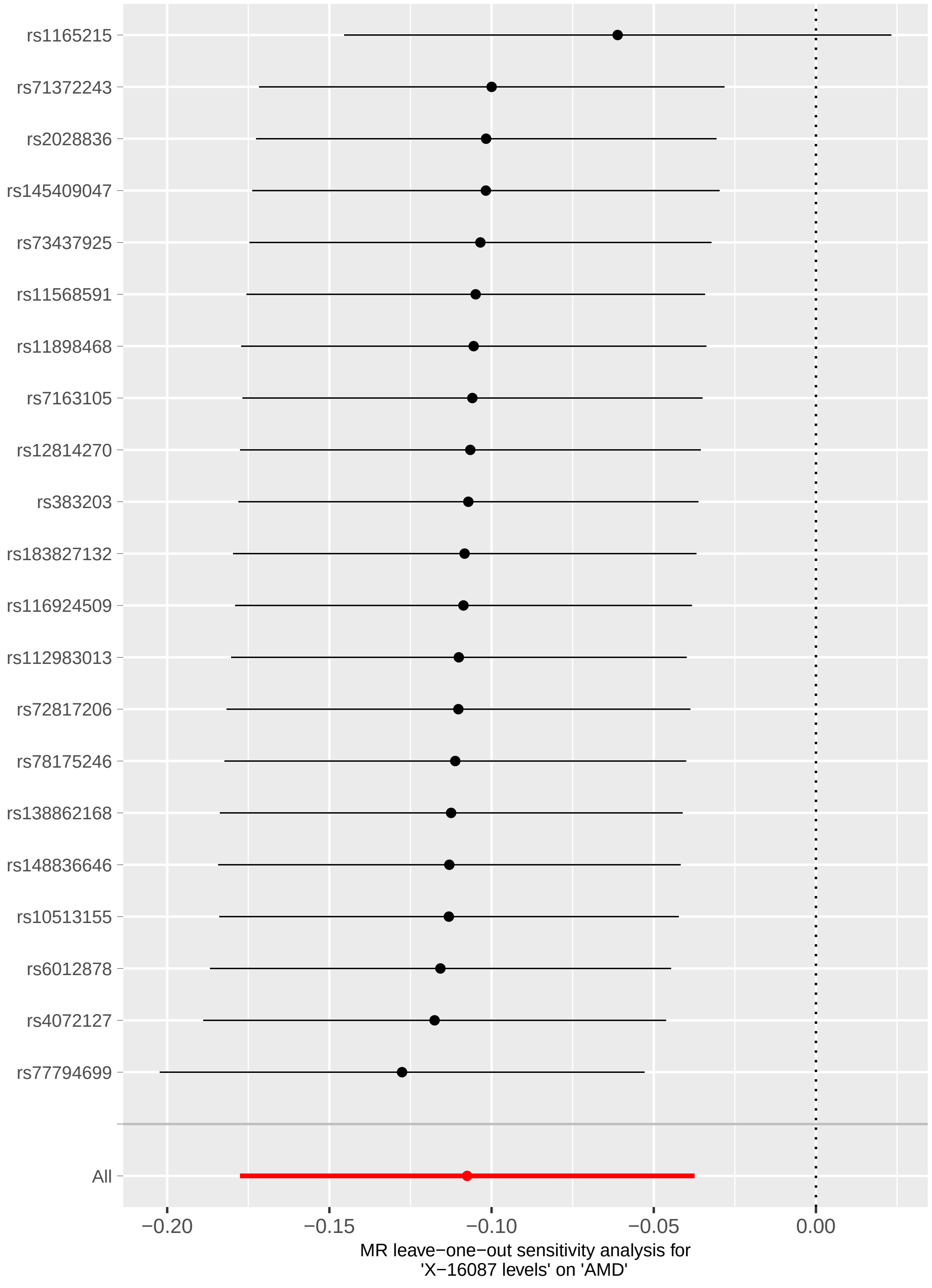

rs1165215
rs71372243
rs2028836
rs145409047
rs73437925
rs11568591
rs11898468
rs7163105
rs12814270
rs383203
rs183827132
rs116924509
rs112983013
rs72817206
rs78175246
rs138862168
rs148836646
rs10513155
rs6012878
rs4072127
rs77794699
All
−0.20
−0.15
−0.10
−0.05
0.00
MR leave−one−out sensitivity analysis for
'X−16087 levels' on 'AMD'

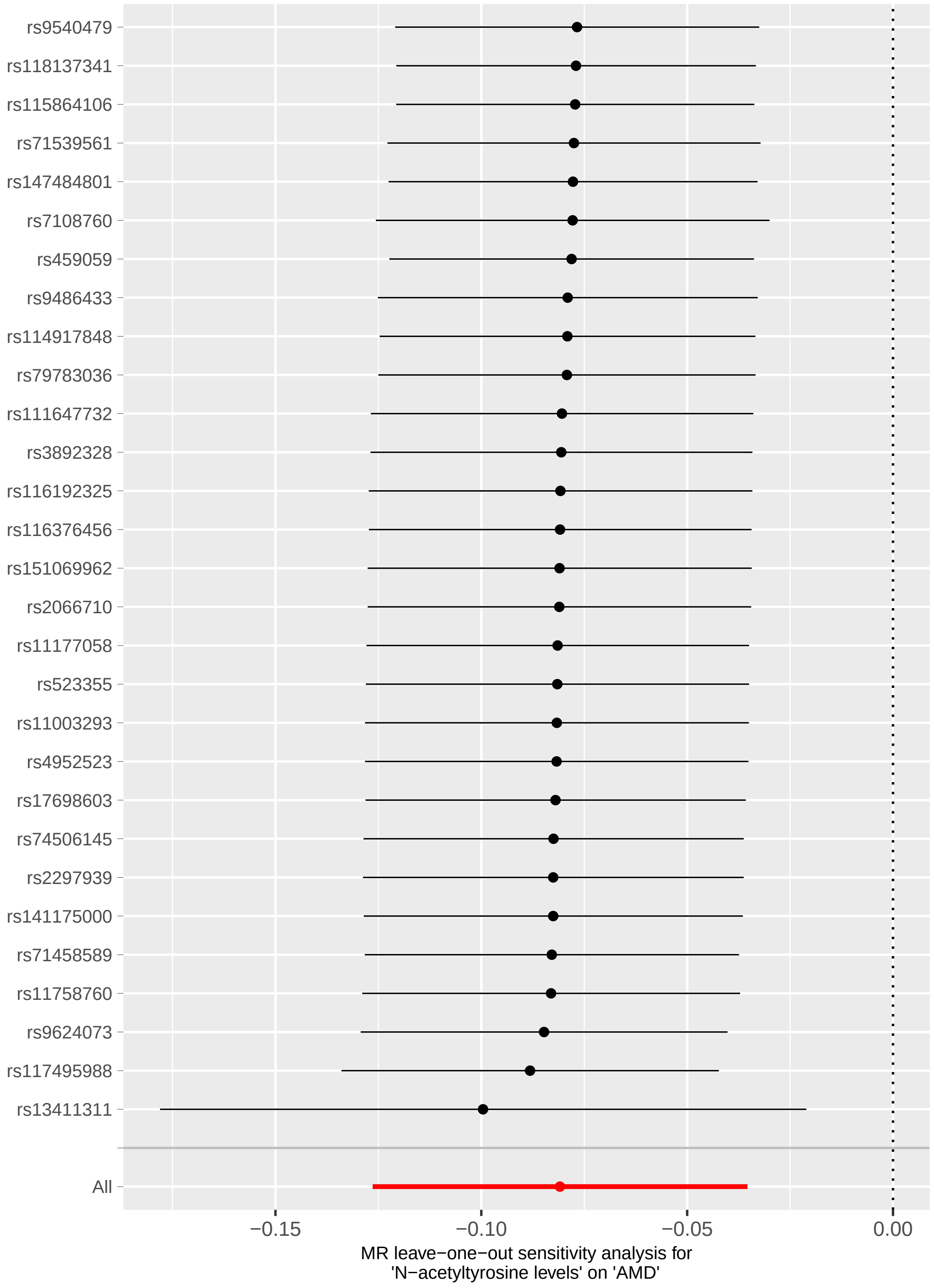

rs9540479
rs118137341
rs115864106
rs71539561
rs147484801
rs7108760
rs459059
rs9486433
rs114917848
rs79783036
rs111647732
rs3892328
rs116192325
rs116376456
rs151069962
rs2066710
rs11177058
rs523355
rs11003293
rs4952523
rs17698603
rs74506145
rs2297939
rs141175000
rs71458589
rs11758760
rs9624073
rs117495988
rs13411311
All
−0.15
−0.10
−0.05
0.00
MR leave−one−out sensitivity analysis for
'N−acetyltyrosine levels' on 'AMD'

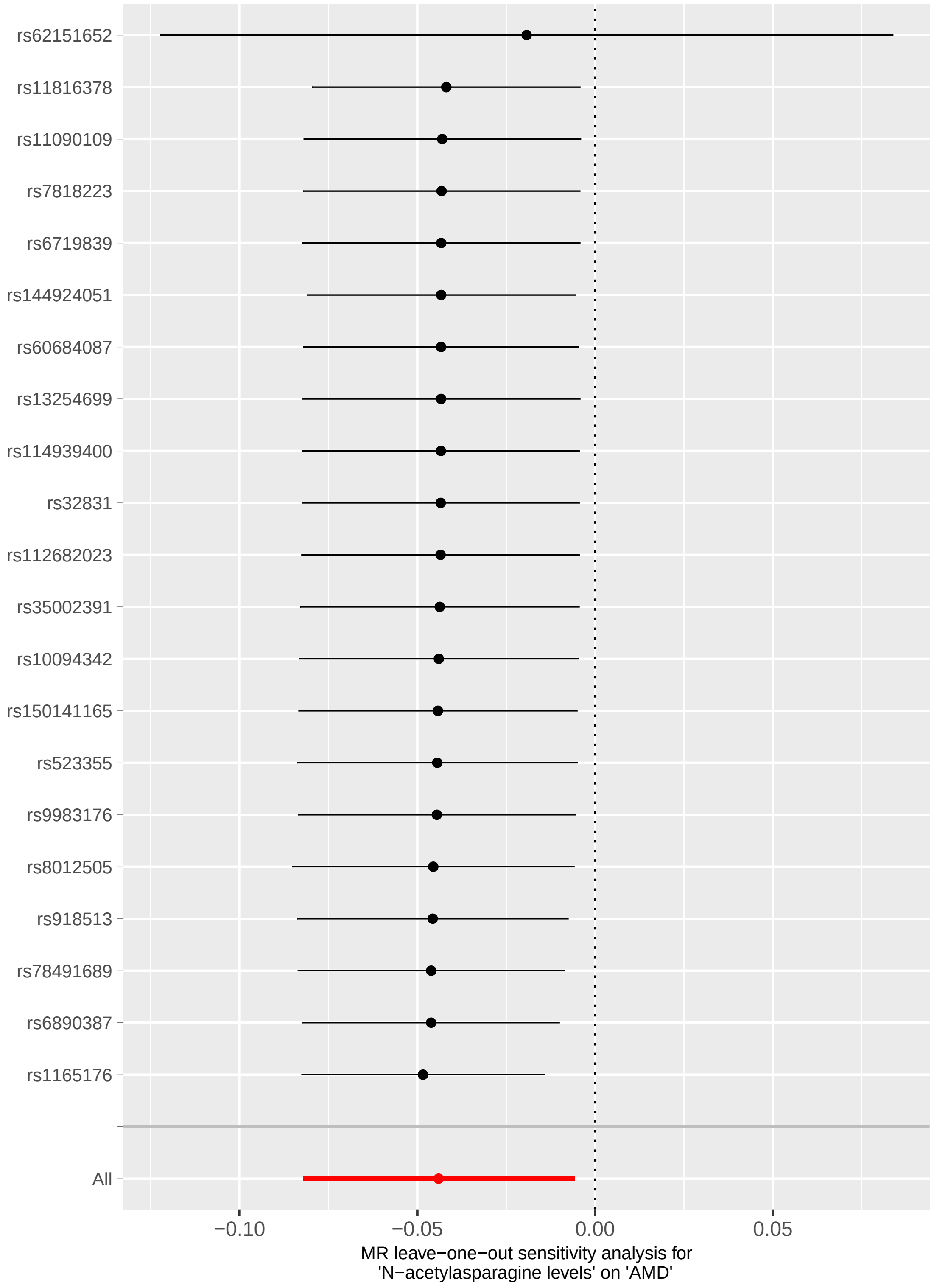
rs62151652
rs11816378
rs11090109
rs7818223
rs6719839
rs144924051
rs60684087
rs13254699
rs114939400
rs32831
rs112682023
rs35002391
rs10094342
rs150141165
rs523355
rs9983176
rs8012505
rs918513
rs78491689
rs6890387
rs1165176
All
−0.10
−0.05
0.00
0.05
MR leave−one−out sensitivity analysis for
'N−acetylasparagine levels' on 'AMD'

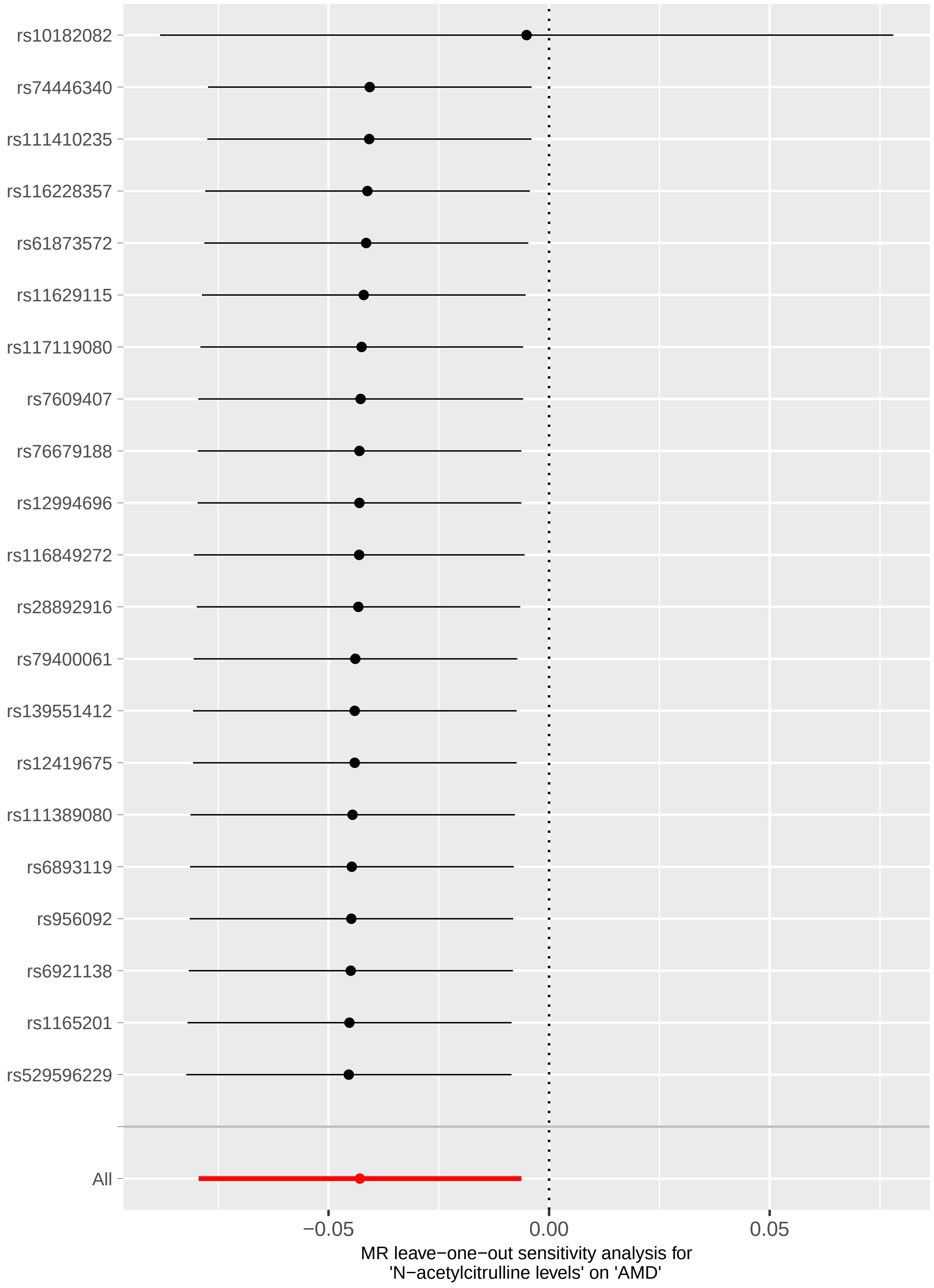

rs10182082
rs74446340
rs111410235
rs116228357
rs61873572
rs11629115
rs117119080
rs7609407
rs76679188
rs12994696
rs116849272
rs28892916
rs79400061
rs139551412
rs12419675
rs111389080
rs6893119
rs956092
rs6921138
rs1165201
rs529596229
All
−0.05
0.00
0.05
MR leave−one−out sensitivity analysis for
'N−acetylcitrulline levels' on 'AMD'

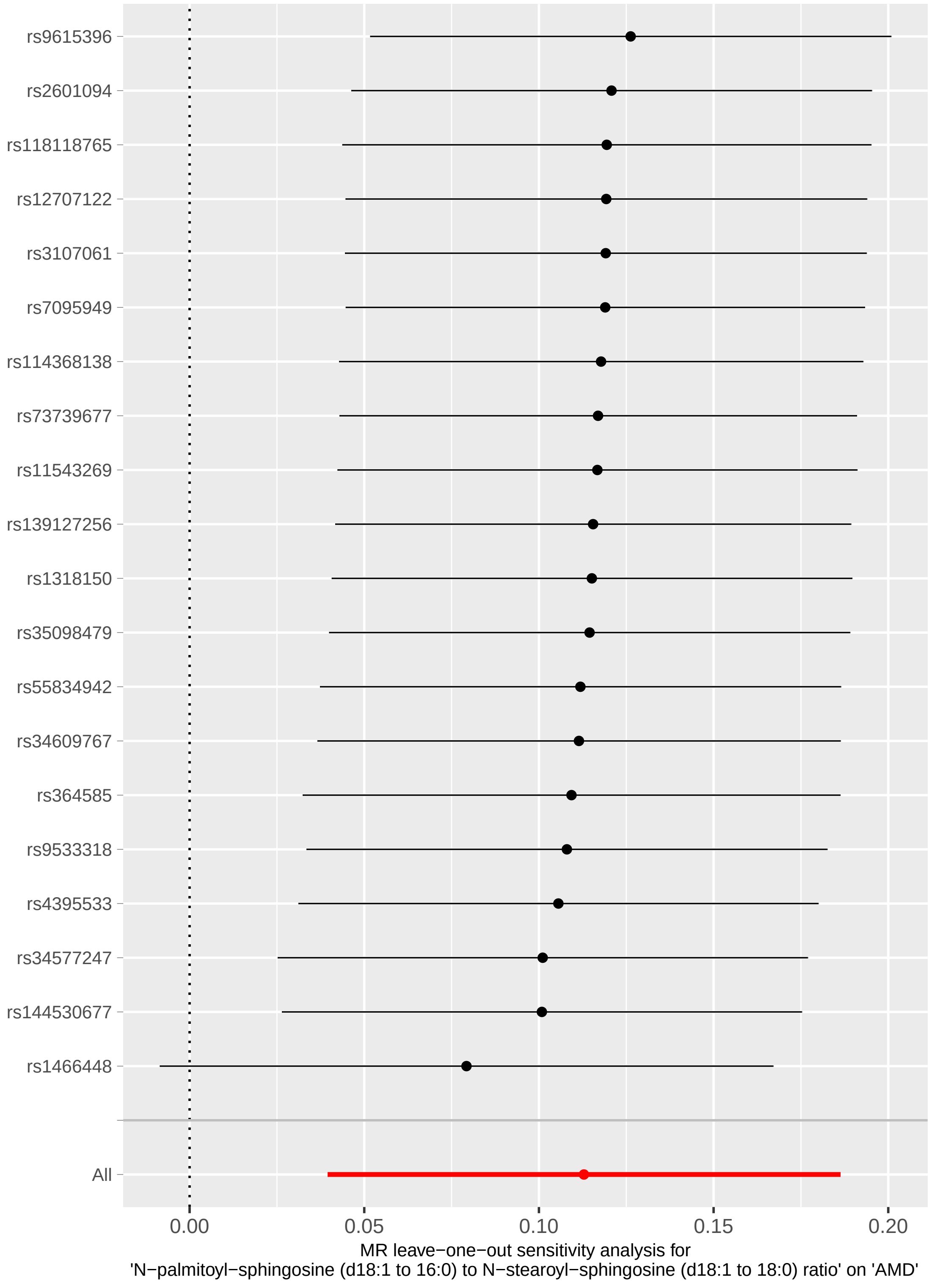
rs9615396
rs2601094
rs118118765
rs12707122
rs3107061
rs7095949
rs114368138
rs73739677
rs11543269
rs139127256
rs1318150
rs35098479
rs55834942
rs34609767
rs364585
rs9533318
rs4395533
rs34577247
rs144530677
rs1466448
All
0.00
0.05
0.10
0.15
0.20
MR leave−one−out sensitivity analysis for
'N−palmitoyl−sphingosine (d18:1 to 16:0) to N−stearoyl−sphingosine (d18:1 to 18:0) ratio' on 'AMD'

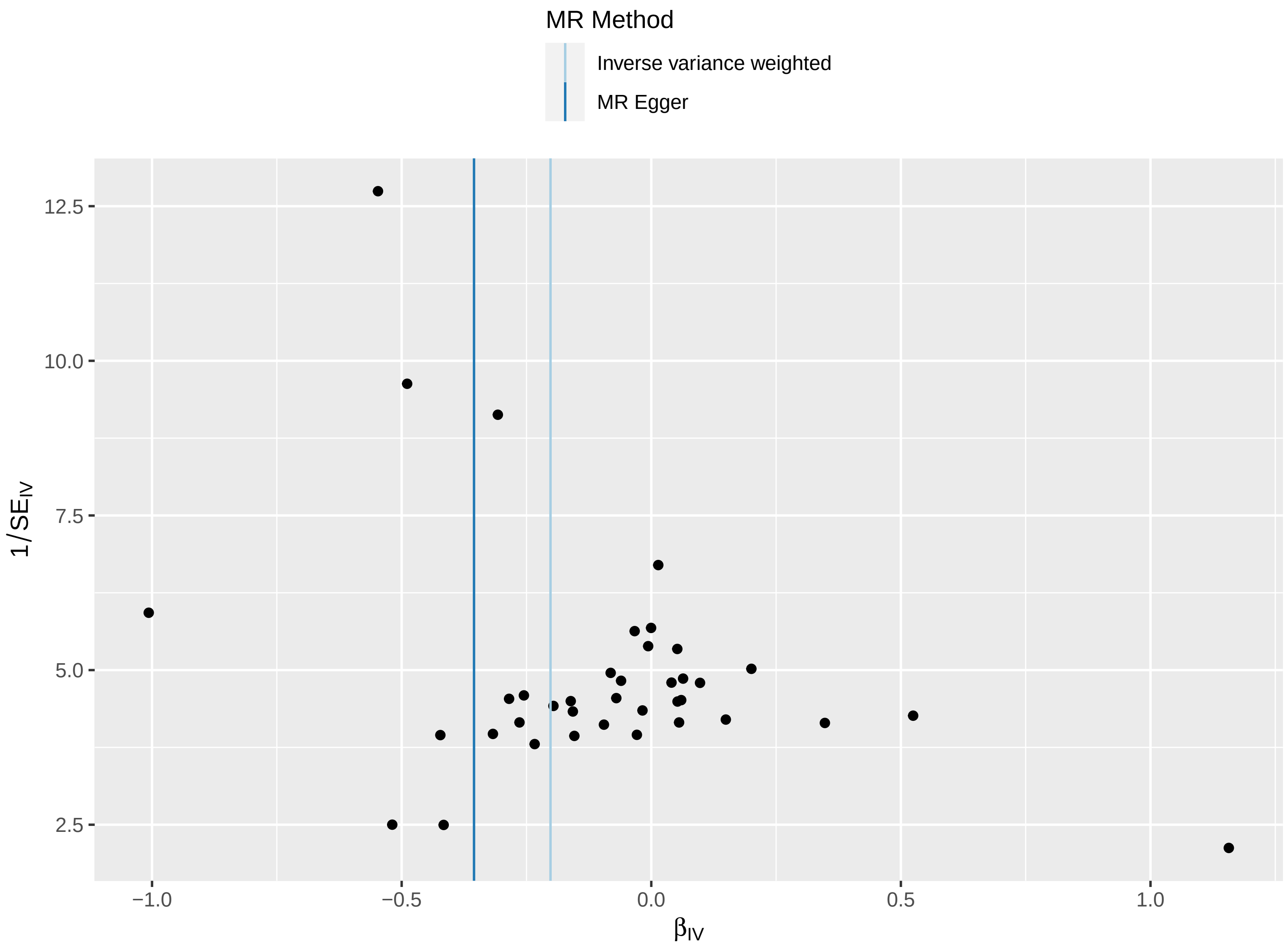
MR Method
Inverse variance weighted
MR Egger
12.5
10.0
7.5
5.0
2.5
$1/SE_{IV}$
−1.0
−0.5
0.0
0.5
1.0
β_{IV}

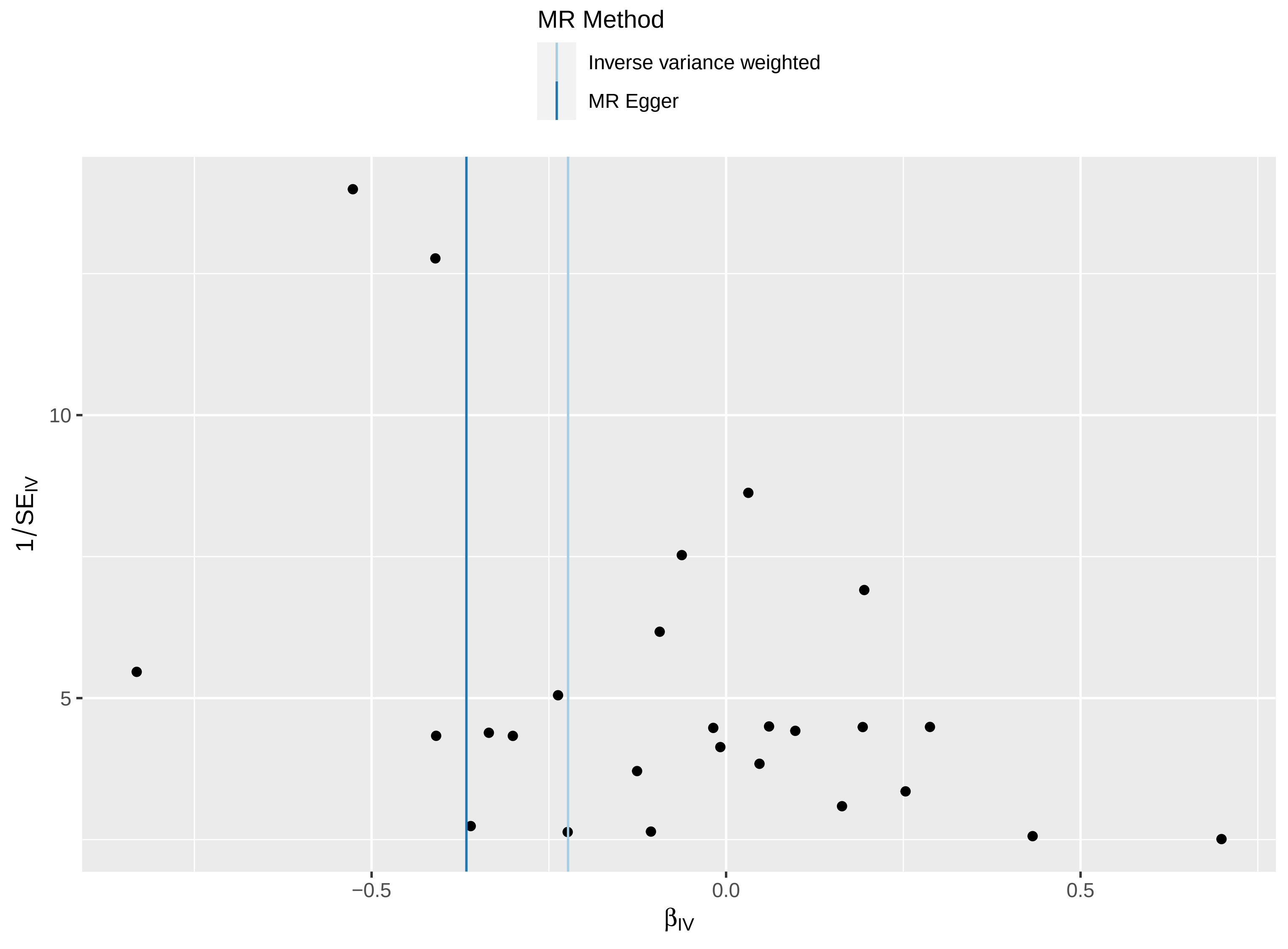
MR Method
Inverse variance weighted
MR Egger
1/SE_IV
10
5
−0.5
0.0
0.5
β_IV

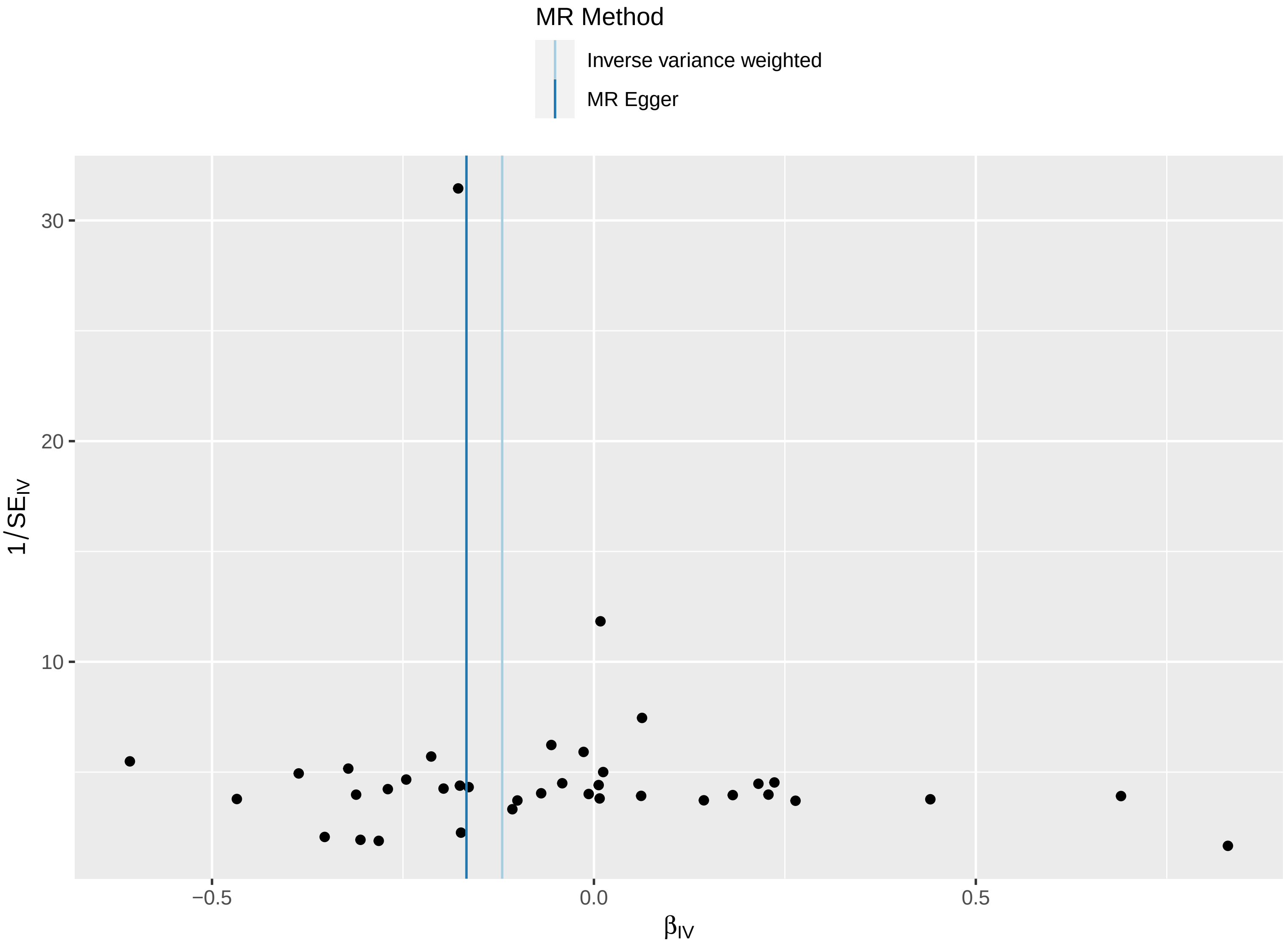

MR Method
Inverse variance weighted
MR Egger
30
20
10
1/SE$_{IV}$
−0.5
0.0
0.5
β$_{IV}$

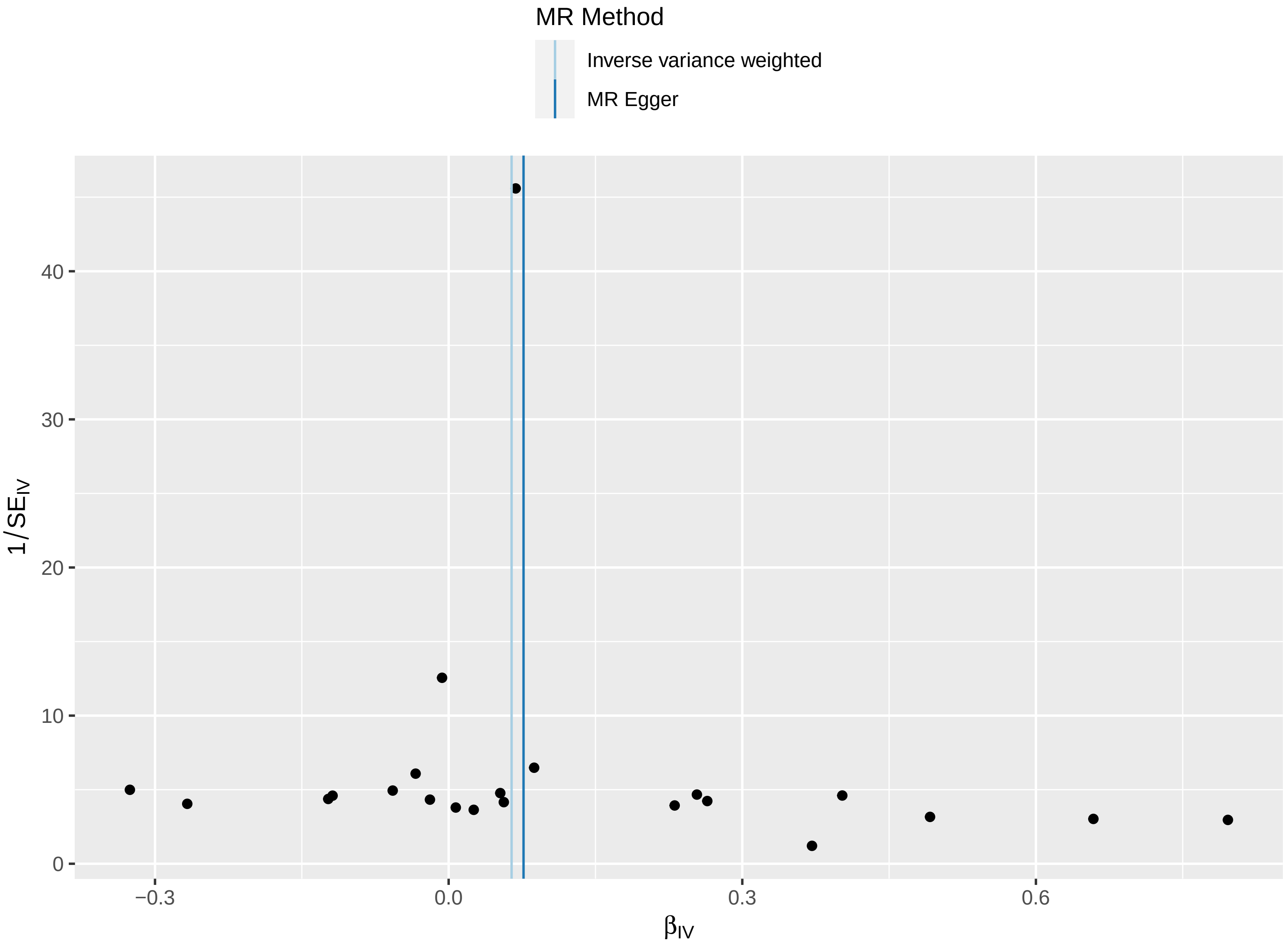
MR Method
Inverse variance weighted
MR Egger
1/SE_IV
40
30
20
10
0
−0.3
0.0
0.3
0.6
β_IV

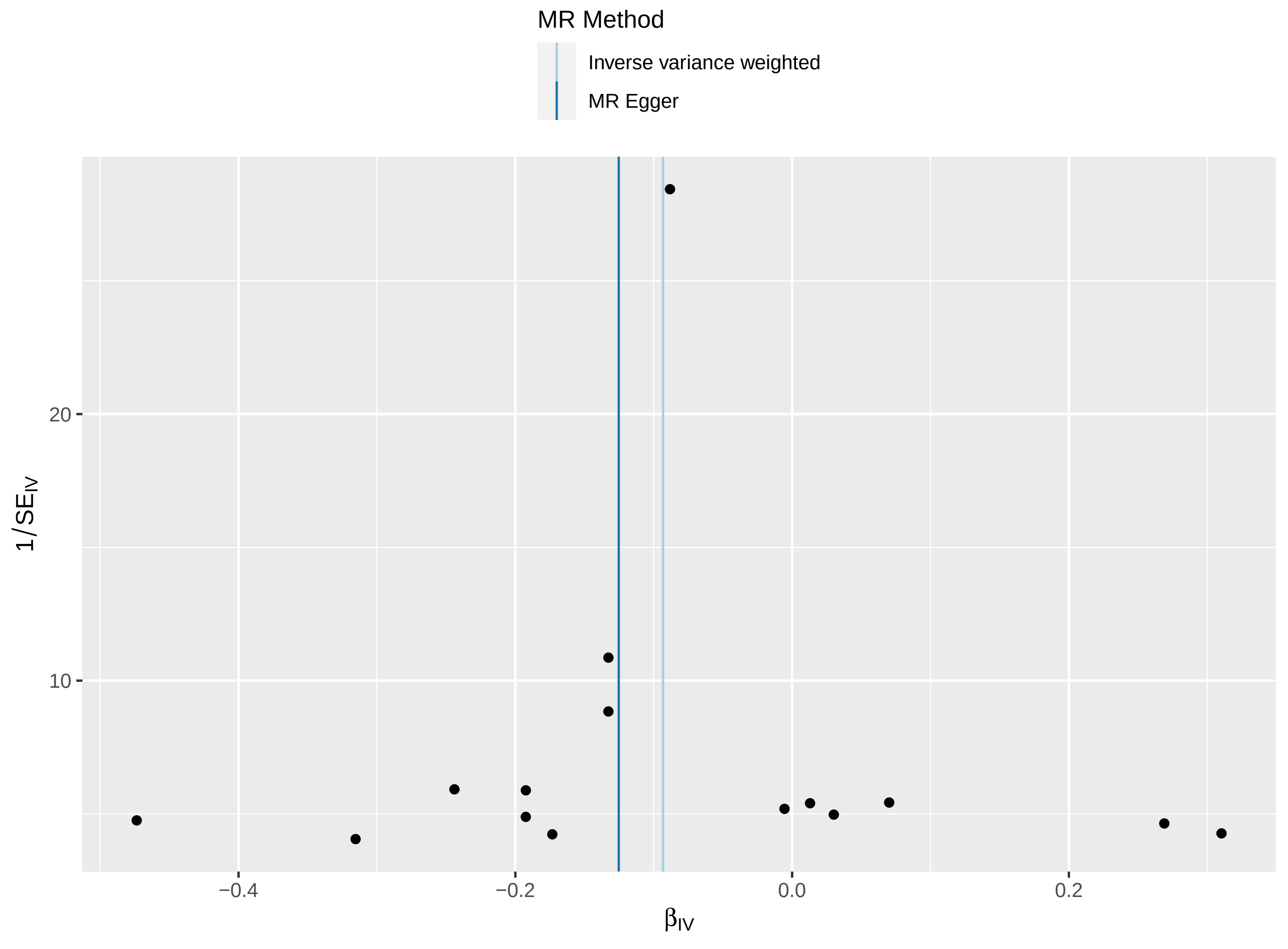

MR Method
Inverse variance weighted
MR Egger
20
10
1/SE$_{IV}$
−0.4
−0.2
0.0
0.2
β$_{IV}$

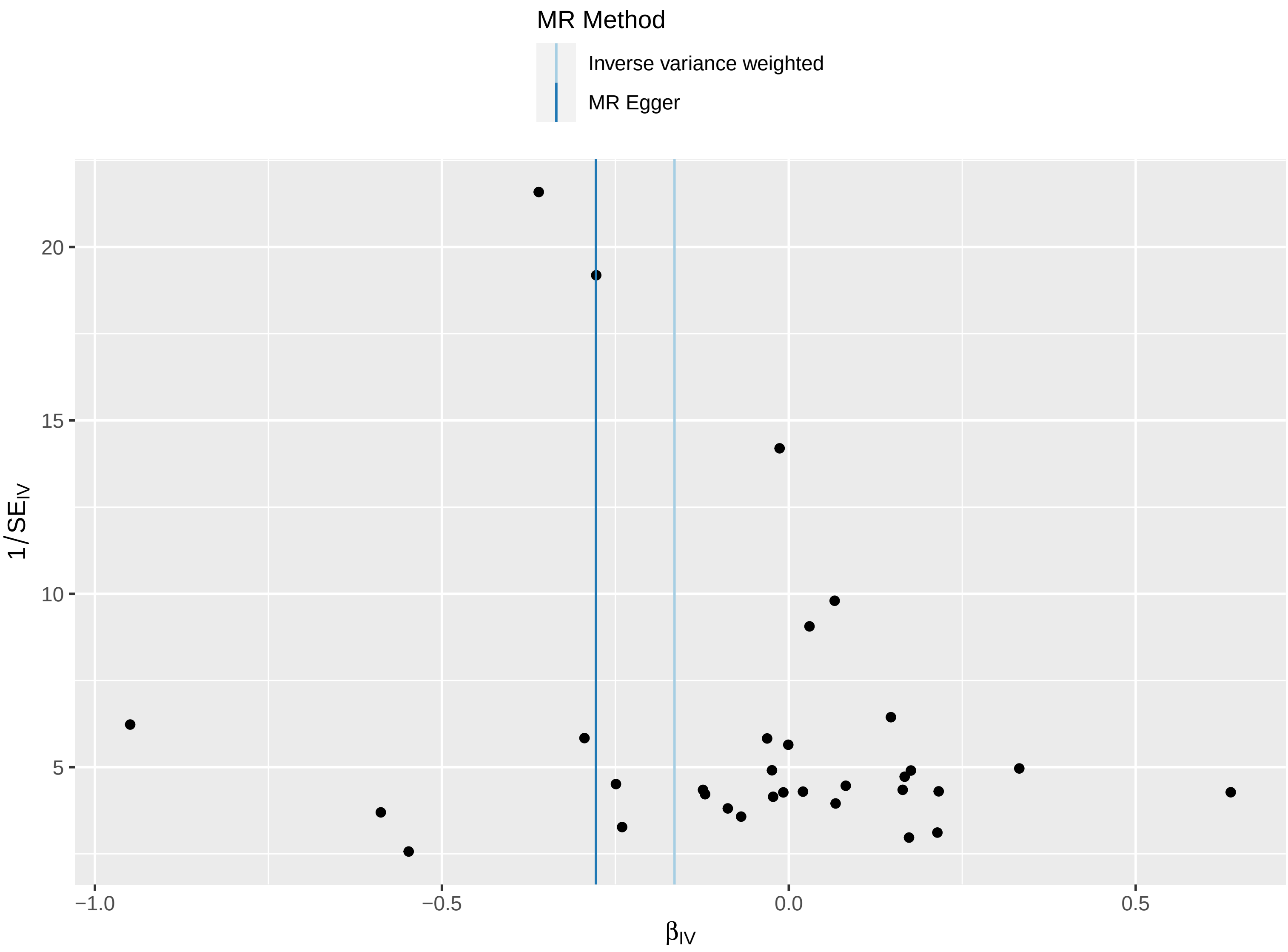
MR Method
Inverse variance weighted
MR Egger
20
15
10
5
1/SE$_{IV}$
−1.0
−0.5
0.0
0.5
β$_{IV}$

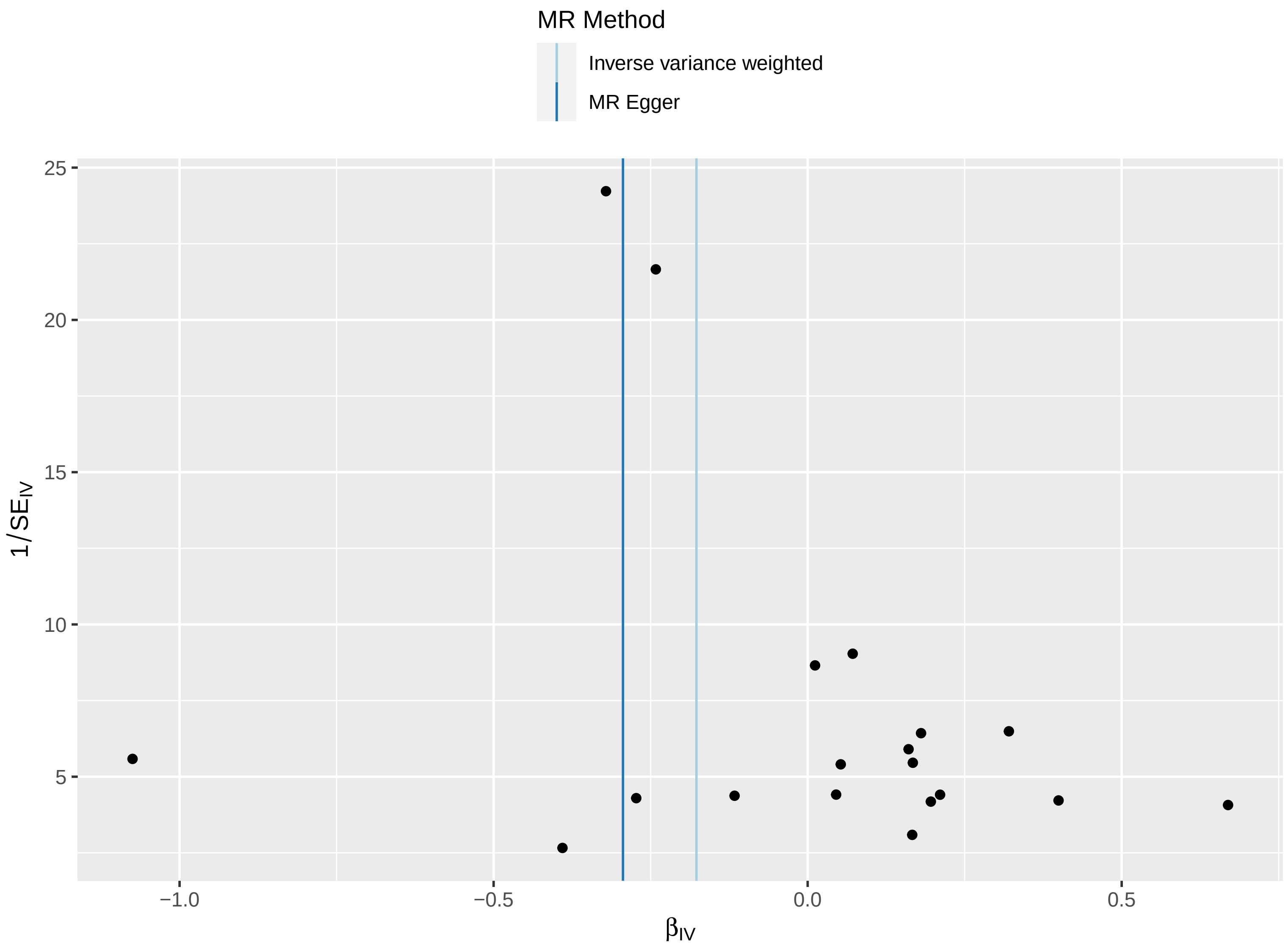
MR Method
Inverse variance weighted
MR Egger
25
20
15
10
5
$1/SE_{IV}$
−1.0
−0.5
0.0
0.5
β_{IV}

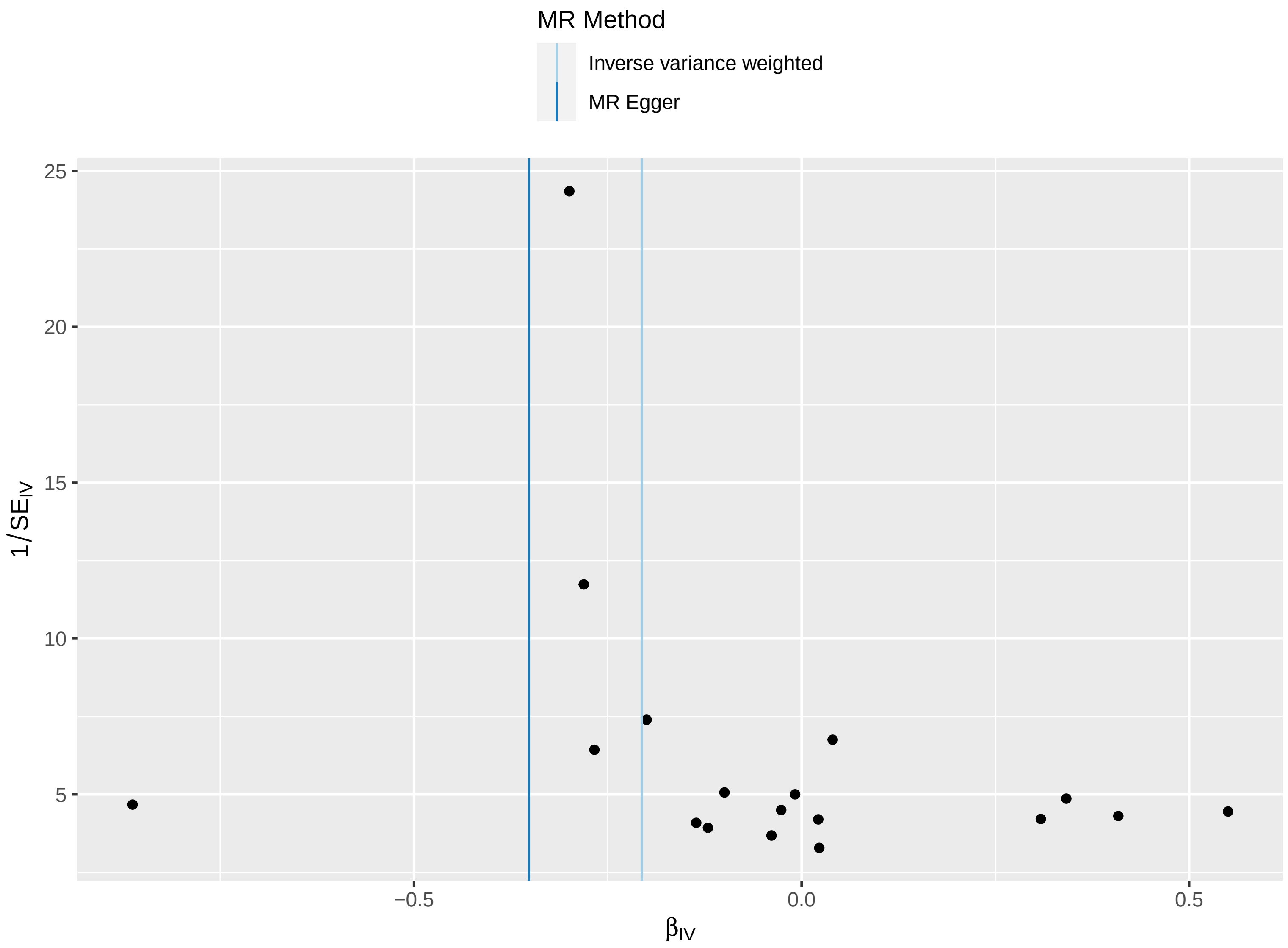
MR Method
Inverse variance weighted
MR Egger
25
20
15
10
5
1/SE$_{IV}$
−0.5
0.0
0.5
β$_{IV}$

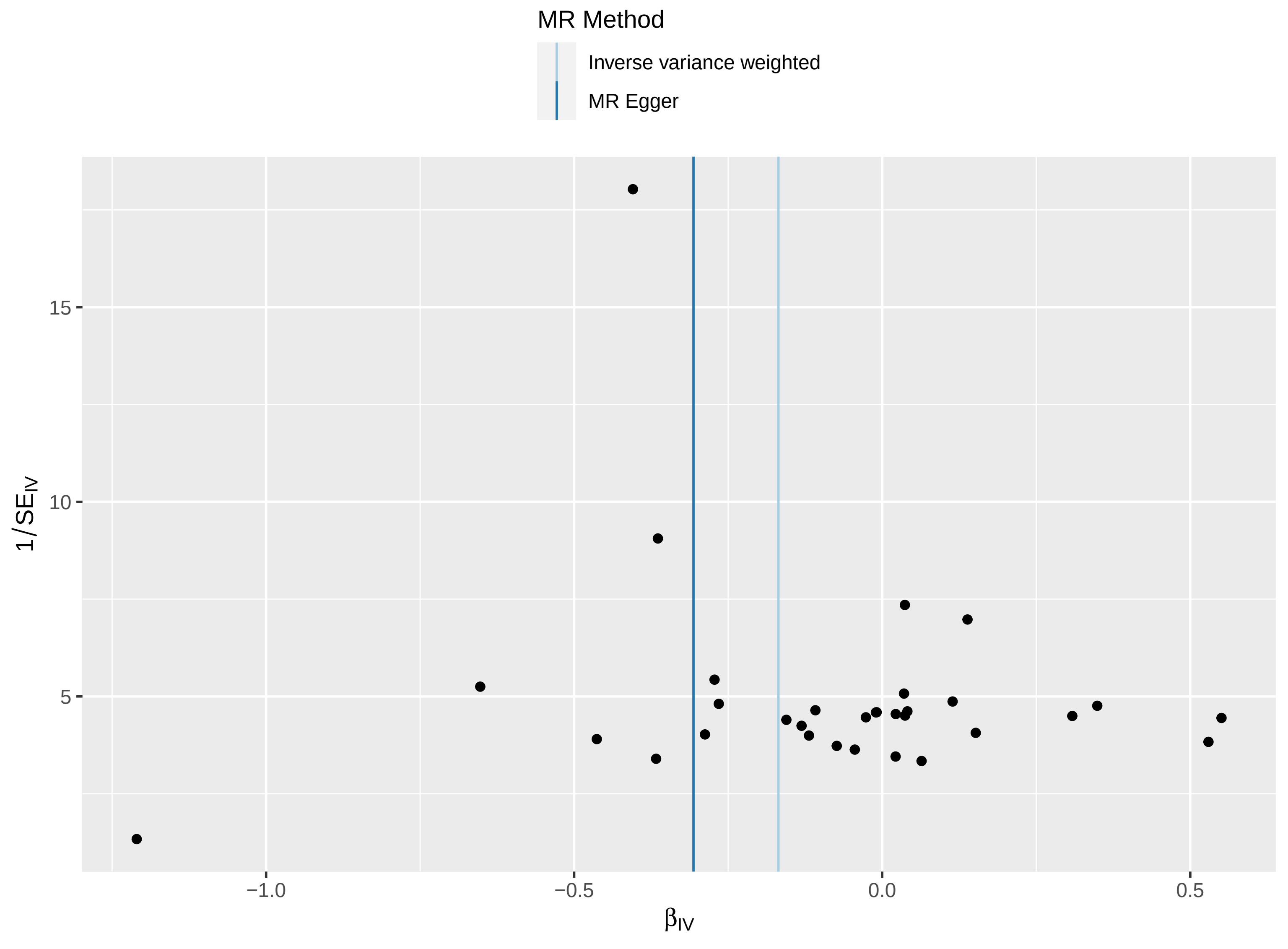

MR Method
Inverse variance weighted
MR Egger
15
10
5
1/SE_IV
−1.0
−0.5
0.0
0.5
β_IV

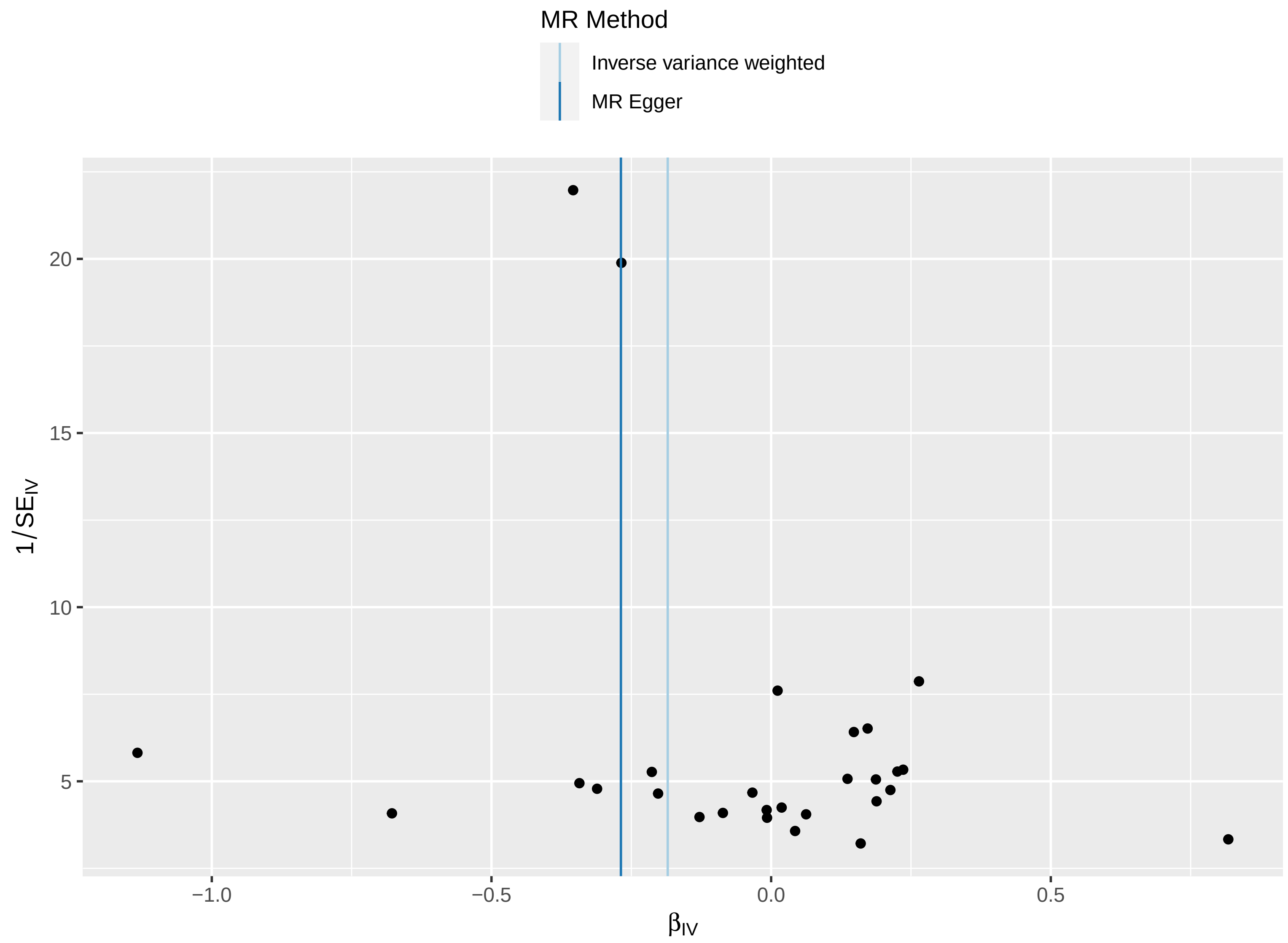
MR Method
Inverse variance weighted
MR Egger
20
15
10
5
1/SE$_{IV}$
−1.0
−0.5
0.0
0.5
β$_{IV}$

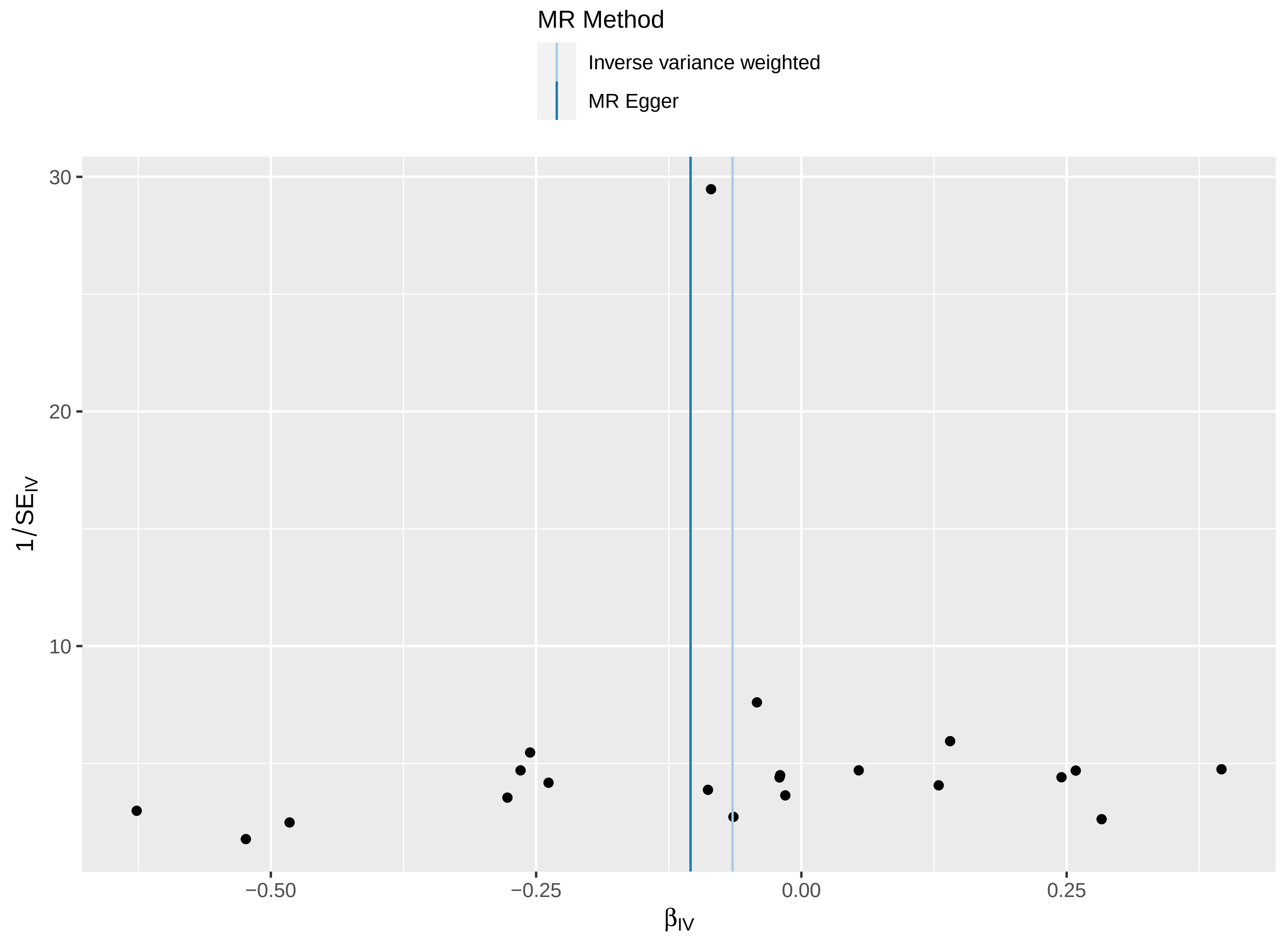

MR Method
Inverse variance weighted
MR Egger
30
20
10
1/SE_IV
−0.50
−0.25
0.00
0.25
β_IV

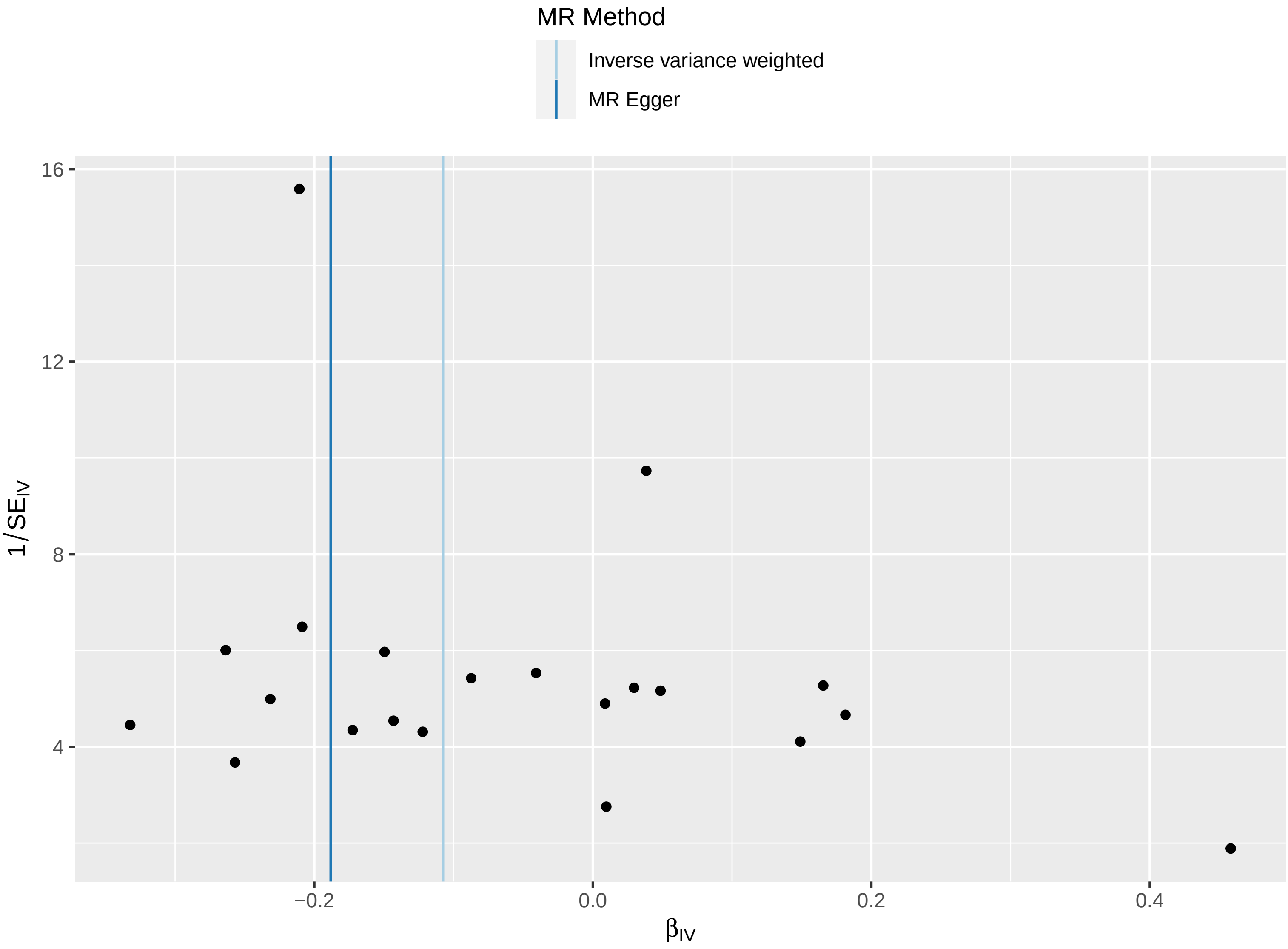
MR Method
Inverse variance weighted
MR Egger
16
12
8
4
$1/SE_{IV}$
−0.2
0.0
0.2
0.4
β_{IV}

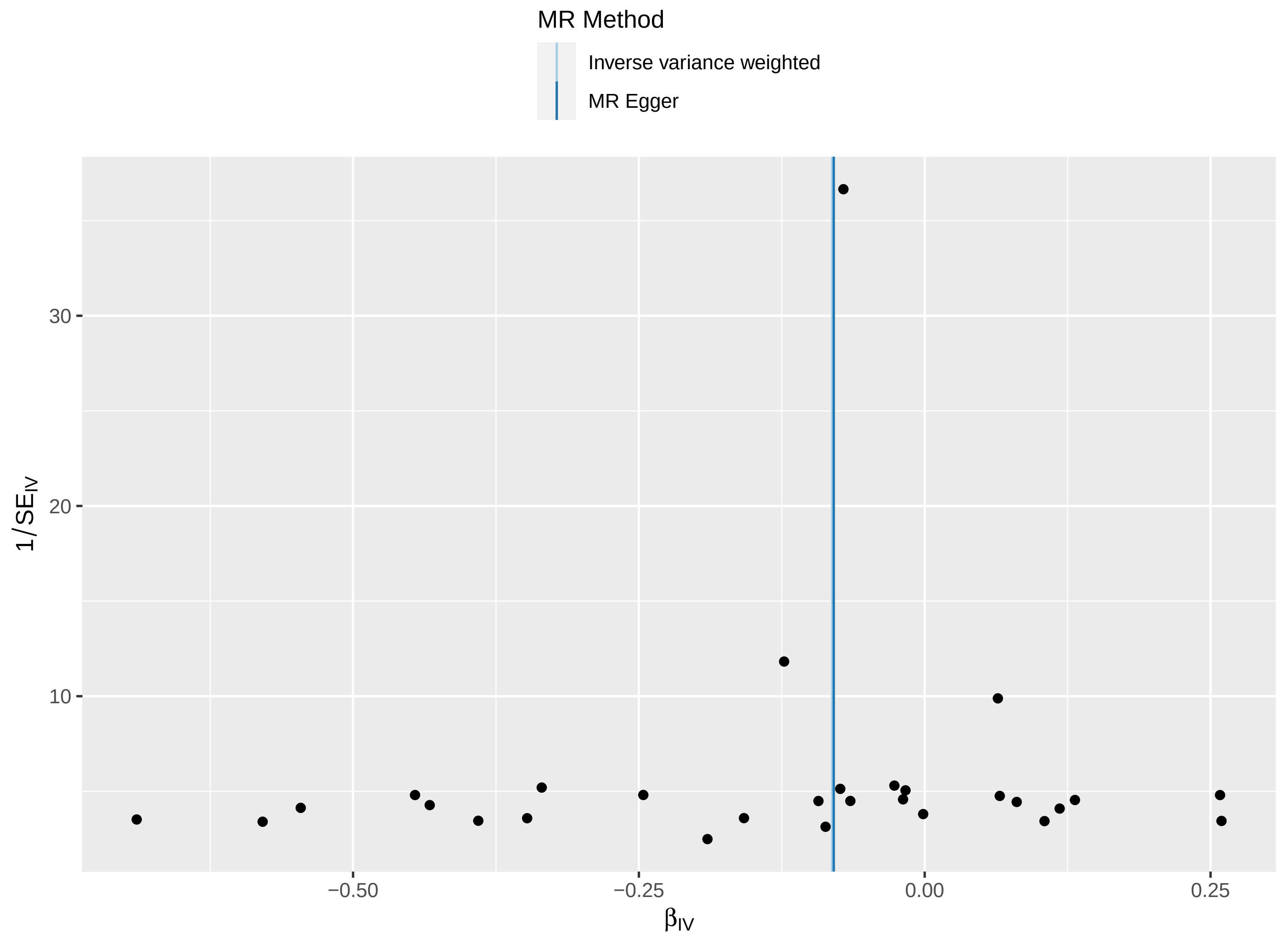

MR Method
Inverse variance weighted
MR Egger
30
20
10
1/SE_IV
−0.50
−0.25
0.00
0.25
β_IV

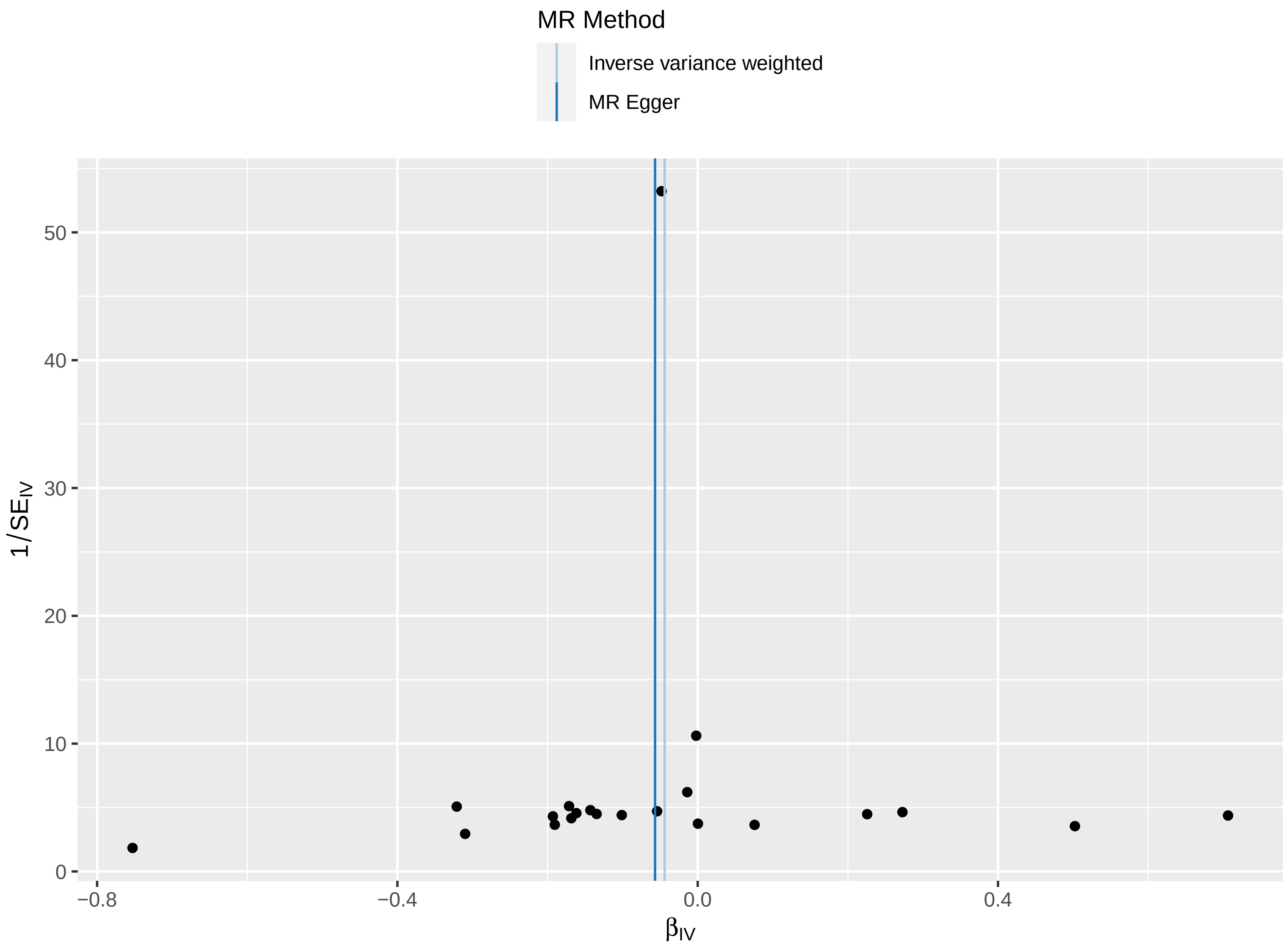

MR Method
Inverse variance weighted
MR Egger
50
40
30
20
10
0
$1/SE_{IV}$
−0.8
−0.4
0.0
0.4
β_{IV}

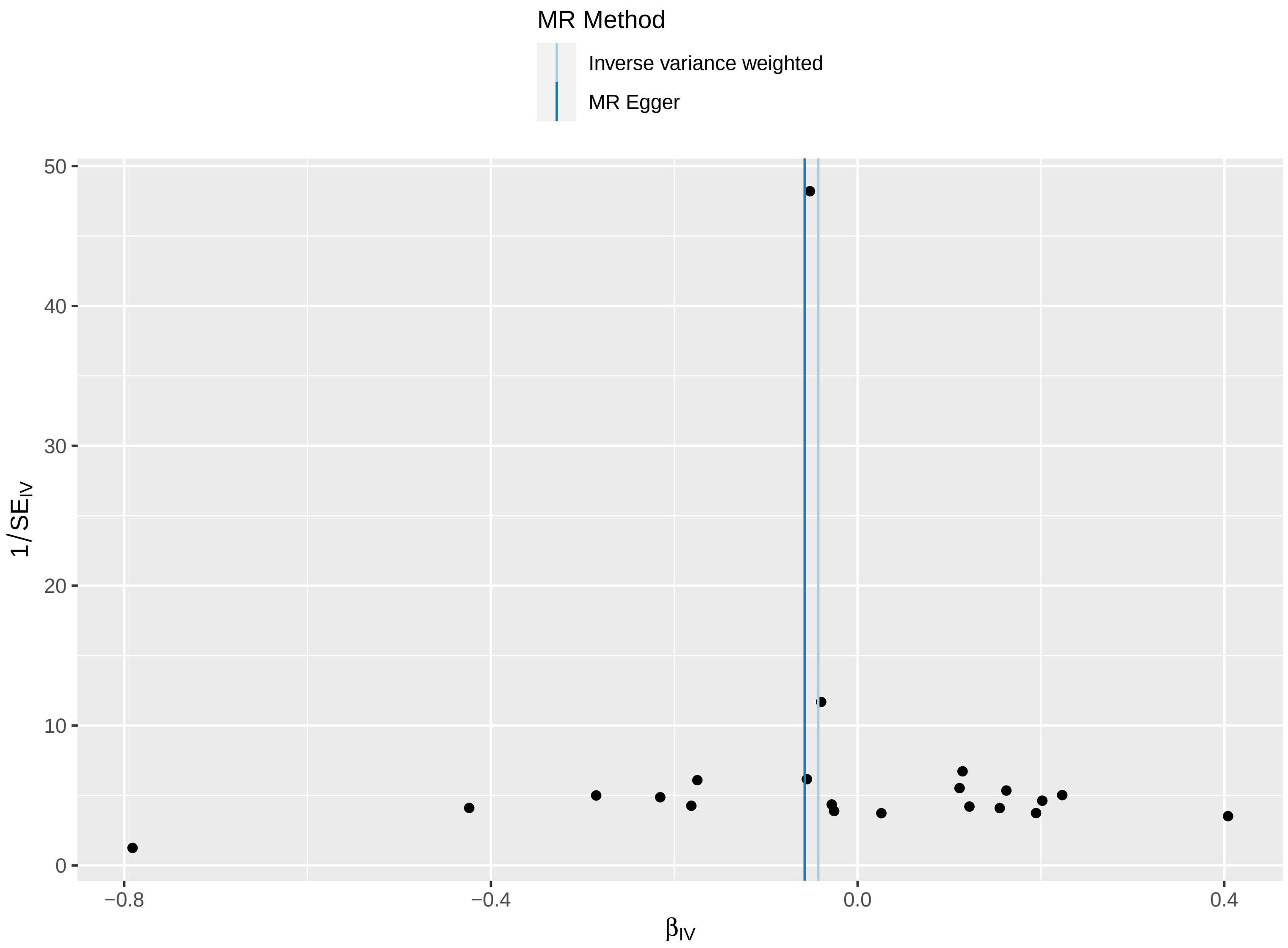
MR Method
Inverse variance weighted
MR Egger
1/SE_{IV}
50
40
30
20
10
0
−0.8
−0.4
0.0
0.4
β_{IV}

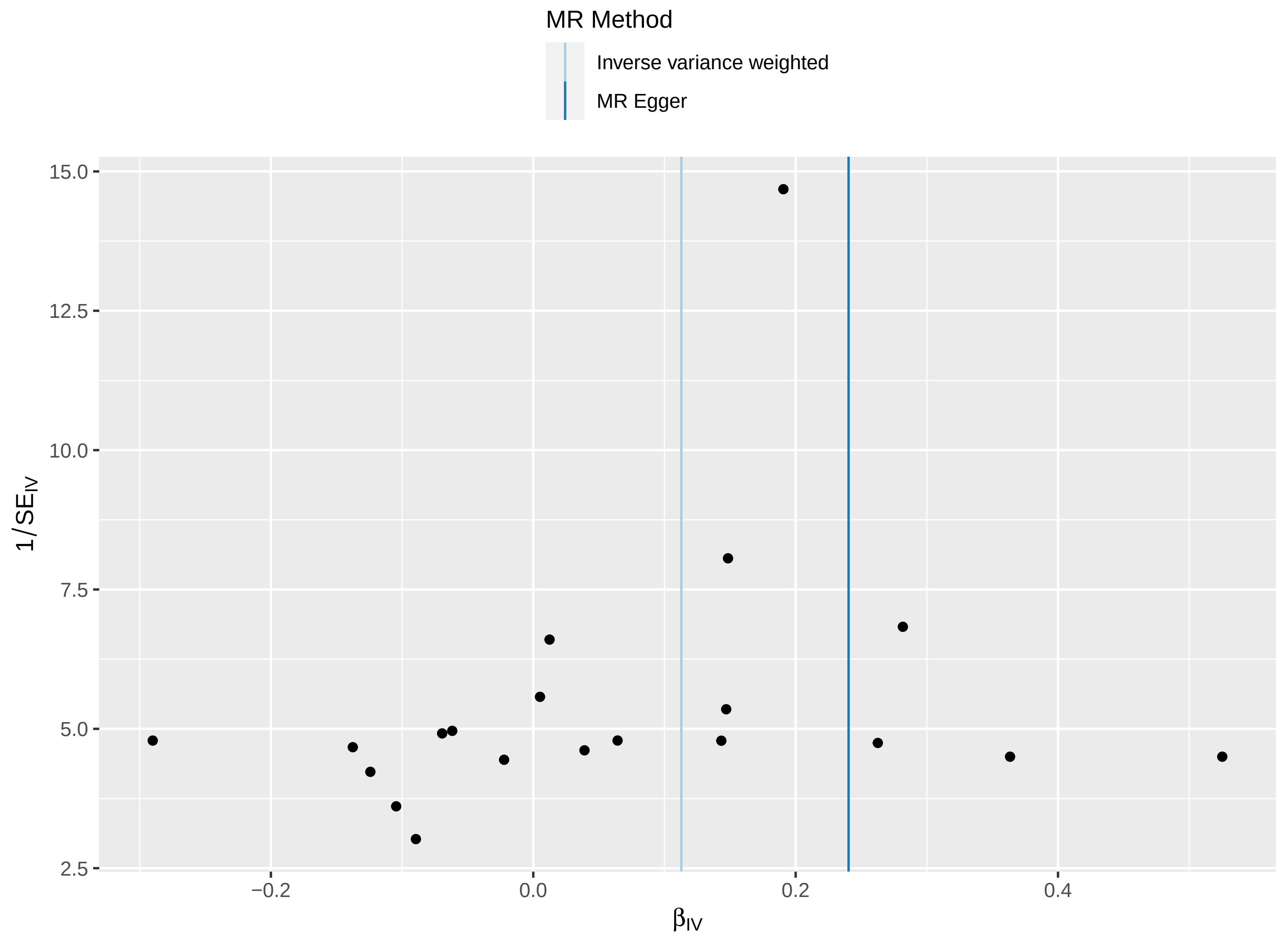
MR Method
Inverse variance weighted
MR Egger
15.0
12.5
10.0
7.5
5.0
2.5
$1/SE_{IV}$
−0.2
0.0
0.2
0.4
β_{IV}

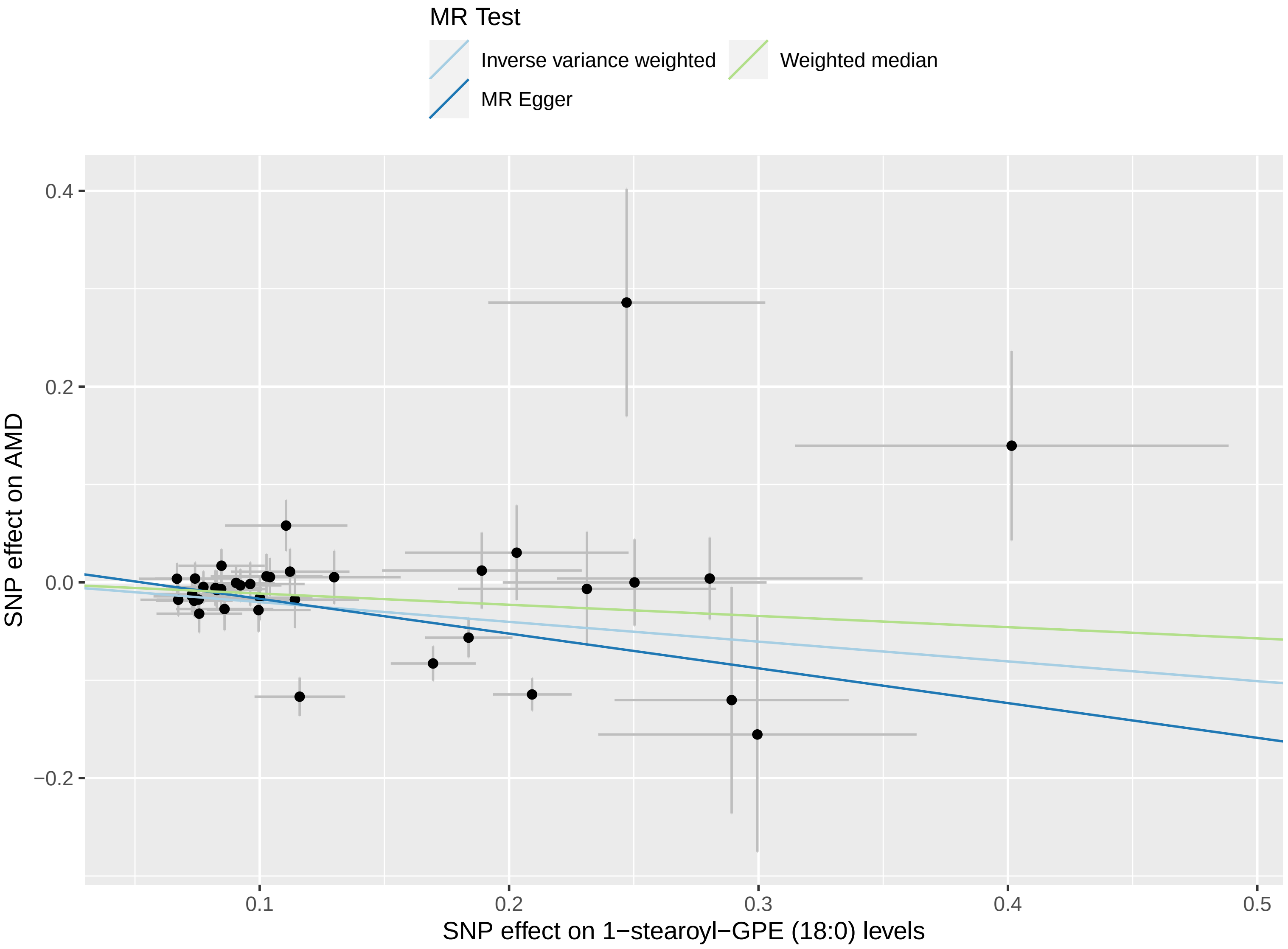
MR Test
Inverse variance weighted
Weighted median
MR Egger
SNP effect on AMD
0.4
0.2
0.0
−0.2
0.1
0.2
0.3
0.4
0.5
SNP effect on 1−stearoyl−GPE (18:0) levels

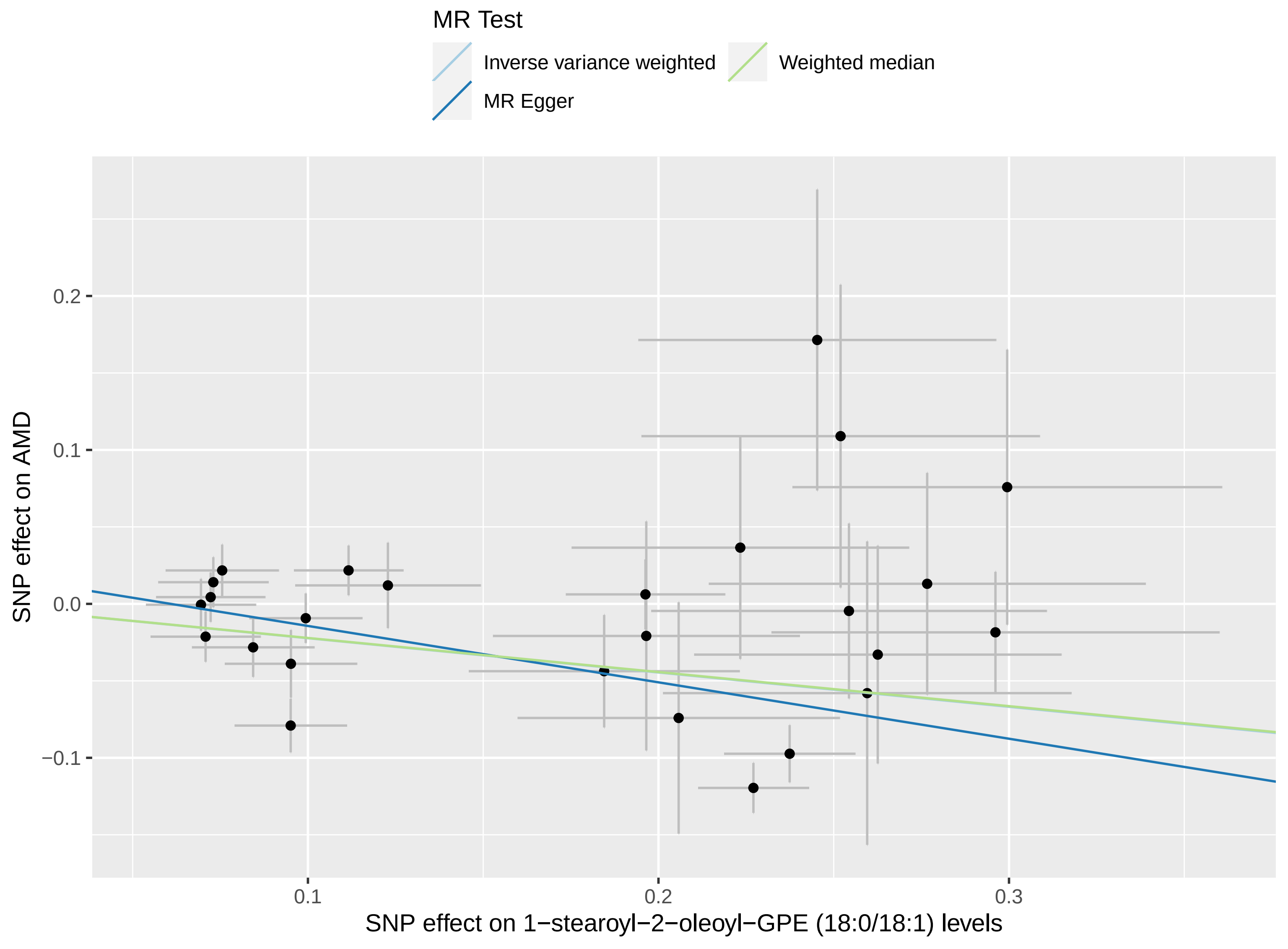

MR Test
Inverse variance weighted
Weighted median
MR Egger
0.2
0.1
0.0
−0.1
SNP effect on AMD
0.1
0.2
0.3
SNP effect on 1−stearoyl−2−oleoyl−GPE (18:0/18:1) levels

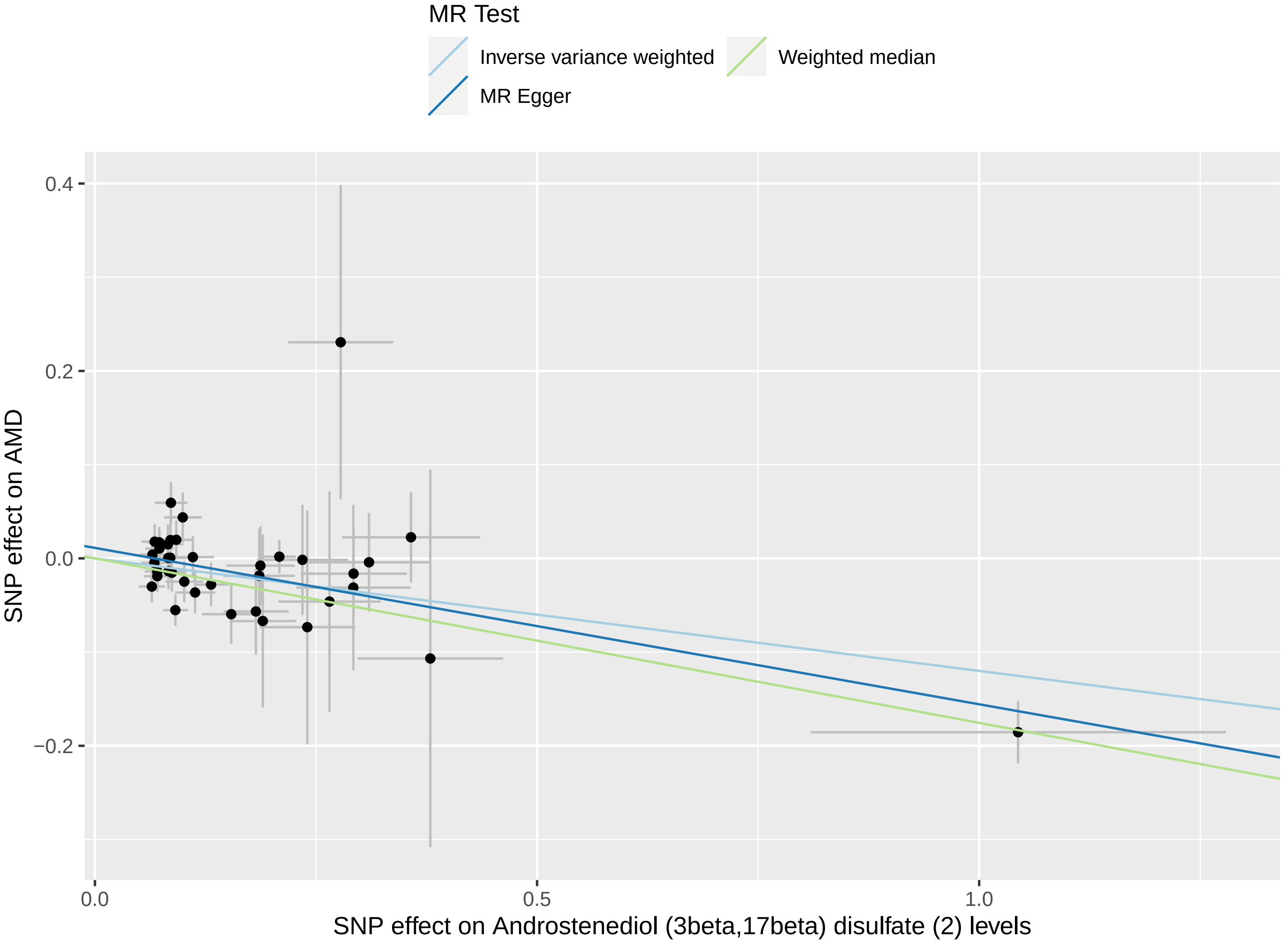
MR Test
Inverse variance weighted
MR Egger
Weighted median
0.4
0.2
0.0
−0.2
SNP effect on AMD
0.0
0.5
1.0
SNP effect on Androstenediol (3beta,17beta) disulfate (2) levels

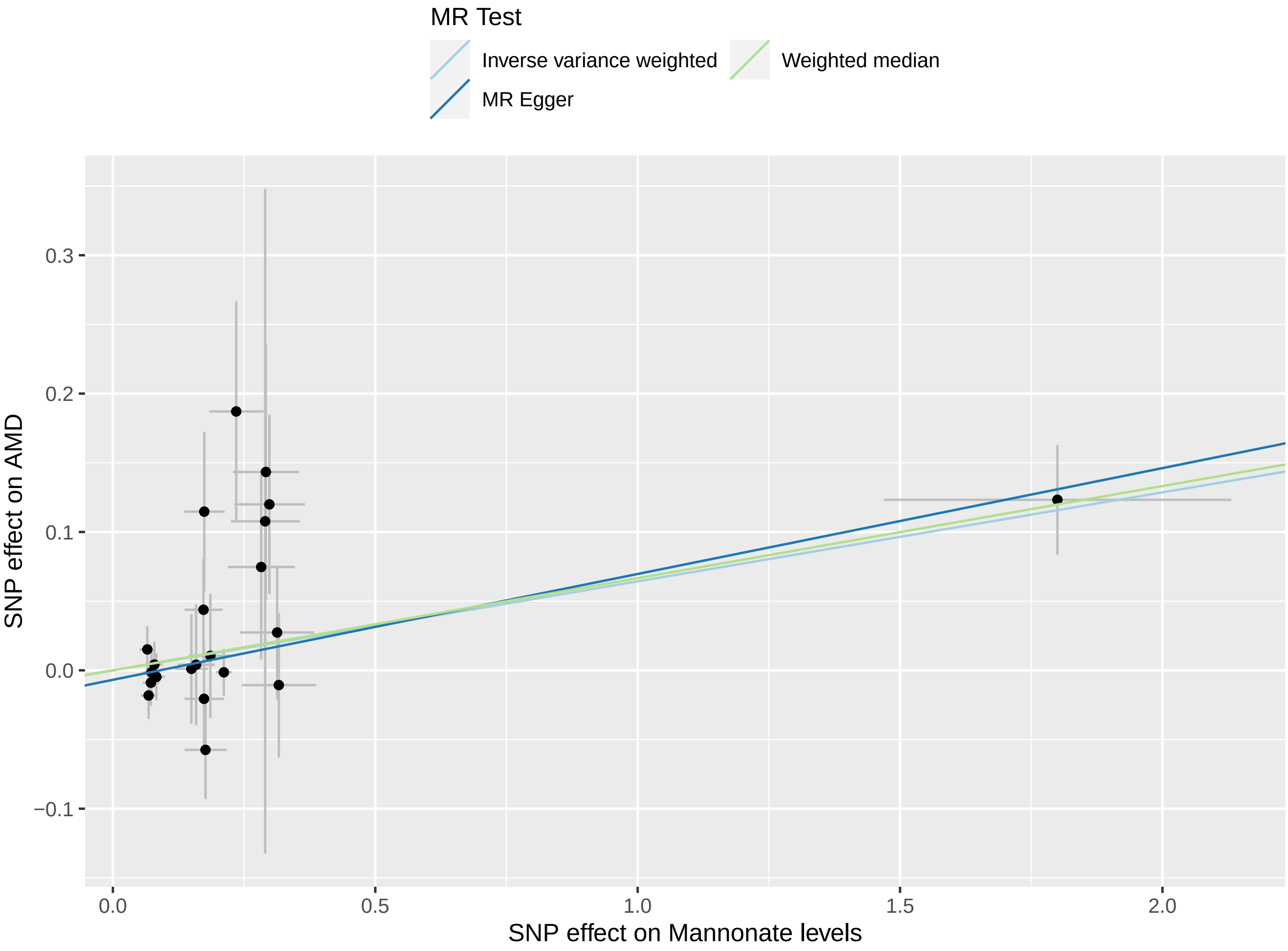
MR Test
Inverse variance weighted
Weighted median
MR Egger
SNP effect on AMD
SNP effect on Mannonate levels
0.3
0.2
0.1
0.0
−0.1
0.0
0.5
1.0
1.5
2.0

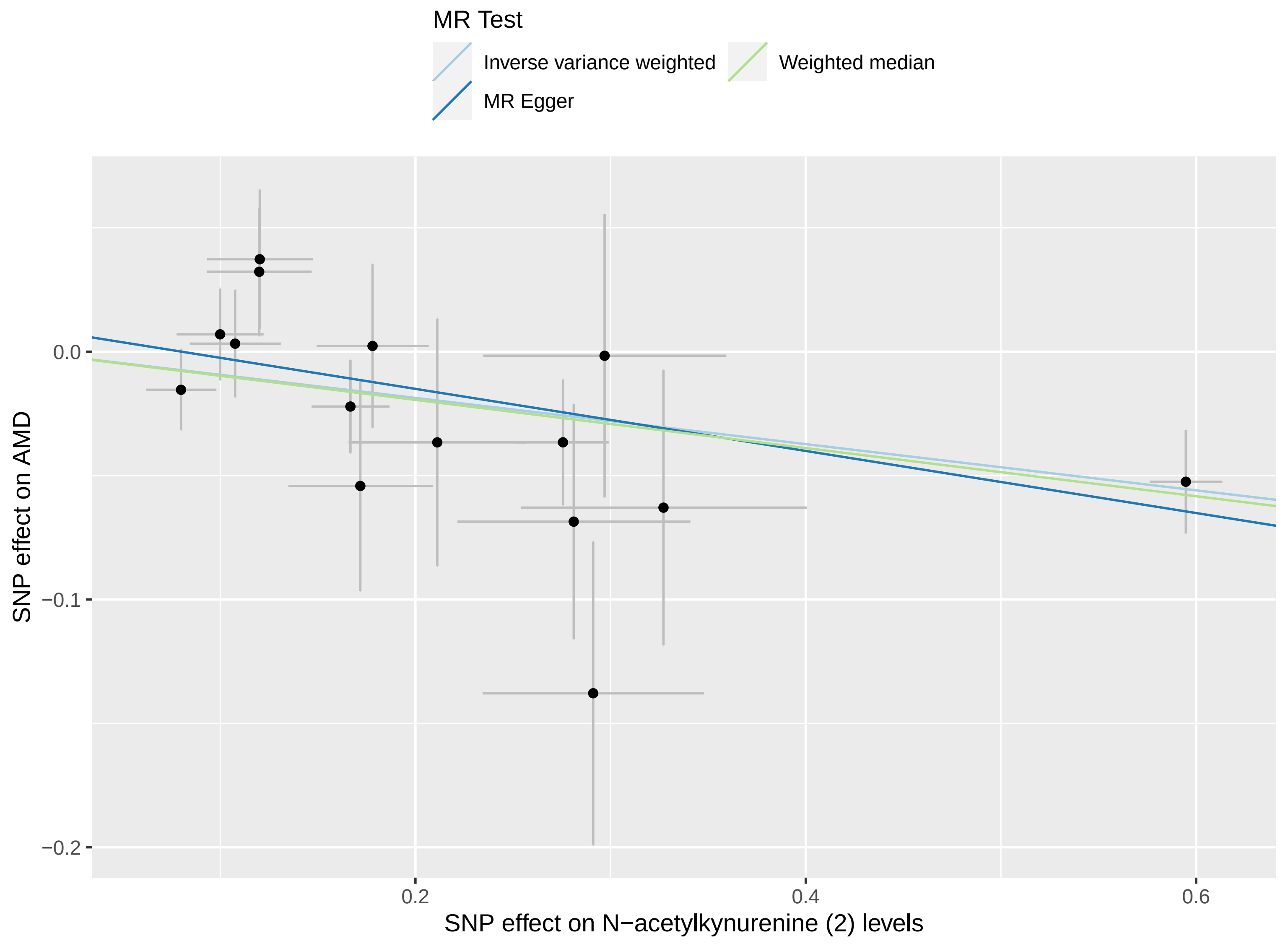
MR Test
Inverse variance weighted
Weighted median
MR Egger
0.0
−0.1
−0.2
SNP effect on AMD
0.2
0.4
0.6
SNP effect on N−acetylkynurenine (2) levels

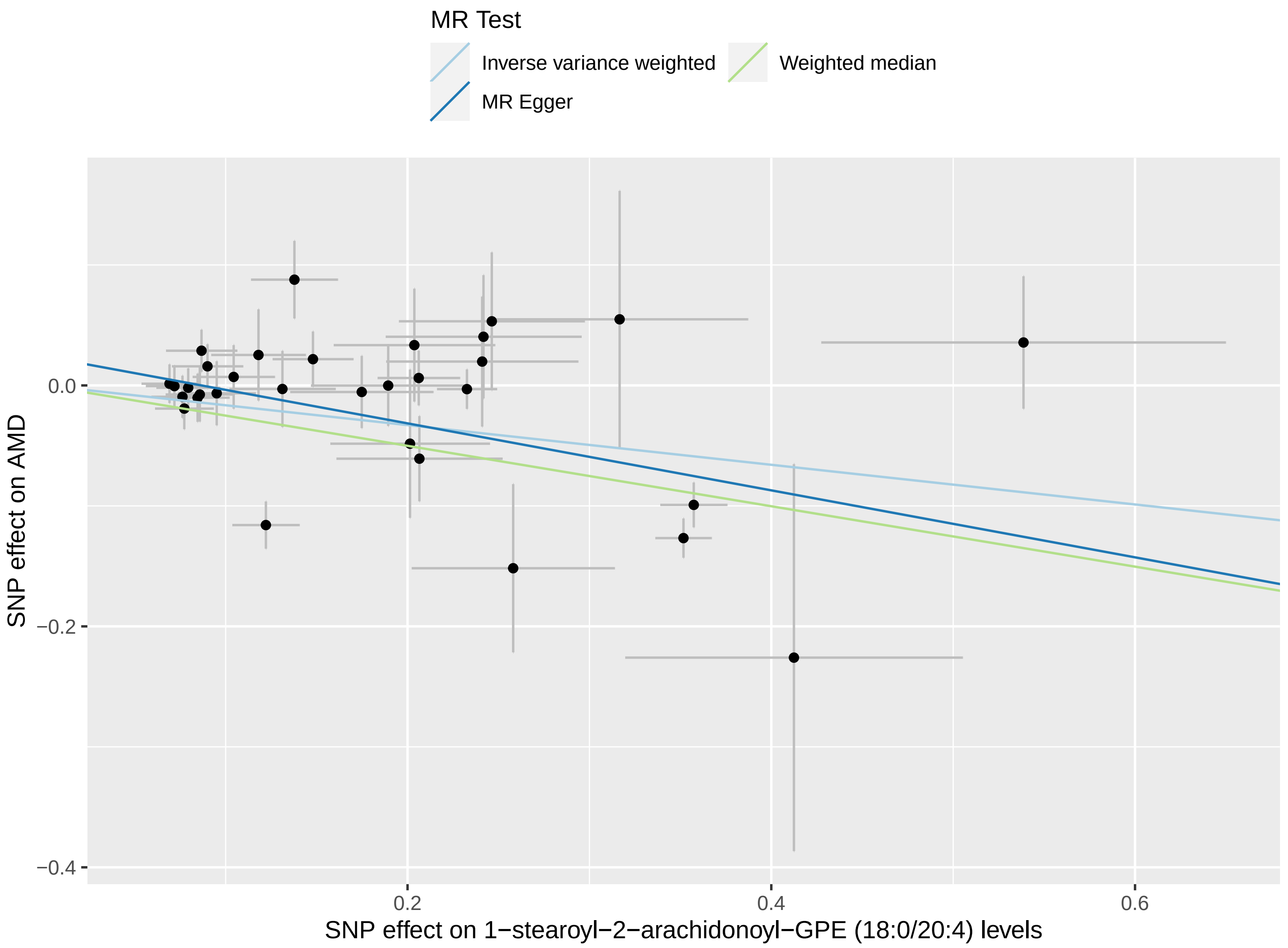
MR Test
Inverse variance weighted
Weighted median
MR Egger
0.0
−0.2
−0.4
SNP effect on AMD
0.2
0.4
0.6
SNP effect on 1−stearoyl−2−arachidonoyl−GPE (18:0/20:4) levels

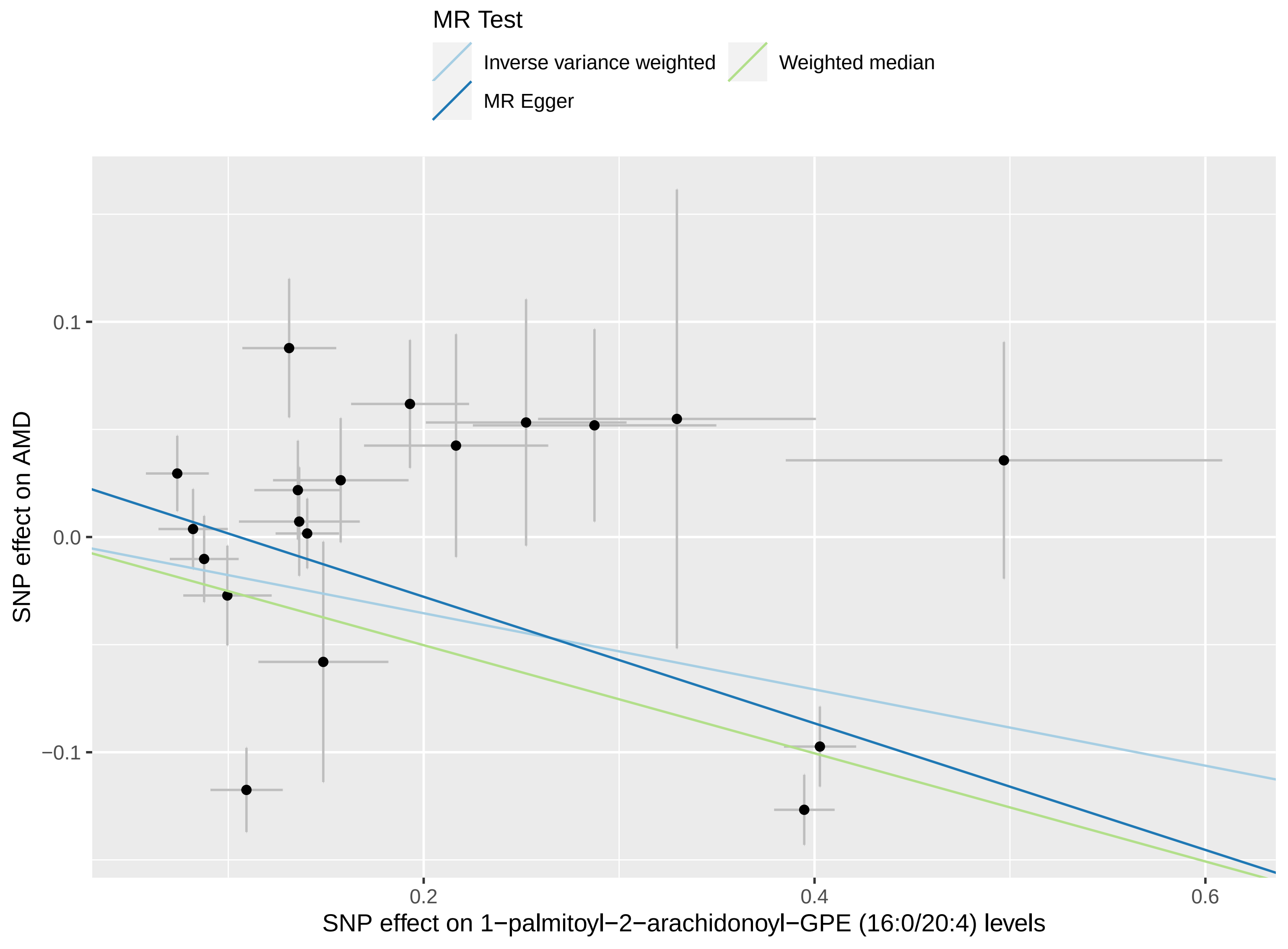

MR Test
Inverse variance weighted
Weighted median
MR Egger
0.1
0.0
−0.1
SNP effect on AMD
0.2
0.4
0.6
SNP effect on 1−palmitoyl−2−arachidonoyl−GPE (16:0/20:4) levels

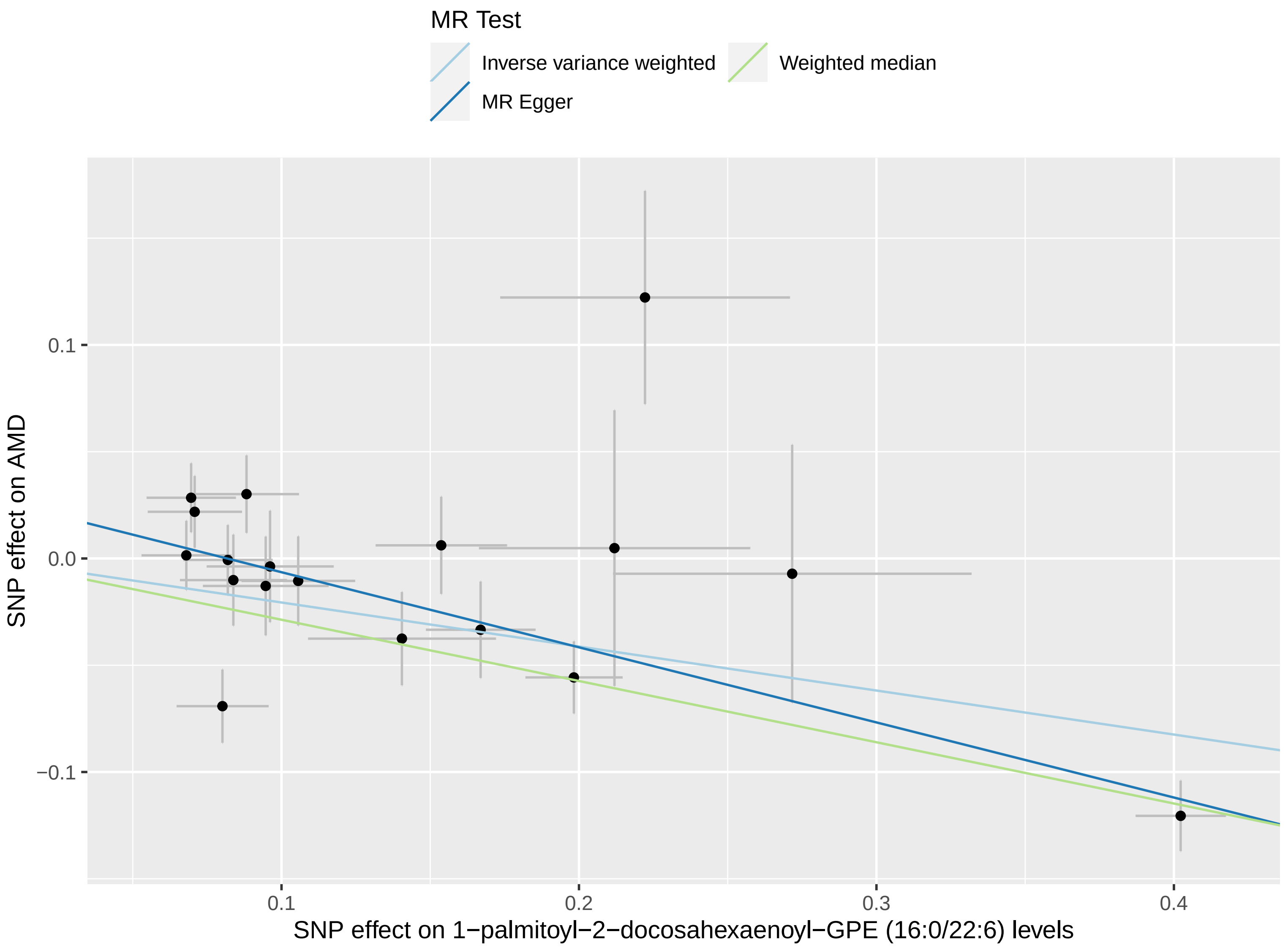
MR Test
Inverse variance weighted
MR Egger
Weighted median
SNP effect on AMD
0.1
0.0
−0.1
0.1
0.2
0.3
0.4
SNP effect on 1−palmitoyl−2−docosahexaenoyl−GPE (16:0/22:6) levels

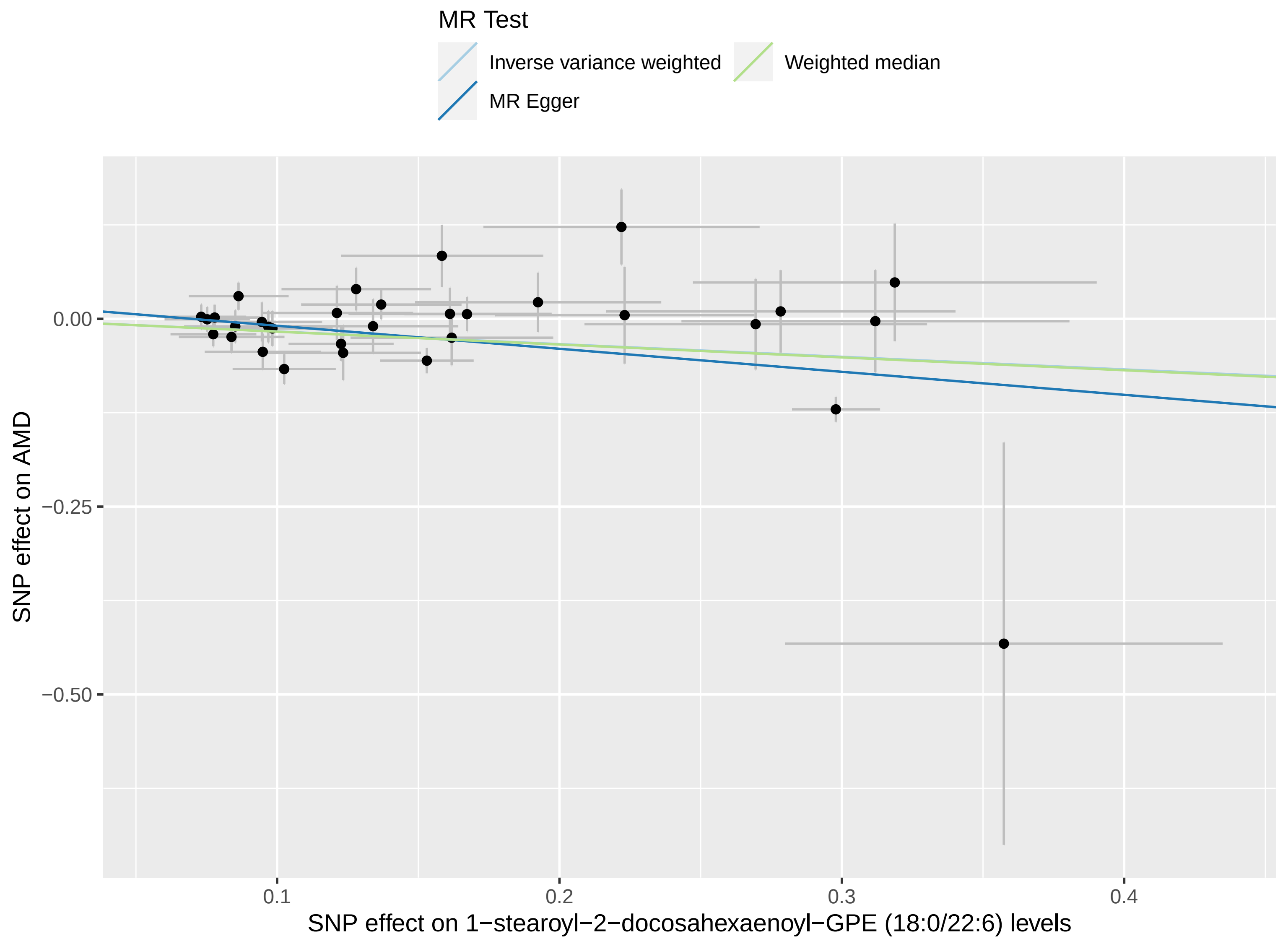

MR Test
Inverse variance weighted
Weighted median
MR Egger
0.00
−0.25
−0.50
0.1
0.2
0.3
0.4
SNP effect on AMD
SNP effect on 1−stearoyl−2−docosahexaenoyl−GPE (18:0/22:6) levels

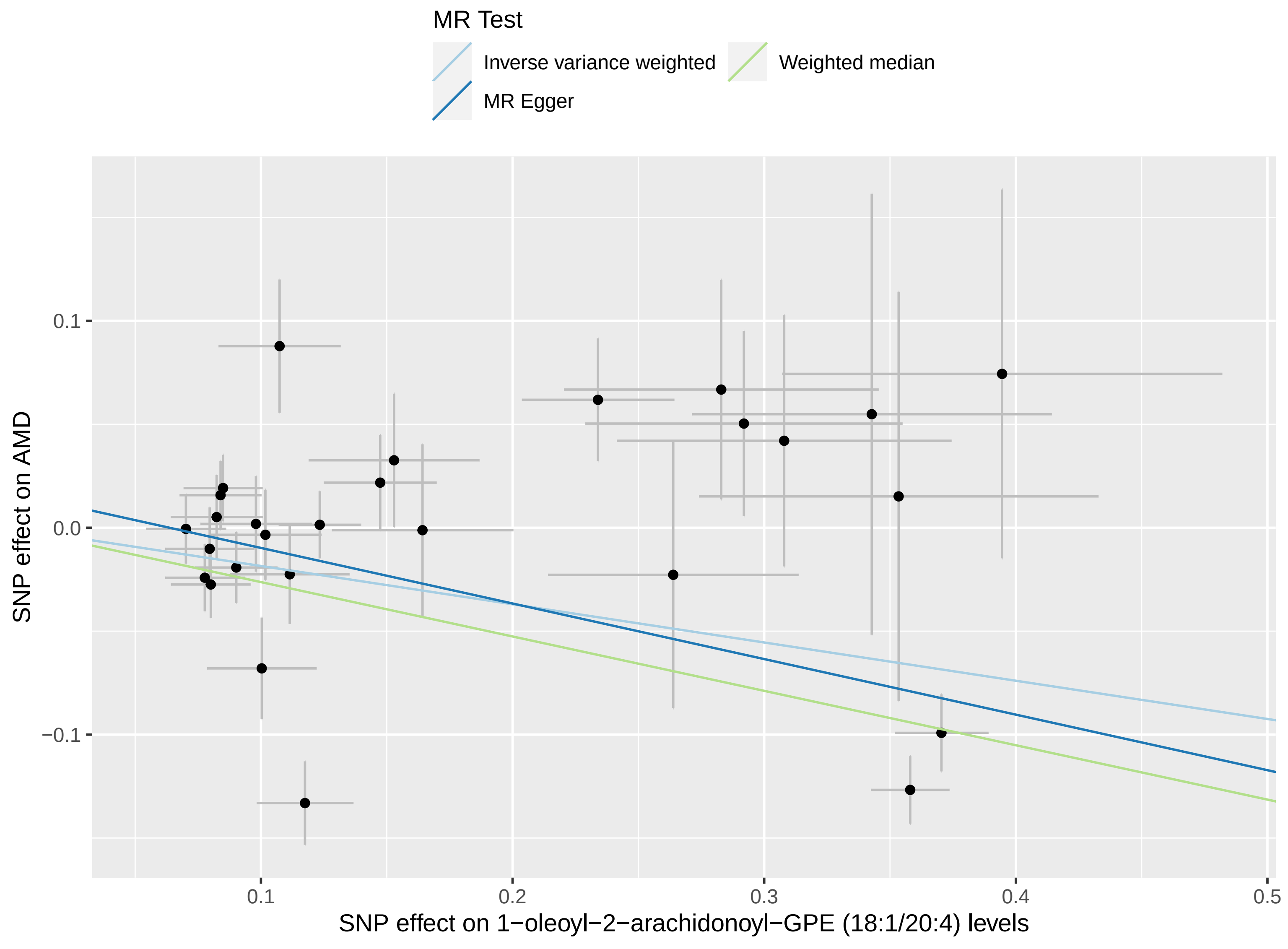
MR Test
Inverse variance weighted
MR Egger
Weighted median
SNP effect on AMD
0.1
0.0
−0.1
0.1
0.2
0.3
0.4
0.5
SNP effect on 1−oleoyl−2−arachidonoyl−GPE (18:1/20:4) levels

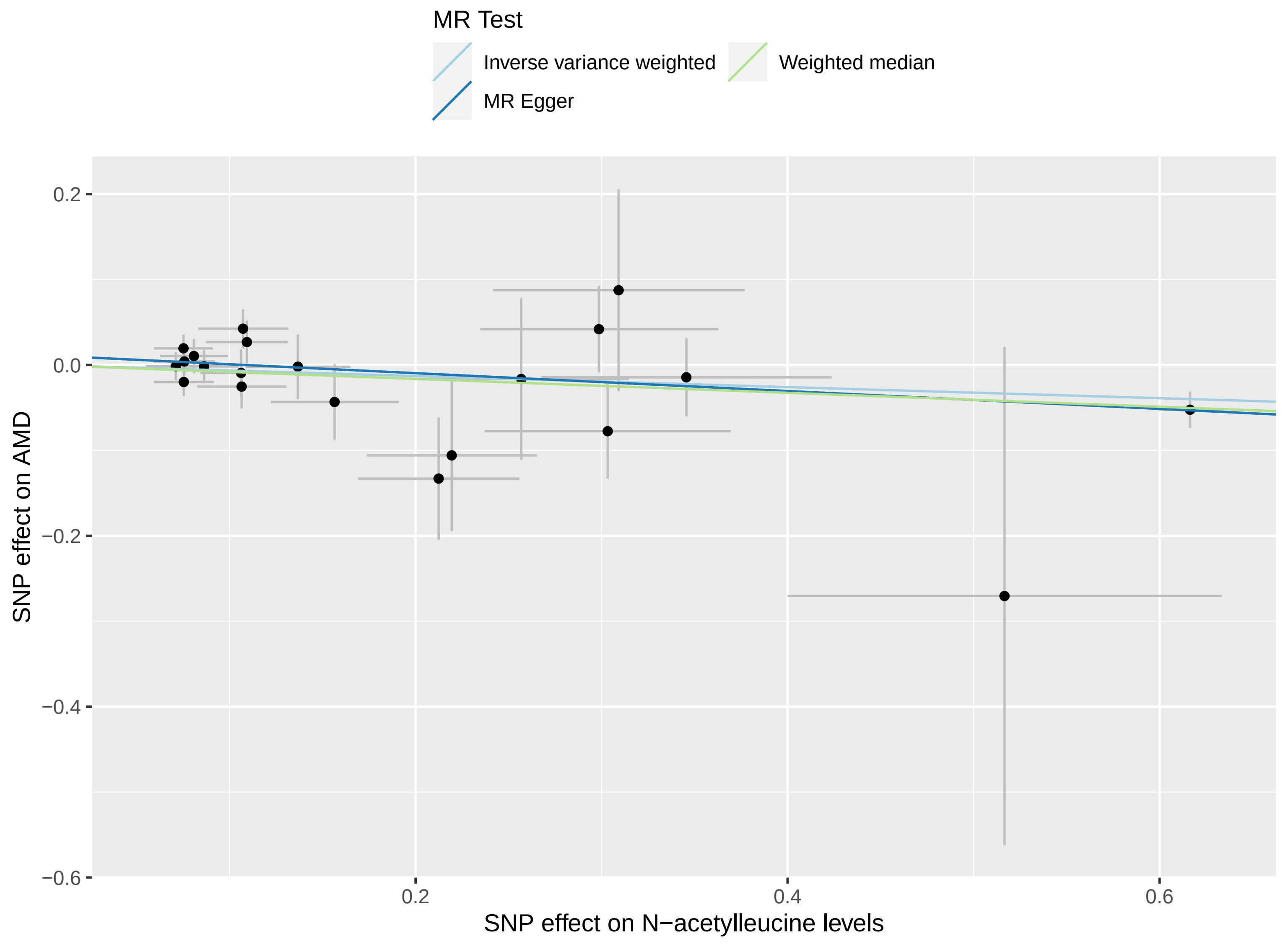

MR Test
Inverse variance weighted
MR Egger
Weighted median
0.2
0.0
−0.2
−0.4
−0.6
SNP effect on AMD
0.2
0.4
0.6
SNP effect on N−acetylleucine levels

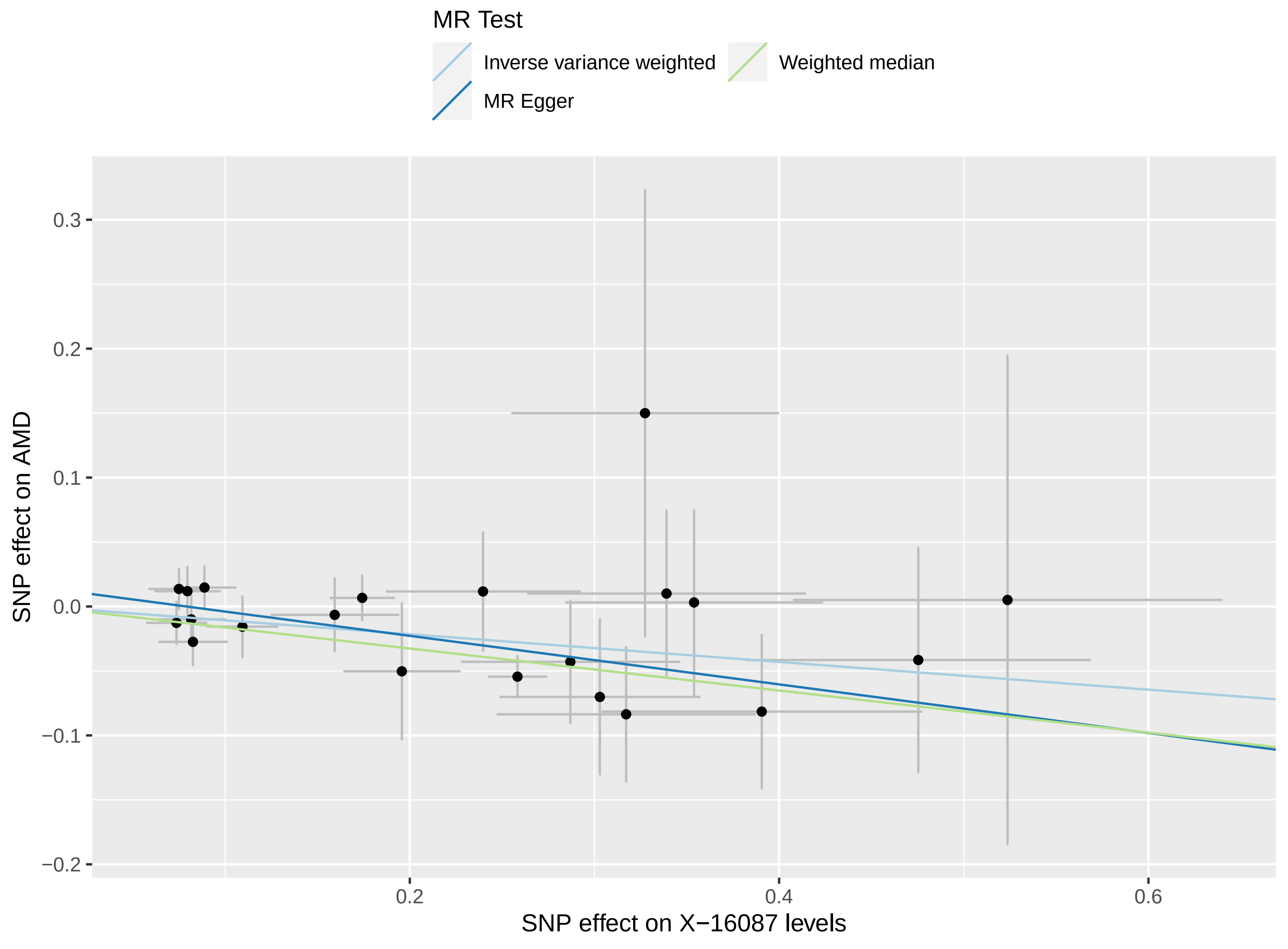
MR Test
Inverse variance weighted
Weighted median
MR Egger
0.3
0.2
0.1
0.0
−0.1
−0.2
SNP effect on AMD
0.2
0.4
0.6
SNP effect on X−16087 levels

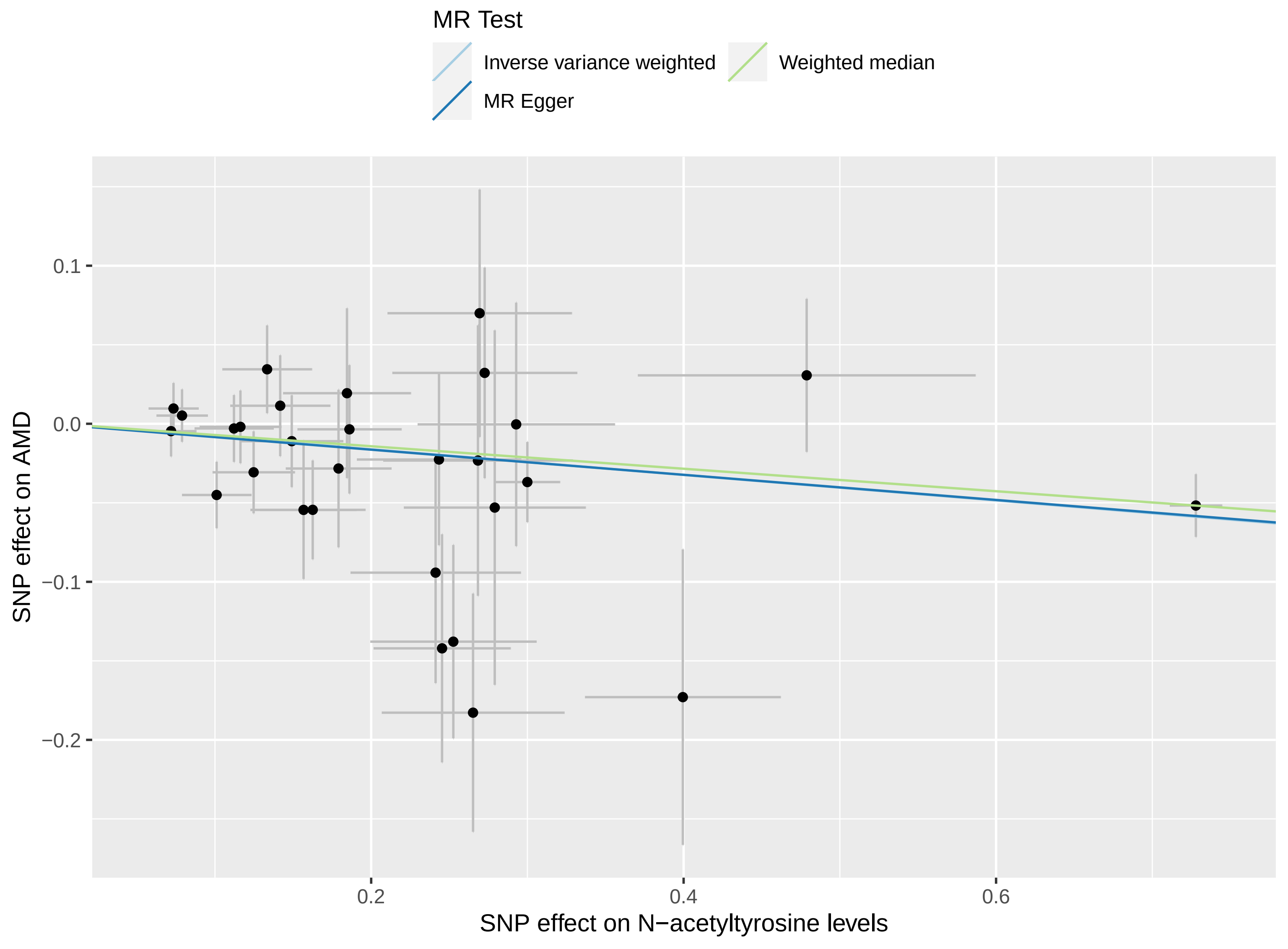

MR Test
Inverse variance weighted
Weighted median
MR Egger
SNP effect on AMD
0.1
0.0
−0.1
−0.2
0.2
0.4
0.6
SNP effect on N−acetyltyrosine levels

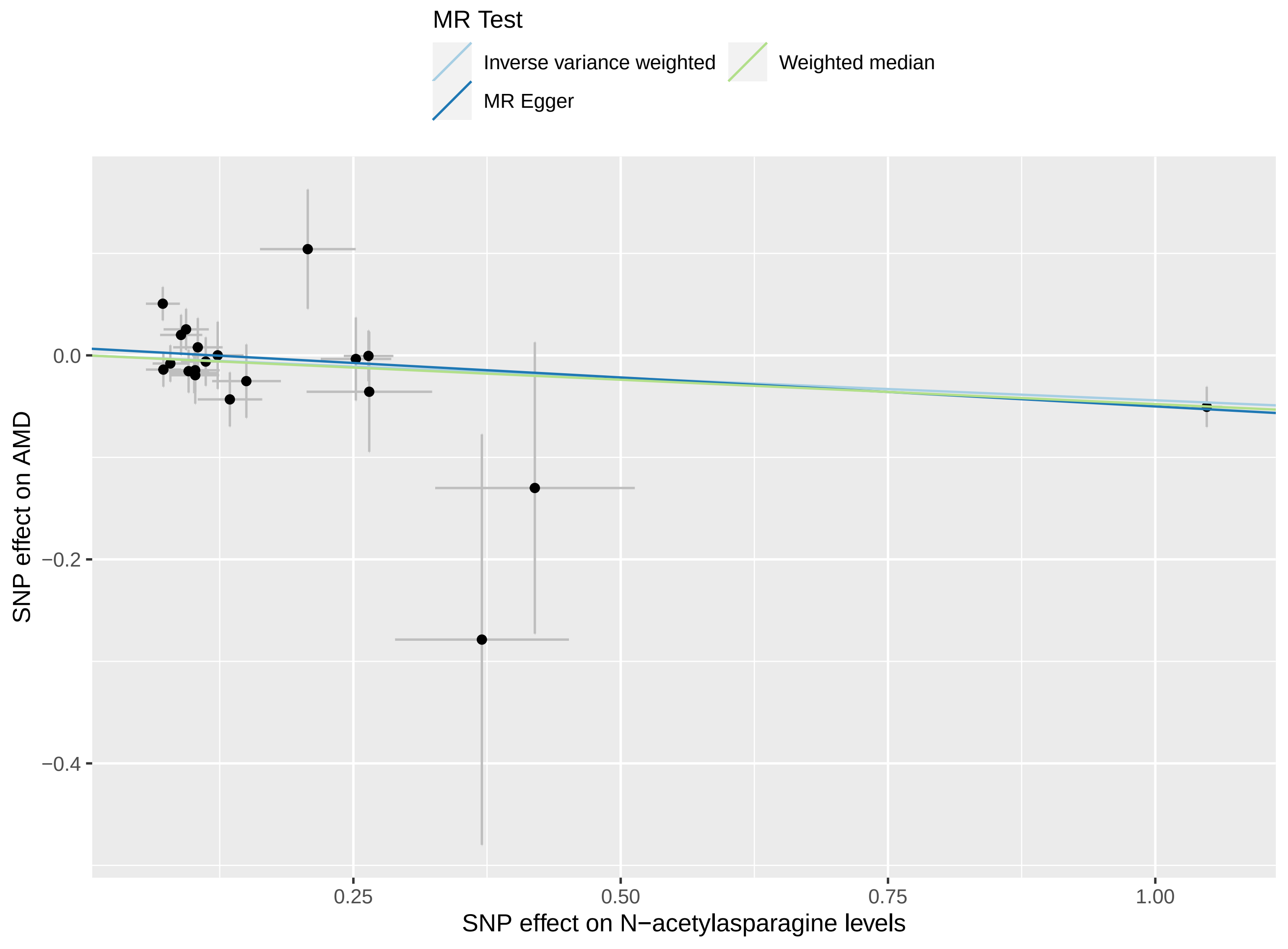
MR Test
Inverse variance weighted
Weighted median
MR Egger
0.0
−0.2
−0.4
0.25
0.50
0.75
1.00
SNP effect on AMD
SNP effect on N−acetylasparagine levels

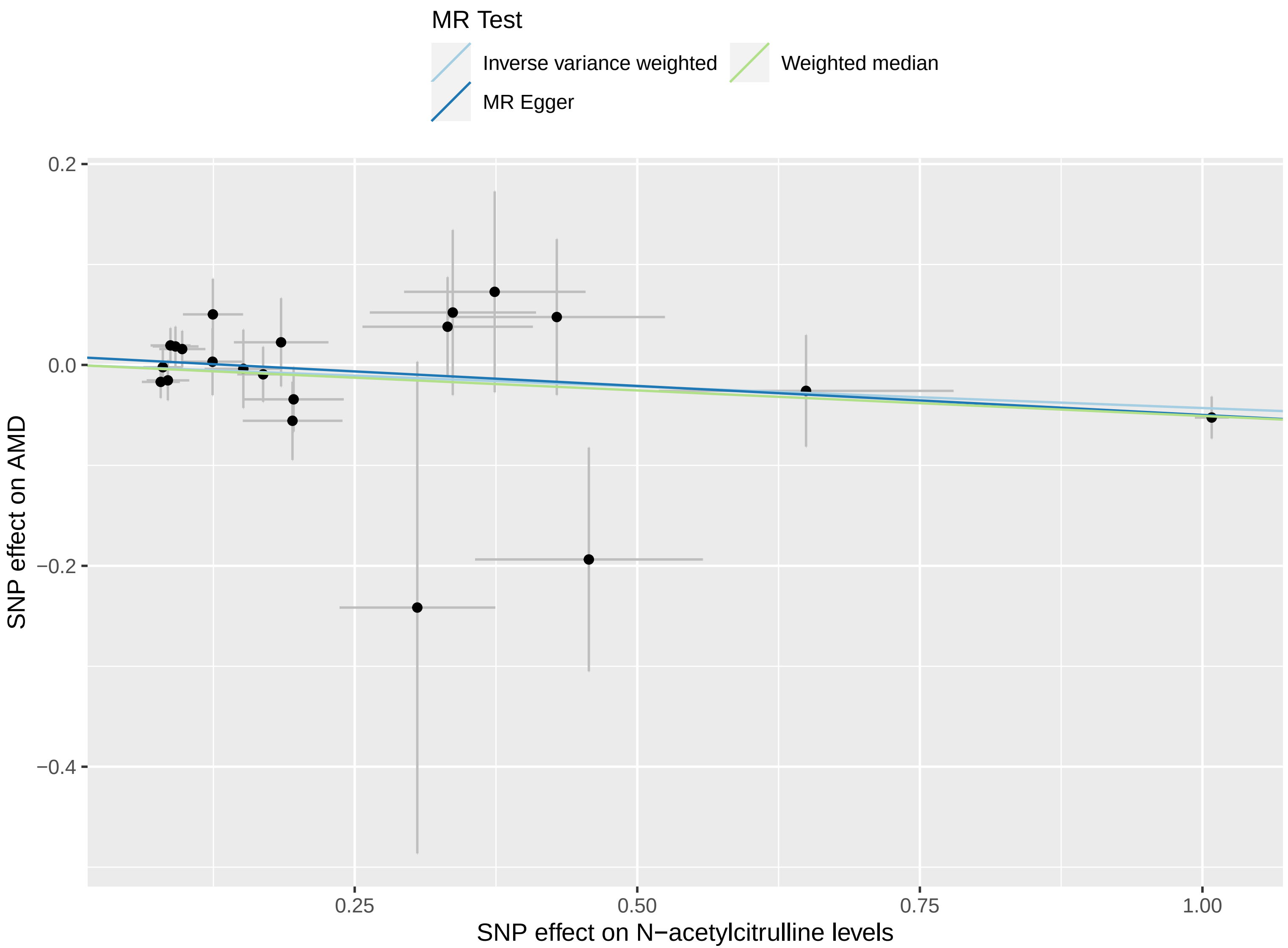

MR Test
Inverse variance weighted
MR Egger
Weighted median
0.2
0.0
−0.2
−0.4
SNP effect on AMD
0.25
0.50
0.75
1.00
SNP effect on N−acetylcitrulline levels

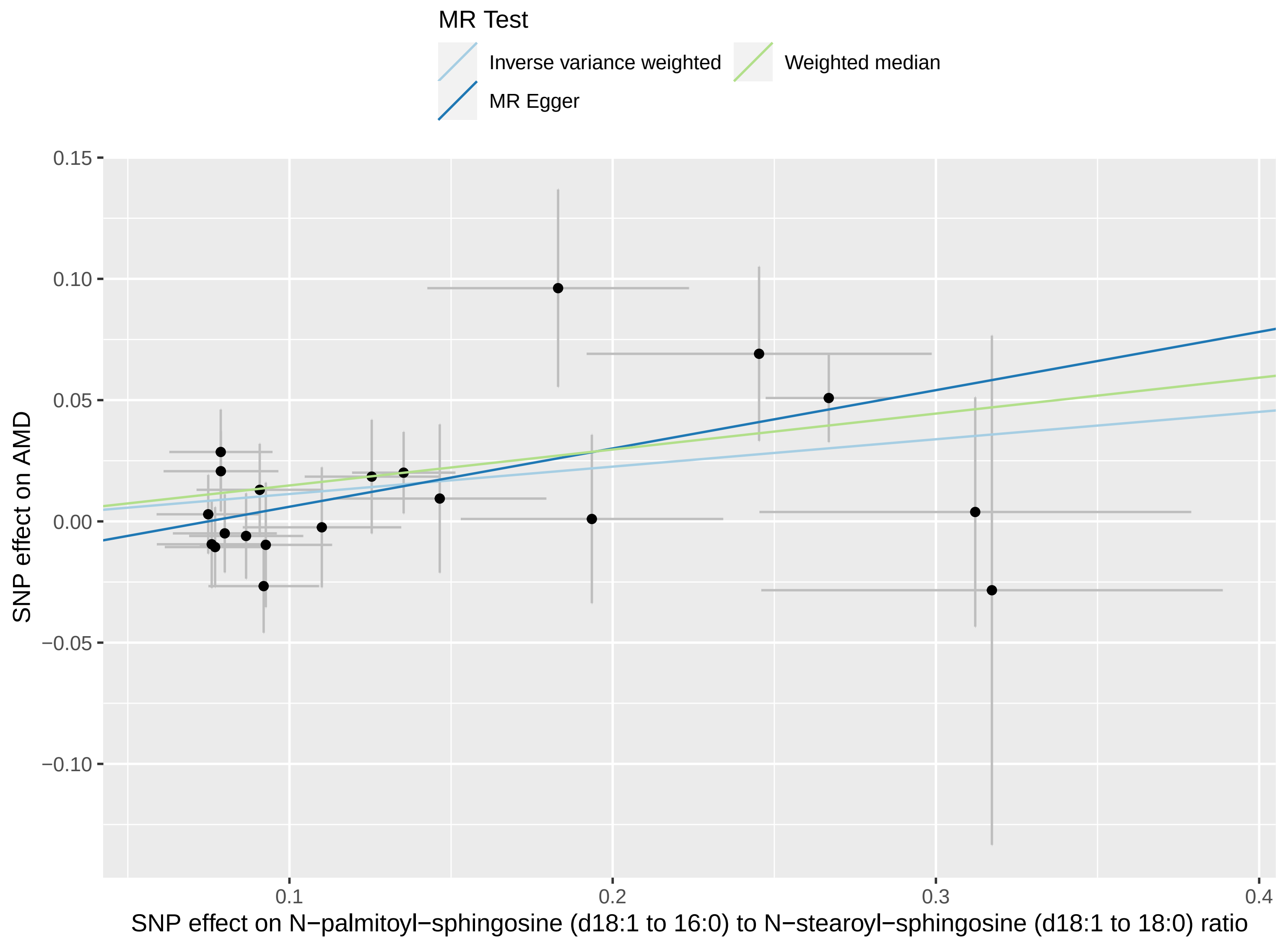
MR Test
Inverse variance weighted
Weighted median
MR Egger
0.15
0.10
0.05
0.00
−0.05
−0.10
SNP effect on AMD
0.1
0.2
0.3
0.4
SNP effect on N−palmitoyl−sphingosine (d18:1 to 16:0) to N−stearoyl−sphingosine (d18:1 to 18:0) ratio
